# Supplementary material for: Multi-level determinants of land use land cover change in Tigray, Ethiopia: A mixed-effects approach using socioeconomic panel and satellite data
Source: PLoS One. 2024 Jun 13;19(6):e0304896. doi: 10.1371/journal.pone.0304896 (PMC11175475; doi:10.1371/journal.pone.0304896)
Supplement: S1 Table — (DOCX) [file pone.0304896.s002.docx]

**S1 Table. Livelihood Zone of Tigray Region, Ethiopia, based on FEWS NET classification.** The Table Shows the location, main crops, and main livestock of each livelihood zone.

| **Livelihood Zone** | **Acronym** | **Administration Zone the LHZ mainly spans** | **Main Agro-ecology** | **Livelihood Zone Type** | **Main Crops** | **Main Livestock** |
| --- | --- | --- | --- | --- | --- | --- |
| Adiyabo Lowland | ALL | Western | Lowland | Cropping | sorghum, sesame, millet & maize | cattle & shoats |
| Alaje Ofla Highland | AOH | Southern | Highland | Cropping | wheat, pulses, barley & maize | cattle & shoats |
| Atsbi Womberta Highland | AWH | Eastern | Highland | Cropping | barley, wheat & pulses | shoats & cattle |
| Central Mixed Crop | CMC | Central | Mixed crop | Cropping | teff, wheat, barley & millet | shoats & cattle |
| Enderta Dry Midland | EDM | South-eastern | Dry midland | Cropping | barley, wheat, teff & pulses | cattle & shoats |
| Eastern Planteau | EPL | Eastern | Plateau | Cropping | barley, wheat & cactus fruit | shoats & cattle |
| Gesho and Wheat Highland | GWH | Southern | Highland | Cropping | gesho, wheat, pulses & teff | shoats & cattle |
| Humera Sesame and Sorghum | HSS | Western | Lowland | Cropping | sesame & sorghum | cattle & shoats |
| Southeast Rainfed Semi-mechanized agriculture | SRM | Western | Lowland | Cropping | sesame & sorghum | cattle & shoats |
| Irob Mountains | IRM | Eastern | Mountainous | Cropping | cactus fruit, barely, maiz and wheat | cattle & shoats |
| Mereb Basin | MRB | Northern-Western | Basin | Cropping | sorghum, millet, teff & groundnuts | cattle & shoats |
| Middle Tekeze | MTK | Northern-Western | Lowland | Cropping | sorghum, maize, teff & millet | shoats & cattle |
| Raya Valley | RVL | Southern | Valley | Cropping | sorghum, teff, maize & pulses | cattle & shoats |
| Tsirare Catchment | TSC | Western | Catchment | Cropping | sorghum, barley, wheat & pulses | shoats & cattle |
| West Central Teff | WCT | Central | Teff-producing | Cropping | maize, teff, sorghum & millet | shoats & cattle |
| Werie Catchment | WRC | Western | Catchment | Cropping | sorghum, maize, teff & barley | shoats & cattle |
| Western Cereal and Sesame | WSE | Western | Cereal and sesame-producing | Cropping | sesame, sorghum, nug & wheat | cattle & shoats |
