# Supplementary material for: Multi-level determinants of land use land cover change in Tigray, Ethiopia: A mixed-effects approach using socioeconomic panel and satellite data
Source: PLoS One. 2024 Jun 13;19(6):e0304896. doi: 10.1371/journal.pone.0304896 (PMC11175475; doi:10.1371/journal.pone.0304896)
Supplement: S2 Table — (DOCX) [file pone.0304896.s003.docx]

**S2 Table. LULC classification accuracy for 1986 and 2016.** The table shows the error matrix, user’s and producer’s accuracy, and overall accuracy for each class. The accuracy was based on Congalton and Green (1999), with 75 samples per class and equal sample sizes for all strata. The overall accuracies were 87% and 89%**.**

| Reference Data of 1986 | | | | | | | |
| --- | --- | --- | --- | --- | --- | --- | --- |
| **Classified Data** | **Crop land** | **Bare land** | **Forest land** | **Pasture land** | **Other land** | **Row Total** | **User Accuracy** |
| Crop land | 64 | 1 | 5 | 4 | 1 | 75 | 85.3 |
| Bare land | 3 | 65 | 1 | 2 | 4 | 75 | 86.7 |
| Forest land | 5 | 2 | 62 | 3 | 3 | 75 | 82.7 |
| Pasture land | 1 | 2 | 4 | 67 | 1 | 75 | 89.3 |
| Other land | 2 | 3 | 2 | 0 | 68 | 75 | 90.7 |
| Column Total | 75 | 73 | 74 | 76 | 77 | 375 |  |
| Producer Accuracy | 85.33% | 89.04% | 83.78% | 88.16% | 88.31% | 375 |  |
| **Overall Classification Accuracy** | **0.869333333** | | | | | | |
| **Reference Data of 2016** | | | | | | | |
| **Classified Data** | **Crop land** | **Bare land** | **Forest land** | **Pasture land** | **Other land** | **Row Total** | **User Accuracy** |
| Crop land | 70 | 0 | 2 | 2 | 1 | 75 | 93.3 |
| Bare land | 3 | 65 | 2 | 2 | 3 | 75 | 86.7 |
| Forest land | 3 | 2 | 67 | 1 | 2 | 75 | 89.3 |
| Pasture land | 1 | 2 | 6 | 64 | 2 | 75 | 85.3 |
| Other land | 2 | 4 | 1 | 0 | 68 | 75 | 90.7 |
| Column Total | 79 | 73 | 78 | 69 | 76 | 375 |  |
| Producer Accuracy | 88.61% | 89.04% | 85.90% | 92.75% | 89.47% | 375 |  |
| **Overall Classification Accuracy** | **0.8906666** | | | | | | |
