# Supplementary material for: Multi-level determinants of land use land cover change in Tigray, Ethiopia: A mixed-effects approach using socioeconomic panel and satellite data
Source: PLoS One. 2024 Jun 13;19(6):e0304896. doi: 10.1371/journal.pone.0304896 (PMC11175475; doi:10.1371/journal.pone.0304896)
Supplement: S3 Table — (DOCX) [file pone.0304896.s004.docx]

**S3 Table. Results of alternative regression models for land use allocation.** We compared results of the mixed-effect model for land allocation with the results of simple linear regression models (OLS) for each land use equations. The result show that mixed-effects model has the best fit and the lowest AIC value.

| **Land use share equation** | | **Cropland** | **Pasture** | **Fallow** | **Forest** | **Others** |
| --- | --- | --- | --- | --- | --- | --- |
| ***Plot level factors*** | Slope (%) | 0.0010 | (0.0005) | 0.0009 | 0.0005 | (0.0005) |
|  |  | (0.0007) | (0.0008) | (0.0012) | (0.0010) | (0.0008) |
|  | Elevation (m) | 0.0000 | 0.0000 | 0.0001 | 0.0000 | 0.0000 |
|  |  | 0.0000 | (0.0000) * | 0.0000 | 0.0000 | 0.0000 |
|  | Dist. to residence (kms) | 0.0003 | (0.0027) | 0.0020 | (0.0059) | (0.0060) |
|  |  | (0.0007) | (0.0089) | (0.0058) | (0.0088) | (0.0031) * |
|  | Soil type is vertosol (Yes=1) | (0.0147) | 0.0087 | 0.0889 | (0.0500) | (0.0274) |
|  |  | (0.0138) | (0.0222) | (0.0326) *** | (0.0251) * | (0.0194) |
|  | Soil quality: Fair | (0.0208) | 0.0150 | (0.0088) |  | (0.0816) |
|  |  | (0.0134) | (0.0152) | (0.0315) |  | (0.0186) *** |
|  | Soil quality: Poor | (0.0280) | (0.0105) | 0.0255 |  | (0.0986) |
|  |  | (0.0152) * | (0.0162) | (0.0351) |  | (0.0208) *** |
|  | Land tenure: Tenanted | 0.1047 | (0.0361) | (0.0840) |  | 0.2923 |
|  |  | (0.0315) *** | (0.0594) | (0.0826) |  | (0.0356) *** |
|  | Land tenure: Sharecropped | 0.0200 | 0.0022 | (0.0576) |  | 0.0493 |
|  |  | (0.0289) | (0.0452) | (0.0727) |  | (0.0407) |
| ***Household level factors*** | Literate house head (Yes=1) | (0.0244) | (0.0120) |  |  | 0.0149 |
|  |  | (0.0120) ** | (0.0147) |  |  | (0.0169) |
|  | Dependency ratio | (0.0160) | 0.0033 | 0.0290 |  | (0.0149) |
|  |  | (0.0059) *** | (0.0064) | (0.0123) ** |  | (0.0082) * |
|  | Ln of total area (hectare) | 0.0656 | 0.0659 | 0.1073 | 0.0433 | 0.0384 |
|  |  | (0.0046) *** | (0.0051) *** | (0.0106) *** | (0.0087) *** | (0.0088) *** |
|  | No of plots owned | (0.0056) | (0.0057) | (0.0167) | (0.0056) | (0.0285) |
|  |  | (0.0014) *** | (0.0014) *** | (0.0024) *** | (0.0022) ** | (0.0016) *** |
|  | Livestock (TLU) |  | (0.0040) |  |  |  |
|  |  |  | (0.0018) ** |  |  |  |
|  | Asset index | (0.0901) |  | (0.0391) |  | 0.1569 |
|  |  | (0.0487) * |  | (0.1400) |  | (0.0645) ** |
|  | Ln of annual income | 0.0032 |  |  | (0.0138) |  |
|  |  | (0.0035) |  |  | (0.0072) * |  |
|  | Dist. to nearest market (km) | (0.0005) | (0.0005) | (0.0025) | (0.0021) | 0.0005 |
|  |  | (0.0003) * | (0.0004) | (0.0008) *** | (0.0028) | (0.0004) |
|  | Dist. to Major Road (km) | 0.0006 | 0.0008 | 0.0012 | (0.0023) | (0.0019) |
|  |  | (0.0004) | (0.0007) | (0.0011) | (0.0037) | (0.0006) *** |
|  | Climate change perception index | (0.0291) | 0.0000 |  | (0.0332) | 0.0113 |
|  |  | (0.0157) * | (0.0001) |  | (0.0347) | (0.0219) |
| ***Community level factors*** | Average output price (Birr) | 0.0000 | (0.0004) |  | 0.0002 |  |
|  |  | 0.0000 | (0.0003) |  | (0.0001) *** |  |
|  | Population density (per km^2^) | 0.0000 | 0.0000 | 0.0000 | 0.0000 | 0.0000 |
|  |  | 0.0000 | 0.0000 | 0.0000 | 0.0000 | (0.0000) *** |
|  | Annual mean Rainfall (mm) |  | 0.0000 |  | (0.0002) |  |
|  |  |  | (0.0001) |  | (0.0005) |  |
|  | Prop. of agri. in 1 km buffer |  |  |  | (0.0547) |  |
|  |  |  |  |  | (0.1092) |  |
|  | Livelihood Zone: ALL | (0.0215) | (0.0231) | 0.0044 |  | (0.0860) |
|  |  | (0.0240) | (0.0348) | (0.0538) |  | (0.0334) ** |
|  | Livelihood Zone: CMC | 0.0351 | 0.0197 | (0.0640) | (0.2027) | (0.0496) |
|  |  | (0.0208) * | (0.0294) | (0.0589) | (0.1673) | (0.0301) * |
|  | Livelihood Zone: EDM | 0.0245 | 0.0451 | (0.0870) | (0.0693) | (0.0767) |
|  |  | (0.0237) | (0.0606) | (0.0874) | (0.1219) | (0.0330) ** |
|  | Livelihood Zone: EPL | (0.0562) | (0.0278) | (0.0552) | (0.1341) | 0.0950 |
|  |  | (0.0228) ** | (0.0225) | (0.0503) | (0.1144) | (0.0300) *** |
|  | Livelihood Zone: HSS | (0.0452) | 0.0073 | 0.0355 |  | (0.0866) |
|  |  | (0.0272) * | (0.0409) | (0.0629) |  | (0.0366) ** |
|  | Livelihood Zone: WCT | (0.0050) | (0.0175) | 0.0557 | (0.0684) | 0.0606 |
|  |  | (0.0235) | (0.0259) | (0.0541) | (0.1007) | (0.0324) * |
|  | Constant | 0.9945 | 0.3240 | 0.5882 | 0.7709 | 0.5448 |
|  |  | (0.0555) *** | (0.1042) *** | (1215) *** | (0.3408) ** | (0.0736) *** |
|  | *R*^2^ | 0.3200 | 0.7300 | 0.6000 | 0.7100 | 0.4300 |
|  | *N* | 916 | 123 | 196 | 57 | 919 |
|  | F | 16.64 *** | 11.11 *** | 12.26 *** | 5.22 *** | 29.71 *** |
|  | AIC | -803.2686 | -330.8000 | -159.5978 | -144.0090 | -178.2386 |
