# Supplementary material for: Multi-level determinants of land use land cover change in Tigray, Ethiopia: A mixed-effects approach using socioeconomic panel and satellite data
Source: PLoS One. 2024 Jun 13;19(6):e0304896. doi: 10.1371/journal.pone.0304896 (PMC11175475; doi:10.1371/journal.pone.0304896)
Supplement: S4 Table — (DOCX) [file pone.0304896.s005.docx]

**S4 Table. Land use land cover transition matrix by main LHZ and Tigray.** The table shows transitions among land cover categories as percent of total landscape of each LHZs (Tigray).

| **During 1986 to 2016** | **Livelihood Zone (LHZ)** | | | | | | | | | | | **Tigray** |
| --- | --- | --- | --- | --- | --- | --- | --- | --- | --- | --- | --- | --- |
|  | **ALL** | | | | | **CMC** | **EDM** | **EPL** | **HSS** | **WCT** | **OTH** |  |
| **Forest to** | (22.2,12.2) | | | | | (12.1,19.9) | (10.5,6.9) | (16.4,12.6) | (28.0,10.0) | (20.5,19.8) | (14.0,12.8) | (18.2,12.5) |
| **Forest** | **12.08** | | | | | **16.26** | **5.00** | **9.34** | **14.07** | **13.45** | **12.59** | **12.46** |
|  | *12.08* | | | | | *16.26* | *5.00* | *9.34* | *14.07* | *13.45* | *12.59* | *12.46* |
|  | 12.08 | | | | | 16.26 | 5.00 | 9.34 | 14.07 | 13.45 | 12.59 | 12.46 |
| **Pasture** | **2.42** | | | | | **0.08** | **0.11** | **0.23** | **5.86** | **0.88** | **0.85** | **1.95** |
|  | *7.04* | | | | | *0.07[0.01,0.1]* | *0.45* | *1.09* | *7.73* | *1.78* | *1.1* | *2.65* |
|  | 7.78 | | | | | 0.08[0.01,0.1] | 0.29 | 0.9 | 8.2 | 1.91 | 1.13 | 2.88 |
| **Cropland** | **0.78** | | | | | **1.1** | **3.75** | **3.35** | **1.54** | **2.55** | **1.99** | **1.99** |
|  | *3.11* | | | | | *2.57* | *4.23* | *6.03* | *6.34* | *5.85* | *3.82* | *4.87* |
|  | 2.33 | | | | | 1.64 | 2.48[1.28,0.5] | 3.43 | 3.35 | 3.13 | 2.11 | 2.68 |
| **Bare land** | **18.41** | | | | | **9.3** | **4.6** | **9.57** | **20.38** | **15.69** | **9.62** | **12.98** |
|  | *10.34[8.07,0.7]* | | | | | *7.65[1.65,0.2]* | *3.73[0.87,0.2]* | *6.20[3.37,0.5]* | *13.30[7.07,0.5]* | *10.79[4.90,0.4]* | *5.48[4.14,0.8]* | *7.97[5.01,0.6]* |
|  | 11.37[7.05,0.6] | | | | | 5.68[3.62,0.6] | 2.22[2.37,1.1] | 4.64[4.93,1.1] | 14.52[5.86,0.40] | 9.26[6.43,0.7] | 4.62[4.99,1.1] | 7.21[5.78,0.8] |
| **Others** | **0.62** | | | | | **1.64** | **2.03** | **3.22** | **0.25** | **1.41** | **1.54** | **1.32** |
|  | *1.75* | | | | | *1.82* | *2.08* | *3.05[0.17,0.1]* | *0.64* | *2.11* | *3.6* | *2.75* |
|  | 1.75 | | | | | 2.45 | 1.71[0.32,0.2] | 3.12[0.10,0.0] | 0.68 | 1.98 | 3.01 | 2.44 |
| **Pasture to** | | (4.5,21.0) | | | | (2.3,0.3**)** | (4.4,1.8) | (2.8,3.3) | (2.6,18.5) | (2.2,5.5) | (3.4,4.0) | (3.3,8.9) |
| **Forest** | **1.06** | | | | | **1.44** | **0.43** | **1.03** | **1.07** | **0.92** | **0.95** | **0.97** |
|  | *1.42* | | | | | *0.82[0.62,0.8]* | *0.54* | *0.64[0.39,0.6]* | *0.81[0.26,0.3]* | *0.77[0.14,0.2]* | *0.91[0.04,0.1]* | *0.93[0.03,0.0]* |
|  | 1.39 | | | | | 0.66[0.78,1.2] | 0.52 | 0.78[0.25,0.3] | 0.88[0.18,0.2] | 0.76[0.16,0.2] | 0.91[0.04,0.1] | 0.97 |
| **Pasture** | **3.01** | | | | | **0.13** | **1.99** | **1.84** | **2.48** | **0.32** | **1.83** | **2.03** |
|  | *3.01* | | | | | *0.13* | *1.99* | *1.84* | *2.48* | *0.32* | *1.83* | *2.03* |
|  | 3.01 | | | | | 0.13 | 1.99 | 1.84 | 2.48 | 0.32 | 1.83 | 2.03 |
| **Cropland** | **2.47** | | | | | **0.62** | **1.8** | **1.42** | **0.49** | **0.49** | **0.98** | **1.13** |
|  | *0.62[1.85,3.0]* | | | | | *0.31[0.31,1.0]* | *1.60[0.20,0.1]* | *0.84[0.57,0.7]* | *0.57* | *0.44[0.05,0.1]* | *0.73* | *0.75[0.38,0.5]* |
|  | 0.51[1.96,3.9] | | | | | 0.14[0.48,3.5] | 1.01[0.79,0.8] | 0.62[0.80,1.3] | 0.41[0.09,0.2] | 0.23[0.26,1.1] | 0.41 | 0.47[0.67,1.4] |
| **Bare land** | **0.09** | | | | | **0.13** | **1.39** | **0.13** | **0.84** | **0.47** | **0.35** | **0.5** |
|  | *2.06* | | | | | *0.91* | *1.4* | *0.87* | *1.21* | *0.82* | *1.04* | *1.22* |
|  | 2.47 | | | | | 0.48 | 0.91[0.48,0.5] | 0.83 | 1.77 | 0.69 | 0.9 | 1.26 |
| **Others** | **0.83** | | | | | **0.07** | **0.73** | **0.2** | **0.25** | **0.32** | **1.08** | **0.73** |
|  | *0.35[0.48,1.4]* | | | | | *0.22* | *0.79* | *0.43* | *0.06[0.19,3.2]* | *0.16[0.16,1.0]* | *0.69[0.40,0.6]* | *0.42[0.31,0.7]* |
|  | 0.38[0.45,1.2] | | | | | 0.21 | 0.70[0.03,0.0] | 0.56 | 0.08[0.16,2.0] | 0.15[0.17,1.2] | 0.59[0.50,0.9] | 0.43[0.31,0.7] |
| **Cropland to** | | | (8.0,5.9) | | | (9.2,4.7) | (5.6,11.2) | (12.9,9.0) | (3.9,6.8) | (12.1,7.0) | (6.9,6.3) | (7.1,7.0) |
| **Forest** | **6.15** | | | | | **8.95** | **3.45** | **9.27** | **2.75** | **10.84** | **6.12** | **5.84** |
|  | *2.17[3.98,1.8]* | | | | | *3.83[5.12,1.3]* | *1.04[2.42,2.3]* | *3.95[5.32,1.4]* | *1.12[1.62,1.5]* | *4.95[5.89,1.2]* | *2.19[3.93,1.8]* | *2.23[3.61,1.6]* |
|  | 2.35[3.80,1.6] | | | | | 4.99[3.96,0.8] | 2.44[1.01,0.4] | 5.52[3.75,0.7] | 2.46[0.29,0.1] | 7.22[3.61,0.5] | 3.66[2.46,0.7] | 3.65[2.19,0.6] |
| **Pasture** | **1.45** | | | | | **0.01** | **0.16** | **0.78** | **0.88** | **0.05** | **0.18** | **0.53** |
|  | *3.15* | | | | | *0.04* | *0.33* | *0.93* | *0.98* | *0.86* | *0.50[0.25,0.3]* | *0.97* |
|  | 4.18 | | | | | 0.05 | 0.57 | 1.14 | 2.77 | 1.35 | 0.89[0.56,1.4] | 1.89 |
| **Cropland** | **4.63** | | | | | **8.83** | **24.32** | **19.81** | **10.37** | **12.02** | **14.08** | **13.07** |
|  | *4.63* | | | | | *8.83* | *24.32* | *19.81* | *10.37* | *12.02* | *14.08* | *13.07* |
|  | 4.63 | | | | | 8.83 | 24.32 | 19.81 | 10.37 | 12.02 | 14.08 | 13.07 |
| **Bare land** | **0.23** | | | | | **0.12** | **1.37** | **1.00** | **0.09** | **0.47** | **0.18** | **0.33** |
|  | *3.15* | | | | | *4.28* | *2.73* | *5.34* | *1.68* | *5.22* | *2.51* | *2.92* |
|  | 4.18 | | | | | 3.61 | 4.30 | 5.89 | 4.91 | 6.56 | 3.64 | 4.74 |
| **Others** | **0.16** | | | | | **0.09** | **0.64** | **1.8** | **0.15** | **0.71** | **0.38** | **0.42** |
|  | *0.53* | | | | | *1.02* | *1.52* | *2.63* | *0.08[0.07,0.9]* | *1.02* | *1.65* | *1.01* |
|  | 0.64 | | | | | 1.55 | 3.30 | 3.96 | 0.23 | 1.4 | 2.37 | 1.6 |
| **Bare land to** | | | (26.8,18.8) | | | (19.0,10.2) | (14.0,9.2) | (13.0,12.6) | (23.5,21.3) | (19.5,17.0) | (16.9**,**11.3) | (19.5,14.7) |
| **Forest** | **4.3** | | | | | **9.42** | **2.71** | **2.01** | **6.13** | **7.99** | **5.25** | **5.34** |
|  | *10.03* | | | | | *11.51* | *2.43[0.28,0.1]* | *4.04* | *8.83* | *10.01* | *6.08* | *7.23* |
|  | 8.02 | | | | | 13.63 | 2.96 | 5.06 | 6.59 | 11.29 | 6.08 | 6.79 |
| **Pasture** | **17.07** | | | | | **0.14** | **1.45** | **2.15** | **11.64** | **4.48** | **2.79** | **6.26** |
|  | *9.90[7.18,0.7]* | | | | | *0.12[0.02,0.1]* | *0.77[0.68,0.9]* | *0.96[1.19,1.3]* | *7.69[3.95,0.5]* | *1.74[2.74,1.6]* | *1.40[1.39,1.0]* | *3.16[3.10,1.0]* |
|  | 9.77[7.31,0.8] | | | | | 0.13[0.01,0.1] | 0.69[0.76,1.1] | 1.05[1.10,1.1] | 7.44[4.20,0.6] | 2.11[2.37,1.1] | 1.48[1.31,0.9] | 3.53[2.74,0.8] |
| **Cropland** | **2.00** | | | | | **2.80** | **3.58** | **2.79** | **4.74** | **3.78** | **2.01** | **2.9** |
|  | *4.36* | | | | | *4.33* | *7.26* | *5.31[0.14,0.1]* | *6.31* | *5.72* | *4.87* | *5.8* |
|  | 2.93 | | | | | 2.84 | 5.79 | 3.99[0.47,0.5] | 3.04[1.70,0.6] | 3.46[0.32,0.1] | 2.77 | 3.28 |
| **Bare land** | **16.35** | | | | | **30.16** | **22.17** | **16.94** | **14.73** | **18.08** | **17.85** | **18.13** |
|  | *16.35* | | | | | *30.16* | *22.17* | *16.94* | *14.73* | *18.08* | *17.85* | *18.13* |
|  | 16.35 | | | | | 30.16 | 22.17 | 16.94 | 14.73 | 18.08 | 17.85 | 18.13 |
| **Others** | **3.38** | | | | | **6.66** | **6.29** | **6.04** | **0.96** | **3.29** | **6.89** | **4.98** |
|  | *2.46[0.92,0.4]* | | | | | *3.06[3.60,1.2]* | *3.57[2.72,0.8]* | *2.69[3.36,1.3]* | *0.64[0.32,0.5]* | *2.06[1.22,0.6]* | *4.58[2.310.5]* | *3.28[1.70,0.5]* |
|  | 2.20[1.18,0.5] | | | | | 4.25[2.42,0.6] | 3.99[2.31,0.6] | 3.63[2.41,0.7] | 0.62[0.35,0.6] | 2.19[1.10,0.5] | 3.93[2.96,0.8] | 2.99[1.99,0.7] |
| **Other uses to** | | | (1.5,5.0) | | | (0.9,8.5) | (4.2,9.7) | (3.8,11.3) | (0.2,1.6) | (0.7,5.7) | (3.2,9.9) | (2.3,7.5) |
| **Forest** | **0.71** | | | | | **0.03** | **0.3** | **0.25** | **0.05** | **0.06** | **0.52** | **0.37** |
|  | *0.40[0.32,0.8]* | | | | | *0.36* | *0.61* | *0.97* | *0.05* | *0.24* | *1.01* | *0.64* |
|  | 0.46[0.25,0.5] | | | | | 0.57 | 0.98 | 1.2 | 0.06 | 0.55 | 2.18 | 1.11 |
| **Pasture** | **0.01** | | | | | **0.03** | **0.06** | **0.18** | **0.1** | **0.06** | **0.21** | **0.14** |
|  | *0.39* | | | | | *0.00[0.03,6.7]* | *0.19* | *0.23* | *0.05[0.05,1.2]* | *0.04[0.02,0.5]* | *0.23* | *0.28* |
|  | 0.57 | | | | | 0.01[0.03,4.5] | 0.23 | 0.25 | 0.07[0.03,0.5] | 0.1 | 0.53 | 0.58 |
| **Cropland** | **0.69** | | | | | **0.22** | **2.07** | **1.41** | **0.05** | **0.17** | **1.3** | **0.94** |
|  | *0.17[0.52,3.0]* | | | | | *0.14[0.08,0.6]* | *1.82[0.25,0.1]* | *1.28* | *0.04[0.01,0.3]* | *0.14[0.030.2]* | *0.81[0.49,0.6]* | *0.52[0.43,0.8]* |
|  | 0.17[0.52,3.1] | | | | | 0.12[0.10,0.8] | 1.92[0.15,0.1] | 0.94 | 0.03[0.02,0.8] | 0.17[0.00,0.0] | 0.99[0.31,0.3] | 0.54[0.41,0.8] |
| **Bare land** | **0.11** | | | | | **0.63** | **1.8** | **1.94** | **0.02** | **0.38** | **1.19** | **0.84** |
|  | *0.57* | | | | | *0.41[0.23,0.6]* | *1.60[0.20,0.1]* | *1.31[0.63,0.5]* | *0.08* | *0.26[0.12,0.5]* | *1.16[0.02,0.0]* | *0.84* |
|  | 0.83 | | | | | 0.41[0.22,0.5] | 1.72[0.08,0.0] | 1.28[0.66,0.5] | 0.12 | 0.5 | 2.17 | 1.44 |
| **Others** | **0.97** | | | | | **1.13** | **7.79** | **3.3** | **0.14** | **1.14** | **9.27** | **3.87** |
|  | *0.97* | | | | | *1.13* | *7.79* | *3.3* | *0.14* | *1.14* | *9.27* | *3.87* |
|  | 0.97 | | | | | 1.13 | 7.79 | 3.3 | 0.14 | 1.14 | 9.27 | 3.87 |
| **Changes in the total landscape** | | | | | | | | | | | | |
| Gain (%) | 63.0 | | | | | 43.5 | 38.7 | 48.8 | 58.2 | 55.0 | 44.4 | 50.4 |
| Loss (%) | 63.0 | | | | | 43.5 | 38.7 | 48.8 | 58.2 | 55.0 | 44.4 | 50.5 |
| Net change (%) | | | | 20.0 | | 15.3 | 11.0 | 8.1 | 20.2 | 8.3 | 7.3 | 10.7 |
| Swamp (%) | | | 43.0 | | | 28.2 | 27.7 | 40.7 | 38.0 | 46.7 | 37.1 | 39.7 |
| Total Change (%) | | | | | 63.0 | 43.5 | 38.7 | 48.8 | 58.2 | 55.0 | 44.4 | 50.5 |
|  | | | | | | | | | | | | |

^*^Observed transitions in bold, expected loss in *italics* and expected gains in normal font under each LHZ (Tigray) as percent of total landscape in each LHZ (Tigray); observed transitions with numbers in square brackets next to their expected loss or/and gains are those with key signals of change; the first number in square brackets is actual percent of the landscape less the percent of the landscape that would be expected if the process of change were random, and the second number in square brackets is this difference divided by the expected percent of the landscape.

The first value in round brackets under each LHZs is gross loss and while the second number is gross gain each as percent of total landscape in each LHZ (Tigray).
