# Supplementary material for: Multi-level determinants of land use land cover change in Tigray, Ethiopia: A mixed-effects approach using socioeconomic panel and satellite data
Source: PLoS One. 2024 Jun 13;19(6):e0304896. doi: 10.1371/journal.pone.0304896 (PMC11175475; doi:10.1371/journal.pone.0304896)
Supplement: S1 Appendix — (DOCX) [file pone.0304896.s006.docx]

**S1 Appendix. Theoretical model and notation for smallholder farming household profit maximization**. We used the model and notation from Zhang et al., 2001 [1] to formulate the profit maximization problem for the smallholder farming households.

**Theoretical Model and Notation**

Economic theory postulates farming households, facing inexistence of markets for land, allocate their homogenous land to different uses according to the profitability of a specific land use. Land profitability directly or indirectly depends on output and input prices, physical and location conditions of land, socioeconomics and other natural environment factors [1–4]. Assume now landholder (farming household) wants to maximize profit from different land uses on homogenous land (plot^[[1]](#endnote-1)^) under his control by allocating it optimally between *k* different uses. Thus, a farming household’s profit maximization problem constrained by the total homogenous land area under his control can be presented as:

$$\underset{A^{k}}{Max} \pi\left( p^{k}, A \right)=\sum_{k=1}^{n} [p^{k}y^{k}(A^{k},E^{k},z)-cE^{k}]$$

*St* $\sum_{k=1}^{n} A^{k}\leq A$*,* $A^{k}$*,*$E^{k}\geq0$ (1)

Where $y^{k}$ is output for land use *k*, $p^{k}$ is farmgate price for output $y^{k}$, and $A$, $A^{k}$ , $E^{k}$ and $c$, respectively, are total plot area, land area allotted to *k*^th^ use (*k*=1, …, n) by each plot, aggregate input and unit cost of aggregate input used to produce output $y^{k}$. $z$ represent physical and location conditions of land, household socioeconomics and community level factors (like natural environment).

Assuming marginal product of land and aggregate input are positive but decreasing in each land use category i.e., $\frac{\partial y_{k}}{\partial A_{k}}>0, \frac{\partial^{2}y_{k}}{\partial{A_{k}}^{2}}<0 and \frac{\partial y_{k}}{\partial E_{k}}>0, \frac{\partial^{2}y_{k}}{\partial{E_{k}}^{2}}<0,$ we setup Lagrangean for the maximization as:

$L=\sum_{k=1}^{n} [p^{k}y^{k}(A^{k},E^{k},z)-cE^{k}]+\lambda(A-\sum_{k=1}^{n} A^{k})$ (2)

The first order conditions are, then,

$\frac{\partial L}{\partial A^{k}}=p^{k}\frac{\partial y^{k}}{\partial A^{k}}- \lambda\leq0\longrightarrow\left( p^{k}\frac{\partial y^{k}}{\partial A^{k}}- \lambda\right)A^{k}=0$ (3)

$\frac{\partial L}{\partial E^{j}}=p^{k}\frac{\partial y^{k}}{\partial E^{k}}- c\leq0\longrightarrow\left( p^{k}\frac{\partial y^{k}}{\partial E^{k}}- c \right)E^{k}=0$ (4)

$\frac{\partial L}{\partial\lambda}=\left( A-\sum_{k=1}^{n} A^{k} \right)\geq0\longrightarrow\left( A-\sum_{k=1}^{n} A^{k} \right)\lambda=0$ (5)

The marginal income from additional land to be used in Eq. (3) ($p^{k}\frac{\partial y^{k}}{\partial A^{k}}$) represent shadow land rents. Each household optimally allocate land in a way these land rents for a homogenous land (plot) are equal for each land use type. As implied by the first order condition, such optimal allocation of land area is, then, function of output price, aggregate input price, total plot area $(A)$ and other plot, household, and community level factors. Thus, land use/cover equations for the farming household considering plot owned can then be presented as:

$A^{k*}=A^{k*}(p^{k},c,z;A)$ (6)

**Reference**

1. Zhang Y, Uusivuori J, Kuuluvainen J. Econometric analysis of the causes of forest land use changes in Hainan, China. EEPSEA, Singapore, SG; 2001.

2. Bergeron G, Pender JL. Determinants of land use change: evidence from a community study in Honduras. 1999.

3. Djaenudin D, Oktaviani R, Hartoyo S, Dwiprabowo H. Modelling of land allocation behavior in Indonesia. Procedia Environ Sci. 2016;33: 78–86.

4. Zhao X, Pu J, Wang X, Chen J, Yang LE, Gu Z. Land-use spatio-temporal change and its driving factors in an artificial forest area in Southwest China. Sustainability. 2018;10: 4066.

1. Plot, a unit of analysis in this study, is piece of land owned (and managed) by a household in all the three waves. It may consist of several fields that can have different uses. That is plot can be allocated to different uses either in its entirety or in parts (a mixed land cover type). A household may own one or more plots in various locations and each plot is assumed to have similar characteristics, such as soil quality, size, location, or access to water and inputs. [↑](#endnote-ref-1)
