# Supplementary material for: Multi-level determinants of land use land cover change in Tigray, Ethiopia: A mixed-effects approach using socioeconomic panel and satellite data
Source: PLoS One. 2024 Jun 13;19(6):e0304896. doi: 10.1371/journal.pone.0304896 (PMC11175475; doi:10.1371/journal.pone.0304896)
Supplement: S2 Appendix — (DOCX) [file pone.0304896.s007.docx]

**S2 Appendix. Model specification and estimation procedure for land use allocation**. We specified a three-level mixed-effects model and estimated using maximum likelihood method.

**Empirical model and estimation**

For estimation purpose, this study assumed a linear specification for the land use/cover equations. Furthermore, in situations where price data for aggregate inputs are absent, their effects can be proxied by the effects of local population level and access to markets as local input prices are mostly affected by local demand such as land and labor [1]. Given plot-level three-wave panel dataset and these assumptions, land use/cover equations for each land use choice *k* (*k*=cropland, pasture, fallow, forest, and other land uses) on plot *i* in time *w* can be empirically specified as:

$A_{iw}^{k}=\alpha^{k}+\beta^{k}p_{w}^{k}+\phi^{k}{z_{iw}}^{k}+\psi^{k}z_{w}+\varepsilon_{iw}^{k}$ (1)

$A_{iw}^{k}$ is land area of the *i*^th^ plot allocated to *k*^th^ use in time *w*, $p_{w}^{k}$ is community level price of *k*^th^ land use output in time *w*, ${z_{iw}}^{k}$ is characteristics of *i*^th^ plot allocated to *k*^th^ land use in time *w*, and $z_{w}$ is household and community level factors in time *w*. $\alpha^{k}$, $\beta^{k}$, $\phi^{k}$, and $\psi^{k}$ are parameters to be estimated for the *k*^th^ land use whereas $\varepsilon_{iw}^{k}$ are correlated error terms [1–4].

Two possible sources for error term correlations can be noted (i) competition for plotland among different land uses or/and (ii) unobserved plot specific heterogeneity. Regarding the first, land use allocation considers tradeoff in land uses and resulting benefits. Tradeoff in land uses means that farmers must choose how to allocate their limited land area among different uses, and that each use has different benefits and costs^[[1]](#footnote-1)^. Hence any decision to use the land cannot be separated from each other i.e., the decision to increase the area of a land use will influence other land use decisions. This requires estimating land use/cover equations that can account for the correlation among errors of land use. Whereas unobserved plot heterogeneity in each land use/cover equations can be separated from the gross disturbance by adopting random/fixed effect specification in each equation [3–5].

To complete our specification, let’s have clear picture of the structure of our dataset. Our dataset consists of data on n plots of land that are grouped by their land use types. Each plot can have at least one of k possible land use types. We measure the dependent variables ($A_{iw}^{k}$) for each plot in each land use type in a vector (nx1)^[[2]](#footnote-2)^. The independent variables are arranged in a matrix (nxm) with block diagonal structure, meaning that each land use type has its own set of explanatory variables on the diagonal^[[3]](#footnote-3)^. We observe the plots over three waves, but not all plots are observed in all waves. Some plots may switch from one land use type to another, creating gaps in the data. This makes our panel data unbalanced^[[4]](#footnote-4)^. To account for this, we follow the method of Biorn et al. [6] and group the plots by the number of waves they are observed in. This reflects the degree of sustainability of a plot in a land use type. A plot that stays in the same land use type for all three waves^[[5]](#footnote-5)^ is more sustainable than a plot that switches. Therefore, our dataset has a hierarchical structure: plots are nested within sustainability groups, and sustainability groups are nested within land use types.

We used unbalanced panel data to fit land use/cover equations that account for the potential correlation of errors and the plot specific effects that are not observed. For this purpose, we respecify Eq. (1) as a three-level mixed-effects model as follows:

$A_{ijk}=\alpha_{k}+\phi z_{ijk}+c_{ij}+c_{ijk}+\mu_{ijk}$ (2)

Where $A_{ijk}$is the area of land allocated to use (*k*) by plot (*i*) in sustainability group (*j*), $z_{ijk}$ is a vector of determinants (plot, household and community level factors like price of outputs of *k*^th^ land use) for land use (*k*) by plot (*i*) in sustainability group (*j*), $\alpha_{k}$ is the intercept for land use group (*k*), $\phi$ is a vector of fixed effects coefficients, $c_{ij}$ is a random intercept for sustainability group (*j*) within land use type (*k*), $c_{ijk}$ is a random intercept for plot (*i*) within sustainability group (*j*) and land use type (*k*), and $\mu_{ijk}$ is the error term with $\mu_{ijk}\sim IIN(0,{\sigma^{2}}_{u})$. The random intercepts are uncorrelated with error terms and assumed to be normally distributed with mean zero and variance-covariance matrices to be estimated [7,8].

The fixed effects coefficient $\phi$ measures the average effect of the determinants on the land use area across all plots, sustainability groups, and land use types. The random effects coefficients $c_{ij}$, and $c_{ijk}$ capture the unobserved heterogeneity at different levels of the hierarchy, such as sustainability group and plot.

We assume that the vector of error terms follows $\mu\sim N(0,\Omega)$, which captures the possible correlation between the errors of different land use equations. The error covariance matrix has the following form:

$$\Omega=\left[ \begin{matrix} \sigma_{1}^{2} & \cdots& \sigma_{lk} \\ \vdots& \ddots& \vdots\\ \sigma_{kl} & \cdots& \sigma_{k}^{2} \end{matrix} \right]$$

where $\Omega$ is the error covariance matrix, $\sigma_{k}^{2}$^​^ ​ is the variance of the error term for land use type (*k*), and $\sigma_{kl}$ is the covariance of the error terms for land use types (*k*) and (*l*). Note that $\Omega$ is symmetric and positive definite.

We estimated the parameters of our three-level mixed-effects model using the maximum likelihood method. We used Stata version 17 to perform the estimation. We suppressed the constant term for the whole model by using the `noc` option in the `mixed` command, as we were interested in the constant of each land use equation, which we obtained by adding a column vector of 1 in the vector of determinants arranged as a block diagonal matrix. We also suppressed the random intercept for the land use group by using the `noc` option after the `*k*` level of the model specification, as it was captured by the estimated constant for each land use equation. We obtained the estimates of the fixed effects coefficients, the random effects variances, and the error covariance matrix from the output of the `mixed` command. Ω was estimated by specifying an unstructured covariance matrix for the error terms using `residuals (unstructured)`option in the `mixed` command to allow us account for the possible correlation between the errors of different land use equations. We also performed post-estimation tests and diagnostics to check the validity and accuracy of our model [9]..

**Reference fl’t;rhlt[d’yhljl’**

1. Bergeron G, Pender JL. Determinants of land use change: evidence from a community study in Honduras. 1999.

2. Verburg PH, Van Eck JRR, de Nijs TC, Dijst MJ, Schot P. Determinants of land-use change patterns in the Netherlands. Environ Plan B Plan Des. 2004;31: 125–150.

3. Zhang Y, Uusivuori J, Kuuluvainen J. Econometric analysis of the causes of forest land use changes in Hainan, China. EEPSEA, Singapore, SG; 2001.

4. Zhou B, Kockelman KM. Lessons learned in developing and applying land use model systems: parcel-based example. Transp Res Rec. 2009;2133: 75–82.

5. Chomitz KM, Gray DA. Roads, land use, and deforestation: a spatial model applied to Belize. The Economics of Land Use. Routledge; 2017. pp. 289–314.

6. Biørn E, Lindquist K-G, Skjerpen T. Heterogeneity in returns to scale: a random coefficient analysis with unbalanced panel data. J Product Anal. 2002;18: 39–57.

7. Carlson BZ, Georges D, Rabatel A, Randin CF, Renaud J, Delestrade A, et al. Accounting for tree line shift, glacier retreat and primary succession in mountain plant distribution models. Divers Distrib. 2014;20: 1379–1391. doi:10.1111/ddi.12238

8. Galvanin EAS, Menezes R, Pereira MHX, Neves SMAS. Mixed-effects modeling for analyzing land use change in the Brazilian Pantanal subregion of Cáceres. Remote Sens Appl Soc Environ. 2019;13: 408–414. doi:10.1016/j.rsase.2018.12.008

9. West BT, Welch KB, Galecki AT. Linear mixed models: a practical guide using statistical software. Crc Press; 2022.

1. For example, crop production may have higher revenue but also higher input costs and environmental degradation than pasture production. Tradeoff in land uses, then, implies there is interaction or spillover effect among outputs, such as opportunity cost, income effect, or environmental impact. [↑](#footnote-ref-1)
2. The dependent variables for each kth land use are stacked into a single vector (nx1) of dependent variables. [↑](#footnote-ref-2)
3. Such data structure is intentionally done by careful data manipulations (preparation) to trick Stata in to estimating a system of land use equations apparently as a single equation. This is because with time invariant plot level factors, adopting seemingly unrelated regression (SUR) was not feasible. [↑](#footnote-ref-3)
4. If a plot (in part or in its entirety) enter a land use category at the first wave and switch in its entirety to other land use, it may be back to its original land use in the next wave creating gap in waves in the land use category. [↑](#footnote-ref-4)
5. The logic behind adopting the method of Biorn et al. (2003) is to account for the unbalancedness of the panel data and to capture the degree of sustainability of a plot in a land use type. By grouping the plots by the number of waves they are observed in, we can compare the plots that are more stable (observed in all three waves) with those that are less stable (observed in one or two waves). This allows us to test how different factors affect the sustainability of plot in a land use. A plot (in part or its entirety) will sustain all the three-waves in a land use category if there are favorable household (like capital availability) or/and community-level features (environmental factors), if not, lesser level of sustainability (in two-waves or in a single-wave). For example, capital constraints may prevent farmers from investing in productivity-enhancing technologies and inputs, such as improved seeds, fertilizers, irrigation, etc. This leads to low yields and low returns from agriculture, which discourages farmers and make them leave some land fallow or clear more land from forests to expand their cultivation area, hoping to increase their output and income. [↑](#footnote-ref-5)
