# Supplementary material for: Multi-level determinants of land use land cover change in Tigray, Ethiopia: A mixed-effects approach using socioeconomic panel and satellite data
Source: PLoS One. 2024 Jun 13;19(6):e0304896. doi: 10.1371/journal.pone.0304896 (PMC11175475; doi:10.1371/journal.pone.0304896)
Supplement: S3 Appendix — (DOCX) [file pone.0304896.s008.docx]

**S3 Appendix. Land use land cover detection techniques.** We used growth rate and transition matrix analysis to detect the land use land cover changes in the study area.

**Diagnostic detection techniques**

Our change detection analysis began with visual observation (LULC map and graphs) of land cover of the two periods (1986 and 2016). Spatiotemporal land cover distribution for each LHZ and Tigray as whole were analyzed in terms of percentages of total area of each landscape in each period to visually identify the changes that are associated with LULCC in each landscape. Moreover, a growth rate-based change detection diagnosis was also adopted to quantify LULC change relative to the reference period, 1986. Average annual rate of LULC change between study periods was calculated as:

$R_{c}=\frac{1}{{(t}_{2}-t_{1)}}*ln \left( \frac{A_{2}}{A_{1}} \right)*100\%$ (1)

Where $R_{c}$ is an average annual rate of change (%); $A_{1}$ is amount of land cover (hectare) in time 1986; $A_{2}$ is an amount of land cover (hectare) in time 2016; and $t_{2}$ and $t_{1}$ are the time two study years.

**Transition matrix method**

Transition matrix is a technique to compare LULC of a landscape at two different time periods, providing comprehensive and detailed “from-to” LULC change information without requiring data normalization between the two dates (Table 1). It organizes categories of the initial time at the rows and categories of the subsequent period at the columns. It displays the proportion of the observed landscape that do not experience any change ($P_{jj}$) on the diagonal of the matrix, whereas proportions of the observed landscape that experience change at the off diagonals. Entries off the diagonal indicate a transition from category *i* to a different category *j*. The column sum of each category during the first period ($P_{i+}$) denotes the proportion of the landscape in category *i* in the initial time while the row total of each category during the subsequent time ($P_{+j}$) denotes the proportion of landscape in category *j* in the subsequent time.

Table 1. Transition matrix for comparing two maps in different time periods.

|  | Subsequent time | | | | Initial time total |
| --- | --- | --- | --- | --- | --- |
|  | Category 1 | Category 2 | … | Category *j* |  |
| Initial time |  | | | | |
| Category 1 | *P_11_* | *P_12_* |  | *P_1j_* | *P_1+_* |
| Category 2 | *P_21_* | *P_22_* |  | *P_2j_* | *P_2+_* |
| … | … | … | … | … | … |
| Category *i* | *P_i1_* | *P_i2_* |  | *P_jj; i=j_* | *P_i+_* |
| Subsequent time total | *P_+1_* | *P_+2_* |  | *P_+j_* | 1 |

Using the most general information on the matrix, we began quantifying the changes associated with LULC changes for each category using net change (in absolute value) given as:

$N_{j}=|P_{+j}-P_{i+}|$ (2)

Though such a net change in the quantity of a category indicates a definite change on the landscape, a lack of net change ($N_{j}$=0) does not necessarily indicate a lack of change on the landscape since the net change fails to capture the swapping component of change. Swamp is component of change when a given quantity (hectare) of a category loss at one location is accompanied by the same quantity (hectare) of the category gain at another location. To fill such shortfalls, therefore, we adopted a methodology introduced by (Pontius Jr et al., 2004) that accounts persistence (proportions at the diagonal) in examining landscape change. This method assumes persistence dominates most landscapes (as evidenced in many studies) and transitions need to be analyzed relative to it. With such relative quantification, we decomposed transitions to its various components to identify signals of systematic land change.

**Component of change analysis**

A more precise way of detecting LULC dynamics is to quantify landscape transition relative to persistence. Gross losses and gains, net change, and swap are important components of total change in the analysis of landscape dynamics considering signals of persistence on the landscape. To arrive at each component, we first calculated gross loss ($L_{i}$) and gross gain ($G_{j}$) as follows:

$L_{i}=\left| P_{i+}-P_{jj} \right|$and $G_{j}=\left| P_{+j}-P_{jj} \right|$ (3)

$L_{i}$ is proportion of landscape that experiences gross loss of category *i* during the study period and $G_{j}$ is proportion of landscape that experiences gross gain of category *j* during the study period.

The second important component, swamp ($S_{j}$), defines the swap location change that occurs when the landscape demonstrates simultaneous gross gain and gross loss (such as reforestation in some portion of the landscape and deforestation in other part). This component is constructed by pairing each grid cell that gains with grid cell that losses to create a pair of grid cells that swap for each category. When amount of gain is equal to the amount of loss ($N_{j}=0$), then the total change is all the swamp. Hence, the total change is twice the gain (or loss). If not, the amount of swamp ($S_{j}$) was calculated as:

$S_{j}=2*Min(L_{i},G_{j})$ (4)

$S_{j}$ does not capture portion of the change in excess of gain(loss) when gains are not equal to losses. The net change captures remaining unpaired gain or loss after all gains and losses have been paired to compute the amount of swap. Hence, $N_{j}$, defined in Eq (2) above can be redefined as:

$N_{j}=Max\left( L_{i},G_{j} \right)-Min\left( L_{i},G_{j} \right)=|P_{+j}-P_{i+}|$ (5)

Having the swamp and the net change components, we calculated total change for each category as the sum of the swamp change and net change as:

$T_{c}=S_{j}+N_{j}=Max\left( L_{i},G_{j} \right)+Min\left( L_{i},G_{j} \right)$ (6)

**Vulnerability to LULCC analysis**

Once we analyzed each component of change (transition), indices of persistence were calculated to show vulnerability of each category to LULC dynamics as follows:

$$G_{P}=G_{j}/P_{j}$$

$L_{P}=L_{j}/P_{j}$ (7)

$$N_{P}=N_{j}/P_{j}$$

Where $G_{P}$ is gross gain to persistence ratio, $L_{P}$ is gross loss to persistence ratio, $N_{P}$ is net change to persistence ratio and $P_{j}$ is proportion of the landscape of a category *j* that did not exhibit any change (transition).

Land use land cover categories with relatively higher $G_{P}$were asserted to have a higher tendency to gain surface instead of remaining stable, whereas land use land cover categories with relatively higher $L_{P}$ were declared to be highly vulnerable to loss surface (loss surface instead of remaining stable). $N_{P}$ shows magnitude of net surface loss/gain relative to persistence.

On the other hand, based on such relative (to persistence) quantifications, there may be misleading decisions showing lower tendencies to change of a LULC category while undertaking the greatest loss/gain and the highest total change. Such inconsistency in signaling change can be explained by size of LULC categories and their persistence levels. A larger transition observed in a category in the transition matrix may not necessary indicate relatively higher LULC change in the category as a category that account for large portions of the landscapes may show higher transition due its share, hence, not systematic transition.

Pontius et. al. (2004) noted even through a random process, largest LULC categories may experience sizable transitions (changes) among themselves. Such wrong signals of transition along with the spurious landscape dynamics due to larger persistence size relative to changes (Alo & Pontius Jr, 2008) leads to the adoption of identification of key and systematic patterns of change separate from any persistence level and land cover size (Briones & Varas, 2016).

**Detecting key signals of LULC change**

The analysis of persistence, gains and losses is instructive, but it fails to identify whether transitions were random or systematic as it fails to examine the dynamics among the off-diagonal entries. Hence, we followed Pontius et. al. (2004) methodology to analyze the off-diagonal entries to identify systematic transitions of land change for a given landscape’s degree of persistence. In identifying systematic transitions within the transition matrix, we constructed two expected transition matrices based on losses and gains that are expected to occur due to random process. In the expected transition matrix based on gross again, the gross gain of any one category is distributed based on the distribution of the other categories in the initial time (1986) as Eq (8) assuming that the total gross gain of each category is fixed:

$G_{ij}=\left( P_{+j}-P_{jj} \right)*\left( \frac{P_{i+}}{\sum_{i=1,i\neq j}^{J} P_{i+}} \right)$ (8)

Whereas, in the expected transition matrix based gross loss, the gross loss of any one category is distributed based on the distribution of the other categories in the subsequent time (2016) as Eq (9) assuming that the total gross loss of each category is fixed:

$L_{ij}=\left( P_{i+}-P_{jj} \right)*\left( \frac{P_{+j}}{\sum_{i=1,i\neq j}^{J} P_{+j}} \right)$ (9)

Where $G_{ij}$ is the expected transition from category *i* to category *j* due to a random process of gain, $L_{ij}$ is the expected transition from category *i* to category *j* due to a random process of loss, ($P_{+j}-P_{jj}$) is the observed gross gain of category *j*, ($P_{i+}-P_{jj}$) is the observed gross loss of category *i*, $P_{i+}$ is the size of category *i* in the initial time, $P_{+j}$ is the size of category *j* in the subsequent time, and the notation $i\neq j$ in the summation of each equations excludes size of the category under consideration from the sum of the sizes.

Eq (8) rearranges only the off-diagonal transitions within each column while holding persistence constant and Eq (9) rearranges only the off-diagonal transitions within each row while holding the persistence constant on the diagonal.

A transition is said to be systematic if the difference between transition observed in observed transition matrix deviates from transition expected in expected transition matrix. To provide conclusive evidence of key signal of change, category *i* should systematically loss to category j whilst category *j* is systematically gaining from category *i*. i.e., if the differences between observed and expected transitions due random losses and the differences between observed and expected transitions due random gains are significantly positive (Alo & Pontius Jr, 2008).
