# Supplementary material for: Multi-level determinants of land use land cover change in Tigray, Ethiopia: A mixed-effects approach using socioeconomic panel and satellite data
Source: PLoS One. 2024 Jun 13;19(6):e0304896. doi: 10.1371/journal.pone.0304896 (PMC11175475; doi:10.1371/journal.pone.0304896)
Supplement: S4 Appendix — (DOCX) [file pone.0304896.s009.docx]

**S4 Appendix. A household survey questionnaire.** This questionnaire was used to conduct a follow-up survey to the Ethiopia Rural Socioeconomic Survey to collect data on the beliefs and opinions of rural households regarding climate and land use land cover changes.

**ADDIS ABABA UNIVERSITY**

**COLLEGE OF DEVELOPMENT STUDIES**

**CENTER FOR ENVIRONMENT AND DEVELOMENT**

**A HOUSEHOLD SURVEY QUESTIONNAIRE**

**Introduction and consent**

Dear respondent!

Good morning/afternoon! My name is (YOUR NAME) and I am working as part of the data collection team of the PhD study entitled “***Socio-Ecological Vulnerability and Livelihood Resilience to Climate and Land Use Changes: Evidence from Farming Communities in Tigray Region.***” This is being conducted as a supplementary survey collecting primary data on climate and land use change perception and ecosystem services valuation which the secondary data of this study is lacking. You are selected for this survey because of your prior participation in the Ethiopian Rural Socioeconomic Survey (ESS) and your important role as a farming community member in the region. Your participation is based on your willingness to take part.

I will ask you a series of questions that would take about 25-30 minutes. Therefore, your kind cooperation with honest responses to the household survey questionnaire will be vital for the overall success of the study. It is purely for academic purpose (PhD study); results and any other details will not be used for un-intended purpose. To this end, anonymity and confidentiality of our respondents are highly guaranteed unless and otherwise under mutual agreement between the concerned parties. If you don’t choose to participate, it will in no way impact you in any aspect. Before we begin, do you want to ask me any questions about the survey? [***Listen questions and provide appropriate responses***].

Shall I continue in asking you each question?

If “Yes” Continue...

If “No” go to the next respondent.

**General instruction for the interviewer/enumerator**

- Please put the code chosen by the respondent in the space provided for some of the close-ended questions and check the chosen responses for those which do not have space to put the codes;
- The respondent can choose more than one response for some questions (multiple response item is possible);
- Write down the response for open-ended questions whenever the respondent provides and necessary;

**MODULE 1: HOUSEHOLD IDENTIFICATION**

| SN | Questions | Code Description | | |
| --- | --- | --- | --- | --- |
|  | Household identification No. | \|  \| \| --- \| | | |
|  | Zone | 1= North Western  2= Central  3= East  4= South [South and South Eastern]  5= West | | |
|  | Woreda | 11= Tahtay Adiyabo  12=Laelay Adiyabo  13=Medebay Zana  15=Asegede Tsimbila  16=Tselemti  21=Mereb Lehe  22=Ahiferom  23=Were Lehe  24=Adwa -rural  26=Tahtay Maychew | 27=Nader Adet  28=Kola Temben  29=Degua Temben  31=Gulo Meheda  33=Saesi Tsadamba  34=Ganta Afeshum  35=Hawuzen  36=Klite Awlalo  37=Atsbi Wenberta | 41=Seharti Samre  42= Adi Gudem  43=Hintalo Wajirat  44=Alaje  45=Endamehoni  46=Raya Azebo  48=Ofla  51=Kafta Humera  52=Welkayit  53=Tsegede |
|  | *Kebele* | \|  \| \| --- \| | | |
|  | Village/Kushet | \|  \| \| --- \| | | |
|  | Agro-ecology type | 1= Highland  2= Midland  3= Lowland | | |
|  | Respondent sex | 1= Male  2= Female | | |
|  | Date of interview | \|  \| \| --- \| | | |
|  | Enumerator name | \|  \| \| --- \| | | |

**MODULE 2: HOUSEHOLD DEMOGRAPHICS**

| **SN** | **Question(s)** | **Code Description** | |
| --- | --- | --- | --- |
|  | Sex of the household head | 1=Male  2=Female | |
|  | Age of the household head **[write in years]** | \|  \| \| --- \| | |
|  | Level of education of the household head | 1=Illiterate  2=Informal education (religious, adult education)  3= Primary school (1-8)  4= Secondary school (9-10)  5= Preparatory (11-12)  6= Above preparatory | |
|  | Years of schooling **[write in years]** | \|  \| \| --- \| | |
|  | Marital status of the household head  [**If age is above 18 years**] | 1= Never married  2= Married  3= Divorced  4= Separated  5= Widowed | |
|  | What is/was your house head's main industry of occupation?  **[Considering farming can also alternative occupation of the household]** | 1= Agriculture  2= Mining  3= Manufacturing  4= Electricity  5= Construction  6= Transportation  7= Buying and Selling any commodity  8= Financial Services  9= Personal Services/own business  10= Education  11= Health  12= Public Administration  13= Other (Specify) | |
|  | Religious affiliation of the household head | 1= Orthodox  2= Protestant  3= Catholic  4= Muslim  5= Other (specify) | |
|  | The house head responsibility in the community | 1= Member of the community  2= Religious leader  3= Development group leader  4= Kebele administrator  5= Council or cabinet member  6= Other (specify) | |
|  | Length of years in the current residence **[write in years]**  Probe as *[how long have you been living in this current residence?]* | \|  \| \| --- \| | |
|  | House head farming experience **[write in years]** | \|  \| \| --- \| | |
|  | Is the house head a model farmer?  Probe as [*Did you or any of your household member receive an award because of his/her successful farming practices by any organization?]* | 1= Yes  2=No | |
|  | Household size (**write in** number)  *[Please let the respondent to give the number of household members categorized by sex and age groups]* | No of male HH members ≤ 14 | \|  \| \| --- \| |
|  |  | No of female HH members ≤ 14 | \|  \| \| --- \| |
|  |  | No of male HH members [15-64] | \|  \| \| --- \| |
|  |  | No of female HH members [15-64] | \|  \| \| --- \| |
|  |  | No of male HH members ≥65 | \|  \| \| --- \| |
|  |  | No of female HH members ≥ 65 | \|  \| \| --- \| |

**MODULE 3: HOUSEHOLD PERCEPTION OF CLIMATE VARIABILITY AND CHANGE AND ITS INDUCED SHOCKS**

| **SN** | **Question(s)** | **Code Description** | | | | | | |
| --- | --- | --- | --- | --- | --- | --- | --- | --- |
|  | Have you heard of what climate change means from any source? | 1= Yes  2= No ………………………… **[Skip to M3.3]**  *[Message: M3.1 is the basis to decide whether a household perceives climate change or not. A “****No****” response to this question is very uncommon in situations where Gov’t and NGO effort to deal with climate change is visible. Hence, probe more and make sure this is the right perception of the respondent before you decide to skip]* | | | | | | |
|  | If yes to M3.1, on which of the following platforms, have you heard about climate variability and change?  **[Multiple choice is possible]** | 1= Radio  2= Television  3= Neighbours  4= Extension workers (health/agriculture)  5= Friends or colleagues  6= Family members/Relatives  7= Religious leaders  8=Community development work meeting  9= Other (Specify) | | | | | | |
|  | What climate change manifestations can you identify in your community during the last 10-20 years?  *[Probe as have you recognize … 1 to 9 or other … in your community during the last 10-20 years?]*  **[Multiple response is possible]** | 1= Increase in temperature  2= Decrease in rainfall amount  3= Frequent droughts  4= Frequent floods  5= Rising food prices  6= Increase of animal diseases  7=Increase of crop pest and/or crop failure  8= Other (Specify)  9= Don’t know/Don’t understand | | | | | | |
|  | How do you perceive the change in the temperature and rainfall in the last 30 years? | | | | | | | |
|  | **Change in temperature** | **1 = Increased** | **2 = No Change** | | **3 = Decreased** | | **4 = I Do Not Know** | |
|  | Overall temperature |  |  | |  | |  | |
|  | Minimum temperature |  |  | |  | |  | |
|  | Maximum temperature |  |  | |  | |  | |
|  | Dry season (‘*Bega’*) temperature |  |  | |  | |  | |
|  | Summer season (*‘Kirmet’*) temperature |  |  | |  | |  | |
|  | *‘Belg*’ season temperature |  |  | |  | |  | |
|  | Hot days in a year |  |  | |  | |  | |
|  | Cold days in a year |  |  | |  | |  | |
|  | Hot nights in a year |  |  | |  | |  | |
|  | Cold nights in a year |  |  | |  | |  | |
|  | **Change in the amount of rainfall** |  |  | |  | |  | |
|  | Overall rainfall amount |  |  | |  | |  | |
|  | Dry season (‘*Bega’*) rainfall amount |  |  | |  | |  | |
|  | Summer season (*‘Kirmet’*) rainfall amount |  |  | |  | |  | |
|  | ‘*Belge’* season rainfall amount |  |  | |  | |  | |
|  | Unusual timing of rains (earlier/later/erratic) |  |  | |  | |  | |
|  | Frequency of droughts |  |  | |  | |  | |
|  | Frequency of floods |  |  | |  | |  | |
|  | Decreased rainfall with unusual timing of rains |  |  | |  | |  | |
|  | What do you think are the main drivers of climate change?  **[Multiple response is possible]** | 1= Deforestation  2= Population growth  3= Agricultural expansion into forest/grass/wet land  4= Rapid urbanization and changes in life style  5= Industrial expansion into agricultural forest/grass/wet land  6= Other (Specify) | | | | | | |
|  | To what extent do you believe that climate change is already affecting your household? | 1= Not at all  2= A bit  3= Somewhat affected  4= Quite a lot  5= Highly affected  6= I do not know | | | | | | |
|  | Would you please indicate the type of shocks that climate change has brought to you or your household during in the last 30 years? | | | | | | | |
|  | **Shock type** | **Occurrence**  **1= Yes**  **2=No** | **Shock frequency** | | | **How do you rate its frequency? *** | | **Shock severity^#^** |
|  | Crop failure |  |  | | |  | |  |
|  | Shortage of water for irrigation |  |  | | |  | |  |
|  | Shortage of water for home/animal consumption |  |  | | |  | |  |
|  | Emergence/resurgence of new pests (weeds) and insects |  |  | | |  | |  |
|  | Increased frequency of droughts |  |  | | |  | |  |
|  | Increased frequency of floods |  |  | | |  | |  |
|  | Geographic isolation |  |  | | |  | |  |
|  | Livestock disease |  |  | | |  | |  |
|  | Crop pests and diseases |  |  | | |  | |  |
|  | Conflict over diminishing resources |  |  | | |  | |  |
|  | Food price inflation |  |  | | |  | |  |
|  | Code*:1= Highly decreased 2= Decreased 3= No change 4= Increased 5= Highly increased  Code^#^: 1= High 2= Medium 3= Low | | | | | | | |
|  | If yes for M3.7.1, what were the main reason for the crop failure?  **[Multiple response is possible]** | 0= No crop failure  1= Erratic rainfall  2= Lack of improved seeds  3= Unaffordable price of inputs  4= Low level of soil fertility  5= Crop pest and disease  6= Shortage of farm oxen  7= Other (Specify) | | | | | | |
|  | Are there any adaptation strategies you have made for the change in climate? | 1= Yes  2= No ………………………… **[Skip to M4.1]**  *[Message: Adaptation strategies are techniques farmers follow to come out of shocks they experience. It may be an indigenous knowledge or acquired from extension services. As a result, farming households are accustomed to adapt at least a strategy to overcome shocks and following* ***No strategy*** *is assumed to be* ***Naïve*** *in farming community. Hence, probe more and make sure* ***No strategy*** *was followed before you decide to skip]* | | | | | | |
|  | If yes for M3.9, what adaptation strategies have you so far employed in response to long term change in precipitation and temperature over the last 30 years? | | | | | | | |
|  | **Strategies** | | | **Response [1= Yes 2=No]** | | | | |
|  | Shift to drought resistant crop varieties | | |  | | | | |
|  | Practicing crop diversification (mixed crops) | | |  | | | | |
|  | Different/new planting dates | | |  | | | | |
|  | Shift to show growing or maturing crops | | |  | | | | |
|  | Move to different sites (migration) | | |  | | | | |
|  | Change from crops to livestock | | |  | | | | |
|  | Change from livestock to crops | | |  | | | | |
|  | Increasing water conservation/harvesting on farms | | |  | | | | |
|  | Use of irrigation (home gardens) | | |  | | | | |
|  | Use of chemicals, fertilizers, manure and pesticides | | |  | | | | |
|  | Shading and sheltering young plants | | |  | | | | |
|  | Mixing crops and livestock (diversification) | | |  | | | | |
|  | Adjusting livestock management practices | | |  | | | | |
|  | Use of micro-insurance schemes | | |  | | | | |
|  | Changed agriculture to non-farm activities | | |  | | | | |
|  | Participate in soil and water conservation practices | | |  | | | | |
|  | Use of prayer and socio-cultural adaption schemes | | |  | | | | |

**MODULE 4: HOUSHOLD PERCEPTION OF LAND USE CHANGE**

| **SN** | **Question(s)** | **Code Description** | | | | | | | |
| --- | --- | --- | --- | --- | --- | --- | --- | --- | --- |
|  | What land use types [ecosystems] do you have in your community [nearby the locality]?  **[Multiple choice is possible]** | 1= Farmland  2= Grassland  3= Plantation forestland  4= Natural forestland  5= Wetland  6= Other (Specify) | | | | | | | |
|  | Over the past 10-20 years, have you noticed any change in land use types in your community/areas close to your community?  Probe as *[Did you recognize any improvements or decline in the land coverage any of the land use types during your stay in these community/areas close to your community?]* | 1= Yes  2= No ………………………… **[Skip to M4.10]**  *[Message: You are about to skip almost the whole module 4. Please probe more as “you mean you did not recognize any change in area coverage of forest, grasslands, water bodies and farmlands?” before you decide to skip]* | | | | | | | |
|  | If yes to M4.2, select land use that has significantly changed over 30 years?  **[Multiple choice is possible]** | 1= Farmland  2= Grassland  3= Plantation forestland  4= Natural forestland  5= Wetland  6= Other (Specify) | | | | | | | |
|  | Could you please tell the direction of change in the area coverage of the following land uses in your community/areas close to your community over the last 10-20 years? | | | | | | | | |
|  | **Land use** | **Perceived change before 10 years**  **[Before 2000 EC]** | | | | | | | |
|  | Farmland |  | | | | | 1= Deceased  2= No change  3= Increased  4= I do not know | | |
|  | Grassland |  | | | | |  |  |  |
|  | Plantation forestland |  | | | | |  |  |  |
|  | Natural forestland |  | | | | |  |  |  |
|  | Wetland |  | | | | |  |  |  |
|  | Other (Specify) |  | | | | |  |  |  |
|  | **Land use** | **Perceived change with in the last 10 years**  **[2000-2011 EC]** | | | | | | | |
|  | Farmland |  | | | | | 1= Deceased  2= No change  3= Increased  4= I do not know | | |
|  | Grassland |  | | | | |  |  |  |
|  | Plantation forestland |  | | | | |  |  |  |
|  | Natural forestland |  | | | | |  |  |  |
|  | Wetland |  | | | | |  |  |  |
|  | Other (Specify) |  | | | | |  |  |  |
|  | At what scale do you think is the change in land use happening?  **[Multiple response is possible]** | 1= District (Woreda) level  2= Community (Kebele) level  3= Watershed/ecosystem level  4= Landscape level | | | | | | | |
|  | What do you think are the main causes of land use change in your locality/community, in general?  **[Multiple response is possible]** | 1= Climate change  2= Population growth  3= Urbanization  4= Infrastructure development  5= Anthropogenic factors (overgrazing, sale of firewood and charcoal extraction)  6= Inappropriate government policies (settlement policies)  7= Inappropriate development interventions (appropriation of investment land)  8= Other (Specify) | | | | | | | |
|  | Please specify, by land use type, the major direct drivers of the change in the major land use types (ecosystems) in your community or nearby your locality?  Probe as *[Can … [driver] be taken as the major cause for change in … [land use type] in your community?]* | | | | | | | | |
|  | **Drivers** | **Farm land**  **1=Yes**  **2=No** | **Grass land**  **1=Yes**  **2=No** | **Plantation forestland**  **1=Yes**  **2=No** | **Natural forestland**  **1=Yes**  **2=No** | | | **Wet land**  **1=Yes**  **2=No** | **Other**  **1=Yes**  **2=No** |
|  | Conversion to industrial area |  |  |  |  | | |  |  |
|  | Conversion to urban area |  |  |  |  | | |  |  |
|  | Conversion to tourist area |  |  |  |  | | |  |  |
|  | Conversion to dams |  |  |  |  | | |  |  |
|  | Conversion to infrastructures (road, school, health etc.) |  |  |  |  | | |  |  |
|  | Proximity to road |  |  |  |  | | |  |  |
|  | Increased fuel demand |  |  |  |  | | |  |  |
|  | Increased food demand |  |  |  |  | | |  |  |
|  | Increased production of agricultural export commodities |  |  |  |  | | |  |  |
|  | Increased production of cash crops |  |  |  |  | | |  |  |
|  | Other (Specify) |  |  |  |  | | |  |  |
|  | What impacts of land use change have you noticed over the last 30 years?  Probe as *[In your view, would an increase in … [land use] result in Less/No/ More change on … [the impacted]?]* | | | | | | | | |
|  | **Impacts on** | **Level of change in land use type** | | | | | | | |
|  |  | **Farm land**  **1=Yes, More**  **2=Yes, less**  **3=No** | **Grass land**  **1=Yes, More**  **2=Yes, less**  **3=No** | **Plantation forestland**  **1=Yes, More**  **2=Yes, less**  **3=No** | **Natural forestland**  **1=Yes, More**  **2=Yes, less**  **3=No** | | | **Wet land**  **1=Yes, More**  **2=Yes, less**  **3=No** | **Other**  **1=Yes, More**  **2=Yes, less**  **3=No** |
|  | Land holding |  |  |  |  | | |  |  |
|  | Soil fertility |  |  |  |  | | |  |  |
|  | Land productivity (crop and livestock) |  |  |  |  | | |  |  |
|  | Livestock holding |  |  |  |  | | |  |  |
|  | Livestock composition |  |  |  |  | | |  |  |
|  | Rangeland |  |  |  |  | | |  |  |
|  | Biodiversity loss (like indigenous trees) |  |  |  |  | | |  |  |
|  | Conflict on rangeland resources |  |  |  |  | | |  |  |
|  | Migration (agricultural labor insufficiency) |  |  |  |  | | |  |  |
|  | Livelihood income diversification |  |  |  |  | | |  |  |
|  | Income and employment created |  |  |  |  | | |  |  |
|  | Benefits from developed infrastructure |  |  |  |  | | |  |  |
|  | Subsidies/payments |  |  |  |  | | |  |  |
|  | Thinking about the past and future (in 5-10 years’ time), overall, do you believe that the impacts of these land uses will be positive or negative? | | | | | | | | |
|  | **Land use (past)** | **Perceived impact with in the past [Before 2011 EC]** | | | | | | | |
|  | Farmland |  | | | | | 1= Negative  2= Neutral  3= Positive  4= I do not know | | |
|  | Grassland |  | | | | |  |  |  |
|  | Plantation forestland |  | | | | |  |  |  |
|  | Natural forestland |  | | | | |  |  |  |
|  | Wetland |  | | | | |  |  |  |
|  | Other (Specify) |  | | | | |  |  |  |
|  | **Land use (future)** | **Perceived impact with in the future [After 5-10 years]** | | | | | | | |
|  | Farmland |  | | | | | 1= Negative  2= Neutral  3= Positive  4= I do not know | | |
|  | Grassland |  | | | | |  |  |  |
|  | Plantation forestland |  | | | | |  |  |  |
|  | Natural forestland |  | | | | |  |  |  |
|  | Wetland |  | | | | |  |  |  |
|  | Other (Specify) |  | | | | |  |  |  |
|  | Thinking about a good mix of land uses for your community, how important are the following outcomes to you? | | | | | | | | |
|  | **Statement** | | | **Response** | | 1= Not important  2= Moderately important  3= Important  4= Highly important  5= I do not know | | | |
|  | The environment is free of harmful chemicals | | |  | |  |  |  |  |
|  | The number of people living in the community is increasing | | |  | |  |  |  |  |
|  | Water is available for all uses | | |  | |  |  |  |  |
|  | Employment opportunities are growing | | |  | |  |  |  |  |
|  | Infrastructures are safe and in good condition | | |  | |  |  |  |  |
|  | Soils in your community are protected from damage | | |  | |  |  |  |  |
|  | Business is prosperous for shops and traders in the community | | |  | |  |  |  |  |
|  | Everyone in the community benefits from land uses, not just some people | | |  | |  |  |  |  |
|  | Indigenous trees are protected from damage | | |  | |  |  |  |  |

**MODULE 5: HOUSEHOLD PERCEPTION OF ECOSYTEM SERVICES**

| **SN** | **Question(s)** |  | | | **Code Description** | | | |
| --- | --- | --- | --- | --- | --- | --- | --- | --- |
|  | What are the major ecosystem services provided by or you get from your ecosystems (land use types) currently?  Probe as [*Ecosystem services refers to the benefits ecosystems/nature gives to the society such as farming and forest products, clean water and air, wildlife habitat, stabilized climate and renewed soils, and human recreation. What major ecosystem services you gain from your ecosystems?*]. | | | | | | | |
|  | **Ecosystem service** | | **Farm land** | **Grass land** | **Plantation forestland** | | **Natural forestland** | **Wet land** |
|  |  |  | 1= Yes 2= No | 1= Yes 2= No | 1= Yes 2= No | | 1= Yes 2= No | 1= Yes 2= No |
|  | Farming food production (crop, meat, dairy, fish, honey) | |  |  |  | |  |  |
|  | Forest production (timber, fuel wood, forage, grass etc.) | |  |  |  | |  |  |
|  | Clean water (surface and underground water for drinking and non-drinking) | |  |  |  | |  |  |
|  | Genetic materials or resources | |  |  |  | |  |  |
|  | Medicinal and cosmetic plants | |  |  |  | |  |  |
|  | Water purification (Natural filtration through nutrient uptake; retention of  particles and pollutants) | |  |  |  | |  |  |
|  | Erosion control | |  |  |  | |  |  |
|  | Flood protection | |  |  |  | |  |  |
|  | Clean air (air quality regulation) | |  |  |  | |  |  |
|  | Maintenance of soil fertility | |  |  |  | |  |  |
|  | Climate regulation | |  |  |  | |  |  |
|  | Pest and disease control | |  |  |  | |  |  |
|  | Pollination | |  |  |  | |  |  |
|  | Biodiversity maintenance | |  |  |  | |  |  |
|  | Nature recreation and tourism | |  |  |  | |  |  |
|  | Spiritual/cultural/aesthetic values | |  |  |  | |  |  |
|  | Wild life habitat | |  |  |  | |  |  |
|  | *Code: 1= Not Important at all 2= Rather not important 3=Rather important 4=Important 5=Very Important, 6= I do not know | | | | | | | |
|  | In your opinion; what has been the overall change [during the last 10-20 years] in the state of ecosystem services you get from land type in your community? | | | | | | | |
|  | **Ecosystem from** | | **State of the ecosystem** | | | 1= Strong deterioration  2= Deterioration  3= No significant change  4= Improvement  5= Major improvement | | |
|  | Farmland | |  | | |  |  |  |
|  | Grassland | |  | | |  |  |  |
|  | Plantation forestland | |  | | |  |  |  |
|  | Natural forestland | |  | | |  |  |  |
|  | Wetland | |  | | |  |  |  |
|  | Other (Specify) | |  | | |  |  |  |
|  | What major environmental problems do you think that you or your community have been facing as a result of deteriorating state ecosystem services?  **[Multiple response is possible]** | | 1= Climate change  2= Land degradation/erosion  3= Deforestation  4= Water pollution  5= Waste management problems  6= Other (Specify) | | | | | |
|  | Please provide your agreement to the following statement.  *“We need to conserve ecosystems (land types) that provides ecosystem services in our community.”* | | 1= Strongly Disagree  2= Somewhat Disagree  3= Neither Agree nor Disagree  4= Somewhat agree  5= Strongly agree | | | | | |
|  | Do you regularly participate in ecosystem conservation activities [like soil and water conservation, afforestation/reforestation, forest protection and guarding etc.] in your community? | | 1= Yes  2= No | | | | | |

**MODULE 6: HOUSEHOLD WILLINGNESS TO PAY (WTP) FOR ECOSYTEMS SERVICES**

| **SN** | **Question(s)** | | **Code Description** | | | | | |
| --- | --- | --- | --- | --- | --- | --- | --- | --- |
|  | ***Imagine the following fictional scenario****: Assume that your ecosystem has faced a change. All ecosystems (land types) your community have now are to be changed into settlement (buildings and other structures) with no chance to get ecosystem services that your community had been served. If you do not have the chance to live on other occupation(s) but as a farming household, how much would you be willing to pay to get each ecosystem service assuming similar ecosystem services are available in other communities near by your community?* | | | | | | | |
|  | **Ecosystem service**  **[Applicable for ecosystem services selected in any of the land types in M5.1]** | **Willing to pay ETB 30 per month***  **1= Yes**  **2=No** | **If no to ETB 30/month, choose pay1 [20, 10, 5, 2, 1, 0]** | | **If chosen pay1 is [20, 10], ask what if pay2 is [25, 15]. If agreed take pay2, if no to pay2 take pay1** | **If chosen pay1 is [5, 2, 1, 0], take pay1** | **If yes to ETB 30/month, choose pay3 [60, 90, 120]** | **If even 60 is not selected, negotiate reducing pay3 by 10. Whereas if pay3 is [60, 90, 120], take it.** |
|  | Farming food production (crop, meat, dairy, fish, honey) |  |  | |  |  |  |  |
|  | Forest production (timber, fuel wood, forage, grass etc.) |  |  | |  |  |  |  |
|  | Clean water (surface and underground water for drinking and non-drinking) |  |  | |  |  |  |  |
|  | Genetic materials/resources |  |  | |  |  |  |  |
|  | Medicinal and cosmetic plants |  |  | |  |  |  |  |
|  | Water purification (Natural filtration through nutrient uptake; retention of  particles and pollutants) |  |  | |  |  |  |  |
|  | Erosion control |  |  | |  |  |  |  |
|  | Flood protection |  |  | |  |  |  |  |
|  | Clean air (air quality regulation) |  |  | |  |  |  |  |
|  | Maintenance of soil fertility |  |  | |  |  |  |  |
|  | Climate regulation |  |  | |  |  |  |  |
|  | Pest and disease control |  |  | |  |  |  |  |
|  | Pollination |  |  | |  |  |  |  |
|  | Biodiversity maintenance |  |  | |  |  |  |  |
|  | Nature recreation and tourism |  |  | |  |  |  |  |
|  | Spiritual/cultural/aesthetic values |  |  | |  |  |  |  |
|  | Wild life habitat |  |  | |  |  |  |  |
|  | *The starting point of monthly payment [ETB ___ in this hypothetical case] will be reformed [selected] by searching for average reasonable amount for ecosystem services through focus group discussion (FGD) to be conducted with farming community members. | | | | | | | |
|  | What main reason do you think that can justify your positive willingness to pay?  **[If positive WTP is reported]** | | | 1= Maximum I can afford  2= Amount I can easily affordable  3= Enough if everyone paid  4= Normally give this amount to similar issues  5= Random choice | | | | |
