# Supplementary material for: Multi-level determinants of land use land cover change in Tigray, Ethiopia: A mixed-effects approach using socioeconomic panel and satellite data
Source: PLoS One. 2024 Jun 13;19(6):e0304896. doi: 10.1371/journal.pone.0304896 (PMC11175475; doi:10.1371/journal.pone.0304896)

PLOS ONE

Multi-level determinants of Land Use Land Cover Change in Tigray - A Mixed-Effects Approach Using Socioeconomic Panel and Satellite Data

--Manuscript Draft--

**Manuscript Number:**	PONE-D-23-33020
**Article Type:**	Research Article
**Full Title:**	Multi-level determinants of Land Use Land Cover Change in Tigray - A Mixed-Effects Approach Using Socioeconomic Panel and Satellite Data
**Short Title:**	Land Use Land Cover Change and its drivers
**Corresponding Author:**	Tadele Tafese Habtie, MSc<br>Wolaita Sodo University<br>Addis Ababa, ETHIOPIA
**Keywords:**	land use choice, land cover change, livelihood zone, mixed-effects model, Tigray, Ethiopia
**Abstract:**	This study examined land use land cover change and its determinants in Tigray and its livelihood zones. We used socioeconomic panel, and satellite data, and applied a mixed-effects model to analyse the factors influencing land allocation among different uses, and transition matrix to analyse land cover dynamics. The results revealed that; land use choices were influenced by plot level factors (such as plot elevation, distance, soil type and quality, and land tenure), household characteristics (such as education, dependency ratio, plot size and number owned, income, livestock and asset, perception of climate change, and access to market and main road), and community level factors (precipitation, product price, population density and livelihood zone variations). Transition matrix analysis showed that between 1986 and 2016, 12.8% of forest was converted to bare land, 6.26% bare land was converted to pasture, and 5.84% of cropland was converted to forest. However, net deforestation occurred in most of the livelihood zones. Therefore, local communities faced environmental and socio-economic challenges from capital constraints induced land fallowing, land fragmentation, and unmanaged land cover change. The study recommended sustainable land use planning and management, market linkages, improved access to roads, forestry subsidies, land tenure security, and land consolidation programs.
**Order of Authors:**	Tadele Tafese Habtie, MSc
	Ermias Teferi, PhD.
	Fantu Guta, PhD.
**Additional Information:**	
**Question**	**Response**
**Financial Disclosure**<br><br>Enter a financial disclosure statement that describes the sources of funding for the work included in this submission. Review the submission guidelines for detailed requirements. View published research articles from *PLOS ONE* for specific examples.<br><br>This statement is required for submission and **will appear in the published article** if the submission is accepted. Please make sure it is accurate.	The author(s) received no specific funding for this work.

Powered by Editorial Manager® and ProduXion Manager® from Aries Systems Corporation

**Funded studies**<br>Enter a statement with the following details:<br>• Initials of the authors who received each award<br>• Grant numbers awarded to each author<br>• The full name of each funder<br>• URL of each funder website<br>• Did the sponsors or funders play any role in the study design, data collection and analysis, decision to publish, or preparation of the manuscript?<br><br>Did you receive funding for this work?	
**Competing Interests**<br><br>Use the instructions below to enter a competing interest statement for this submission. On behalf of all authors, disclose any competing interests that could be perceived to bias this work—acknowledging all financial support and any other relevant financial or non-financial competing interests.<br><br>This statement is required for submission and **will appear in the published article** if the submission is accepted. Please make sure it is accurate and that any funding sources listed in your Funding Information later in the submission form are also declared in your Financial Disclosure statement.<br><br>View published research articles from *PLOS ONE* for specific examples.	The authors have declared that no competing interests exist.

**NO authors have competing interests**<br><br>Enter: *The authors have declared that no competing interests exist.*<br><br>**Authors with competing interests**<br><br>Enter competing interest details beginning with this statement:<br><br>*I have read the journal's policy and the authors of this manuscript have the following competing interests: [insert competing interests here]*<br><br>* typeset	
**Ethics Statement**<br><br>Enter an ethics statement for this submission. This statement is required if the study involved:<br><br>• Human participants<br>• Human specimens or tissue<br>• Vertebrate animals or cephalopods<br>• Vertebrate embryos or tissues<br>• Field research<br><br>Write "N/A" if the submission does not require an ethics statement.<br><br>General guidance is provided below. Consult the submission guidelines for detailed instructions. **Make sure that all information entered here is included in the Methods section of the manuscript.**	NA

Powered by Editorial Manager® and ProduXion Manager® from Aries Systems Corporation

**Format for specific study types**<br><br>**Human Subject Research (involving human participants and/or tissue)**<br>• Give the name of the institutional review board or ethics committee that approved the study<br>• Include the approval number and/or a statement indicating approval of this research<br>• Indicate the form of consent obtained (written/oral) or the reason that consent was not obtained (e.g. the data were analyzed anonymously)<br><br>**Animal Research (involving vertebrate animals, embryos or tissues)**<br>• Provide the name of the Institutional Animal Care and Use Committee (IACUC) or other relevant ethics board that reviewed the study protocol, and indicate whether they approved this research or granted a formal waiver of ethical approval<br>• Include an approval number if one was obtained<br>• If the study involved *non-human primates*, add *additional details* about animal welfare and steps taken to ameliorate suffering<br>• If anesthesia, euthanasia, or any kind of animal sacrifice is part of the study, include briefly which substances and/or methods were applied<br><br>**Field Research**<br><br>Include the following details if this study involves the collection of plant, animal, or other materials from a natural setting:<br>• Field permit number<br>• Name of the institution or relevant body that granted permission	
**Data Availability**<br><br>Authors are required to make all data underlying the findings described fully available, without restriction, and from the time of publication. PLOS allows rare exceptions to address legal and ethical concerns. See the PLOS Data Policy and FAQ for detailed information.	No - some restrictions will apply

A Data Availability Statement describing where the data can be found is required at submission. Your answers to this question constitute the Data Availability Statement and **will be published in the article**, if accepted.<br><br>**Important:** Stating 'data available on request from the author' is not sufficient. If your data are only available upon request, select 'No' for the first question and explain your exceptional situation in the text box.<br><br>Do the authors confirm that all data underlying the findings described in their manuscript are fully available without restriction?	
**Describe where the data may be found in full sentences. If you are copying our sample text, replace any instances of XXX with the appropriate details.**<br><br>• If the data are **held or will be held in a public repository**, include URLs, accession numbers or DOIs. If this information will only be available after acceptance, indicate this by ticking the box below. For example: *All XXX files are available from the XXX database (accession number(s) XXX, XXX.).*<br>• If the data are all contained **within the manuscript and/or Supporting Information files**, enter the following: *All relevant data are within the manuscript and its Supporting Information files.*<br>• If neither of these applies but you are able to provide **details of access elsewhere**, with or without limitations, please do so. For example:<br><br>*Data cannot be shared publicly because of [XXX]. Data are available from the XXX Institutional Data Access / Ethics Committee (contact via XXX) for researchers who meet the criteria for access to confidential data.*<br><br>*The data underlying the results presented in the study are available from (include the name of the third party*	Data cannot be shared publicly because of data source access policy. Data are available from the WB Institutional Data Access for researchers who meet the criteria for access to confidential data.

and contact information or URL).<br>• This text is appropriate if the data are owned by a third party and authors do not have permission to share the data.<br><br>* typeset	
Additional data availability information:	Tick here if your circumstances are not covered by the questions above and you need the journal's help to make your data available.

Powered by Editorial Manager® and ProduXion Manager® from Aries Systems Corporation

Manuscript
Click here to access/download;Manuscript;Tadele_Tafese_Paper_2_Final_P

Multi-level determinants of Land Use Land Cover Change in Tigray - A Mixed-Effects Approach Using Socioeconomic Panel and Satellite Data

Tadele Tafese Habtie[a,*], Ermias Teferi[a], and Fantu Guta[b]

[a]*Centre for Environment and Development Studies, Addis Ababa University, Addis Ababa, Ethiopia*

[b]*Department of Economics, Addis Ababa University, Addis Ababa, Ethiopia*

[*]Corresponding author. Email: tadeleth74@gmail.com; Tel.: +251914766418

0009-0002-0256-0610

Abstract

This study examined land use land cover change and its determinants in Tigray and its livelihood zones. We used socioeconomic panel, and satellite data, and applied a mixed-effects model to analyse the factors influencing land allocation among different uses, and transition matrix to analyse land cover dynamics. The results revealed that; land use choices were influenced by plot level factors (such as plot elevation, distance, soil type and quality, and land tenure), household characteristics (such as education, dependency ratio, plot size and number owned, income, livestock and asset, perception of climate change, and access to market and main road), and community level factors (precipitation, product price, population density and livelihood zone variations). Transition matrix analysis showed that between 1986 and 2016, 12.8% of forest was converted to bare land, 6.26% bare land was converted to pasture, and 5.84% of cropland was converted to forest. However, net deforestation occurred in most of the livelihood zones. Therefore, local communities faced environmental and socio-economic challenges from capital constraints induced land fallowing, land fragmentation, and unmanaged land cover change. The study recommended sustainable land use planning and management, market linkages, improved access to roads, forestry subsidies, land tenure security, and land consolidation programs.

Keywords: land use choice, land cover change, livelihood zone, mixed-effects model, Tigray, Ethiopia

Introduction

Land is a crucial resource that supports human existence and development (Yesuph & Dagnew, 2019). However, human activities have caused changes to the land system that impact climate, ecosystem services, and land degradation (Newbold et al., 2015; Turner et al., 1995; van Schrojenstein Lantman et al., 2011; Verburg et al., 2015). Unmanaged land cover transitions increase the vulnerability of human-environment systems and threaten human well-being (Farley, 2008; Turner et al., 2007). Sustainable development promotes environment-neutral economic interests to manage forests, halt and reverse land degradation and biodiversity losses, and promote sustainable use of terrestrial ecosystems (Nkonya et al., 2016).

However, in Ethiopia, continuous land use changes have resulted in deforestation and land degradation mainly due to agricultural expansion, overgrazing, deforestation, and settlement development (Alemu et al., 2015a; Regasa et al., 2021). This has led to changes in land cover, which are aggravated by the ever-growing population and increased demands for ecosystem services (Temesgen et al., 2014). One of the most affected regions in Ethiopia is Tigray in the north, which is highly degraded and suffers from low agricultural productivity, food insecurity and poverty (Hachoofwe, 2012; Hurni et al., 2010; Van der Veen & Gebrehiwot, 2011).

Most previous studies on LULC change in Ethiopia have focused on change detection and/or macro-level determinant analysis (Tadesse et al., 2017; WoldeYohannes et al., 2018). However, few studies have considered both macro-level and household-level drivers of LULC change (Gedefaw et al., 2020; Miheretu & Yimer, 2018). Moreover, most studies have used cross-sectional data and aggregated analysis units, which may not capture the spatial and temporal heterogeneity of LULC dynamics (Abere

et al., 2020; Ewunetu et al., 2021). Therefore, there is a need for a more comprehensive and detailed analysis of LULC change and its drivers in Tigray.

To address this research gap, this study aims to examine determinants of land use choices and land cover dynamics in Tigray using socioeconomic panel and satellite images. The novel contributions of this study include: (1) We use a hierarchical model that examines how factors at different levels (plot, household, and community and above) affect land use choice, (2) We analyze land use changes at the plot level within households, (3) We use panel data with three waves to track how land use choices change over time, and (4) We disaggregate our analysis by livelihood zone (LHZ), which is a smaller and more homogeneous unit with regard to livelihoods than basin or sub-basin. This paper is organized as follows: Section 2 describes the materials and methods used in this study. Section 3 depicts the results. Section 4 presents the discussion. Section 5 concludes and provides policy implications.

Background

Tigray is a northern Ethiopian region with a population of about 6.1 million, mostly Orthodox Christian Tigrayans (95.6%). It covers an area of roughly 53,000 km² and has diverse landscapes, climates, and livelihoods. It has three main agro-climatic zones (lowland, midland, and highland) and 17 livelihood zones, with elevations ranging from 500 to 3200 meters above sea level (Figure 1). The average annual temperature in Tigray varies from 13-28°C, while the average annual rainfall is between 388-602 mm. (Aredehey et al., 2020).

Figure 1. Map of the study area showing the location, international and administrative boundaries, livelihood zones, and enumeration areas (EAs) of Tigray region in Ethiopia. Source: Author’s own work using ArcGIS software and spatial data on livelihood zones from FEWS NET (2018) and on EA from CSA Ethiopia (2019).

The region has a long and rich history of political and economic importance in Ethiopia since ancient times. It was the heart of the Aksumite Empire and has witnessed many wars in Ethiopia, including colonial wars, civil wars, and the Eritrean-Ethiopian War (Abbink, 2011). Currently, Tigray is facing one of the worst wars in the world after a conflict broke out between the federal and regional governments on November 4th, 2020.

Agriculture is the mainstay of Tigray's economy, accounting for about 36.7% of the regional GDP and employing about 83% of the workforce. It is based on a mixed farming system that combines crop and livestock production on the same farm, with 81.4% of farms engaged in crop production and 14.5% in livestock production (BoPF, 2018). However, the region is challenged by environmental degradation, climate change, food insecurity, and poverty. Deforestation and land degradation have been affecting the region for decades, causing severe soil erosion and yield reduction (Hachoofwe, 2012; Tesfaye et al., 2014; Yaynemsa, 2022).

As a result, Tigray is regarded as one of the most drought-prone and food-insecure areas in Ethiopia with frequent droughts occurring every two to four years (Maxwell et al., 2013; Mesele et al., 2019; Weldearegay & Tedla, 2018). To address these challenges the government has implemented various policies and programs such as the Productive Safety Net Program (PSNP), Soil and Water Conservation initiatives and community-based natural resource management approaches (including exclosures and cropland afforestation). These programs have achieved significant success stories that have been recognized internationally (Gebreegziabher et al., 2020; Gebremedhin et al., 2003; Mekuria et al., 2007).

Materials and methods

Data and data source

Socioeconomic data

The study used socioeconomic unbalanced panel data from 996 households and 2,211 plots across 17 LHZs in Tigray. It came from the World Bank's Living Standards Measurement Study, Integrated Survey on Agriculture (LSMS-ISA). The LSMS-ISA is a representative survey at both national and regional levels, and it conducted the Ethiopia Rural Socioeconomic Survey (ERSS) in three waves (2012, 2014, and 2016) from a representative sample of households in rural areas, small towns, and medium and large towns. The LSMS-ISA survey provides detailed information on plot, household, and community level variables. It is a valuable source of data for researchers studying socioeconomic conditions in Ethiopia. In Tigray, the LSMS-ISA surveyed 387 households repeatedly in 37 EAs in all three waves in rural areas and small towns, where the population is expected to predominantly live on agriculture. Additionally, the study re-surveyed the 387 households on their public climate and LULC change beliefs and opinions to complement the LSMS-ISA data.

Satellite image processing and classification

On-demand Landsat images for 1986 and 2016 from Landsat 5 Thematic Mapper, and Landsat 8 Operational Land Imager, respectively, were used for land cover classification (Table 1). We selected 1986 as a drought year and 2016 as the last year of the panel data. All Landsat images were accessed free of charge from the US Geological Survey Center for Earth Resources Observation and Science via Earth Explorer. Essential pre-processing operations such as radiometric and atmospheric corrections were performed by the data providers.

Table 1. Summary of LULC categories and descriptions

LULC category	Description
Forest	Various types of forests, woodlands, shrublands, and agroforestry
Pasture	Grasslands in different moisture and terrain conditions
Cropland	Areas with crop cultivation, both small and large scale
Bare land	Degraded or exposed land, mining and construction sites, river courses
Others uses	Settlements, water bodies, and unidentified LULC

We also used ancillary data to improve classification accuracy and support interpretation of land cover changes. LHZ data were obtained from Famine Early Warning Systems Network. Digital Elevation Model (SRTM-30m) and its derived datasets such as slope were used to supplement satellite data in mapping land cover, as vegetation classes are often limited to specific agro-climatic conditions. DEM of SRTMGL1 (NASA Shuttle Radar Topography Mission Global 1 arc second (~30 m) V003) was obtained from http://e4ftl01.cr.usgs.gov/SRTM/SRTMGL1.003/. Old maps and Topographic maps of Tigray were used to assist image classification of the 1986 satellite images.

A hybrid supervised/unsupervised classification approach was implemented together with successive Geographic Information System operations (spatial analysis) to classify the Landsat imageries based on homogenous land units. First, to determine the spectral classes Iterative Self-Organizing Data Analysis clustering was performed. Second, ground truth data was collected from field observations as well as Google Earth to associate the spectral classes with the cover types of the already defined classification scheme. Key Informant interviews and old topo-sheets were used to obtain information about the historical land cover status of every sample points. Finally, images were classified using Maximum Likelihood Algorithm based on the training samples developed in the previous step. In order to use the classified maps for further LULC analysis, the classification accuracy was assessed. Overall accuracies of 87% and 89% were attained for the 1986 and 2016 classified maps, respectively.

Method of data analysis

We want to know if and why unmanaged LULC changes and how it affects rain-fed farming. We use land use choice analysis and a multi-method land cover change detection procedure. The following sub-sections presented theoretical and empirical models for the land use choice analysis and methods of land cover change detection adopted.

Theoretical model

Literature on the spatial land allocation theory (Lee, 1996), postulates that land is divided between alternative uses according to the highest land rent obtainable, given the land's special characteristics with regards to location and physical conditions. However, in Ethiopia, land is publicly owned and hence land markets do not exist effectively and land prices reflecting the land rent of a particular land quality class in alternative uses are not available (Gebremedhin & Swinton, 2003).

Capital constraints and imperfect markets exist in Ethiopia (Deininger et al., 2008; Holden & Ghebru, 2016) also challenge land use and production decisions. However, it can be assumed that the farming household[1] in Tigray that have been given the use right over land will allocate the land to different uses according to profitability in the particular use category (Bergeron & Pender, 1999; Djaenudin et al., 2016; Zhang et al., 2001; Zhao et al., 2018) when they produce and sell their outputs in the already available farm output and input markets according to the market prices and demand, and receive input supports and government intervention[2] to cope with capital constraints and imperfect markets (Dorward et al., 2004; Krueger, 1990; Stiglitz, 1989).

Hence, analysis of the behaviour of land use allocation in Tigray for this study is based on a profit-maximization theoretical model derived in the Appendix A. In this modelling it is assumed that farmers will allocate their homogenous land area among

different uses according to the profitability of each use, given their constraints and preferences.

Empirical model and estimation

This study specifies a linear equation for each land use/cover choice: which depends on the profitability of each use, given the constraints and preferences of farmers. The profitability of each use is determined by the community level price of each land use output, the characteristics of each plot allocated to each land use, and the household and community level factors that affect land use and production decisions.

The dataset used for this study is a plot-level three-wave panel dataset, which contains information on the area allocated to each land use (arranged in a nx1 vector grouped by land use type), and on the prices, plot characteristics, and household and community factors (arranged in a nxm matrix of independent variables with block diagonal structure) for a sample of farmers in Tigray region. The dataset also has also made to have a hierarchical structure: plots are nested within sustainability groups, and sustainability groups are nested within land use types. The degree of sustainability of a plot in a land use type is measured by the number of waves plots are observed in. Such structure of the dataset allows the study to control for unobserved heterogeneity across plots and sustainability groups, and to estimate separate land use/cover equations.

The study assumes that the error terms are correlated across different land uses, sustainability groups, and plots due to the competition for land among different uses and the unobserved plot specific heterogeneity. The study estimates a three-level mixed-effects model using the maximum likelihood method. The model accounts for the correlation of error terms within plots over sustainability group and across different land uses. The model also captures the unobserved heterogeneity at different levels of the hierarchy, sustainability group and plot. Our procedure estimated each land use equations

(cropland, pasture, fallow, forest, and other land uses) separately and software used for estimation is Stata version 17. For more details on the empirical model specification and estimation, please refer to Appendix B.

Empirical model and estimation

This study specifies a linear equation for each land use/cover choice: which depends on the profitability of each use, given the constraints and preferences of farmers. The profitability of each use is determined by the community level price of each land use output, the characteristics of each plot allocated to each land use, and the household and community level factors that affect land use and production decisions.

Land use/cover Change detection

We used two methods in broad to detect and analyse LULC change in Tigray between 1986 and 2016: Growth rate and transition matrix analysis. They are suitable for LULC change analysis, as they can handle complex and non-linear phenomena, such as land cover dynamics (Ewunetu et al., 2021; Munthali et al., 2019). Growth rate measures the rate of change of each LULC category over time, and transition matrix analysis shows the proportions of each LULC category that changed or persisted over time. Using the transition matrix analysis, we have conducted: component of change analysis, vulnerability to change analysis, and key signal of change analysis. Component of change measures the gross loss, gross gain, net change, and swap change for each category. Vulnerability to change measures the persistence indices for each category to show their vulnerability to loss, gain, or net change. Key signal of change identifies systematic transitions that deviate from random processes. These techniques are based on the approaches of Briones & Varas (2016), Ewunetu et al. (2021), Pontius et al. (2004), and Alo & Pontius Jr (2008). For details of the arguments and mathematical formulations of

each method and technique, please refer to Appendix C.

Results

Land use choice analysis

We analyse land allocation to land uses. We describe our model variables. Land without market value is allocated based on profitability which depends product prices (Djaenudin et al., 2016; Zhang et al., 2000). Land allocation also depends on land conditions, and socioeconomic and environmental factors (Bergeron & Pender, 1999; Zhao et al., 2018).

Descriptive statistics

About 72% Tigray farmers who decide land allocation are illiterate and have one economically active for each dependent in their five-member households. However, 77% of farmers also engage in [illegible]-farm activities. Their main livelihood is crop farming, owning and cultivating 87% of their land on an average of two to three plots in different locations[3]. In addition, they live on average 1 km away from their plots, which 46% have fair soil quality, 21% are vertosol type, and have high elevation and moderate slope[4]. Despite their low asset ownership and income, they benefit from extension services and have experienced income growth in 2012 to 2016. Nevertheless, they face challenges such as long distances to markets and roads, unstable prices, climate change and high population density. Moreover, the region falls below the national average of annual rainfall. Table 2 depicts description of the main variables of the study.

Table 2. Descriptive statistics for discrete and continuous descriptors by wave and pooled. The table shows the percentage for discrete descriptors and the mean and standard deviation for continuous descriptors.

Variables	Pooled		Waves					
			I		II		III	
	Mean	SD	Mean	SD	Mean	SD	Mean	SD
**Discrete**								
Plot descriptors								
Soil type is vertosol (Yes=1)	21.53	-	22.18	-	21.32	-	21.11	-
Soil quality: Good	26.16	-	27.94	-	26.30	-	24.39	-
Soil quality: Fair	46.34	-	42.53	-	42.56	-	53.26	-
Soil quality: Poor	27.50	-	29.53	-	31.14	-	22.36	-
Land tenure: owned	95.18	-	94.48	-	95.66	-	95.41	-
Land tenure: Tenanted	1.87	-	2.07	-	2.06	-	1.51	-
Land tenure: Sharecropped	2.19	-	1.14	-	2.28	-	3.09	-
Land tenure: Irregular cultivated	0.76	-	2.32	-	0.00	-	0.00	-
Household descriptors								
Literate household head (Yes=1)	27.91	-	26.06	-	25.07	-	32.20	-
Access to extension (Yes=1)	98.09	-	97.39	-	98.21	-	98.59	-
Nonfarm participation (Yes=1)	77.01	-	82.41	-	70.45	-	78.53	-
**Continuous**								
Plot descriptors								
Average plot slope (%)	12.62	10.96	13.31	11.37	12.57	11.02	12.03	10.48
Average plot elevation (m)	1943.08	540.17	1960.48	538.09	1922.50	536.61	1945.33	544.85
Plot Dist. To residence (kms)	1.02	15.55	1.24	26.79	0.96	3.67	0.87	2.28
Household descriptors								
Household size	4.92	2.28	4.95	2.23	4.95	2.29	4.88	2.31
Dependency ratio	1.16	0.90	1.20	0.95	1.13	0.88	1.14	0.88
Dist. to the nearest market (kms)	57.87	27.42	58.43	27.37	57.37	27.38	57.87	27.56
Dist. to nearest Major Road (kms)	17.98	16.50	17.97	16.56	18.14	16.45	17.85	16.53
Asset index	0.08	0.13	0.02	0.07	0.09	0.12	0.12	0.15
Annual income (00 Birr)	241.95	950.08	64.08	164.85	149.45	539.48	483.73	1466.66
Livestock (TLU)	3.58	4.52	3.28	3.45	3.03	3.38	4.36	5.97
Total size (hectare)	0.51	0.19	0.50	0.19	0.52	0.21	0.52	0.23
Proportion of crop land	0.87	0.18	0.88	0.18	0.87	0.17	0.86	0.20
Proportion of pasture land	0.10	0.10	0.10	0.10	0.11	0.12	0.08	0.08
Proportion of fallow land	0.24	0.23	0.20	0.21	0.27	0.22	0.26	0.25
Proportion of forest land	0.07	0.09	0.05	0.05	0.08	0.07	0.09	0.13
Proportion of other land uses	0.15	0.28	0.12	0.23	0.16	0.30	0.17	0.31
Average no of plots owned	2.52	0.53	2.24	0.74	2.18	0.74	2.2	0.83
Climate change risk perception+	0.24	0.36	0.14	0.30	0.17	0.33	0.40	0.39
Community descriptors								
Average cropland output price*	65.29	184.66	90.57	317.29	10.26	9.38	94.52	30.67
Average pasture land output price*	14.23	16.95	20.03	24.61	23.61	9.70	2.56	1.02
Average forest land output price*	229.88	197.05	73.75	26.16	100.07	26.01	442.37	135.80
Population density (per km^2)	738.7	2586.66	579.15	2026.4	747.91	2578.5	868.36	2994.99
Proportion of Agr. within 1 km buffer	0.36	0.23	0.37	0.23	0.35	0.22	0.36	0.22
Annual mean precipitation (mm)	701.74	104.78	702.75	105.15	701.85	106.69	700.75	102.91
**Households**	**996**		**307**		**335**		**354**	
**Plots**	**2,211**		**689**		**730**		**792**	

Note: Prices with * are quantity weighted average producer prices (Birr/kg). For crop land, products were mainly cereals; for pasture, meat, milk & milk products; for forest, fuelwood, charcoal & honey.

Climate change perception index is constructed by combining individuals' climate change beliefs, personal experience with extreme weather events, its frequency, severity, and associated livelihood losses.

Dependency ratio is derived by dividing the number of dependents (people under 18 or over 64) by the number of working-age people (people between 18 and 64) in the household.

Source: Panel data from the WB LSMS-ISA survey; own cross-sectional survey

Determinants of land use allocation

We used a mixed model to estimate five land use equations with STATA. Table 4 shows the maximum likelihood results. The LR and Wald tests support a three-level mixed model (intercept heterogeneity) and a joint fit. The AIC in Table 4 and annexed Table 7

showed the robustness of results of mixed model compared to separate OLS for each land use equations. Land use equation residuals are also found correlated supporting our specification (Table 3).

Table 3. Correlation Matrix of Residuals of Land Use Equations: After mixed model estimation

	Cropland	Pasture	Fallow	Forest	Others
Cropland	1.0000				
Pasture	0.2207	1.0000			
Fallow	-0.0939	0.0523	1.0000		
Forest	-0.1236	-0.0774	-0.3623	1.0000	
Others	-0.0232	-0.1778	-0.1979	-0.4334	1.0000

The results (Table 4) show that plot-level factors such as elevation, distance to residence, soil type and quality, and tenure affect land use in Tigray. Higher altitude increases fallow and pasture. Longer distance reduces forest and other land uses. Vertosol soil increases fallow and decreases forest land use compared to other types. Fair or poor soil quality reduces cropland and other land uses compared good soil quality while pasture is negatively related to poor soil quality. Tenanted land increases cropland and other land uses compared to owned land.

Household-level factors also affect land allocation. Increase in plot size increases all land uses, while increase in number of plots decreases them. Increase in dependency ratio increases fallow and decreases cropland and other land uses. Being literate head and increase in asset index and climate change perception decrease cropland, while higher asset index increases other land uses. The longer residence distance to market decreases cropland, fallow, and pasture, while longer distance to road decreases other land uses. Livestock ownership and income decrease pasture and forest, respectively.

Table 4. Mixed-effects model for land use share equations. The table shows the coefficients and standard errors for variables at plot, household and community levels that affect the land use shares.

Land use equation		Cropland	Pasture	Fallow	Forest	Others
Plot level factors	Slope (%)	0.0010	-0.0004	0.0007	-0.0005	-0.0005
		(0.0007)	(0.0007)	(0.0011)	(0.0007)	(0.0008)
	Elevation (m)	0.00001	0.0001	0.0001	0.00004	0.00002
		(0.00002)	(0.00002) **	(0.00003) **	(0.00003)	(0.00002)
	Dist. to residence (kms)	0.0003	-0.0068	-0.0006	-0.0149	-0.0072
		(0.0007)	(0.0076)	(0.0050)	(0.0064) **	(0.0030) **
	Soil type is vertosol (Yes=1)	-0.0149	0.0026	0.0919	-0.0316	-0.0195
		(0.0136)	(0.0189)	(0.0297) ***	(0.0188) *	(0.0190)
	Soil quality: Fair	-0.0218	0.0032	-0.0119		-0.0762
		(0.0131) *	(0.0131)	(0.0284)		(0.0182) ***
	Soil quality: Poor	-0.0300	-0.0257	0.0279		-0.0951
		(0.0149) *	(0.0139) *	(0.0318)		(0.0203) ***
	Land tenure: Tenanted	0.1104	-0.0150	-0.0661		0.2996
		(0.0307) ***	(0.0504)	(0.0735)		(0.0344) ***
	Land tenure: Sharecropped	0.0204	0.0236	-0.0594		0.0531
		(0.0284)	(0.0382)	(0.0633)		(0.0391)
Household level factors	Literate house head (Yes=1)	-0.0245	-0.0120			0.0194
		(0.0117) **	(0.0124)			(0.0164)
	Dependency ratio	-0.0156	0.0038	0.0287		-0.0144
		(0.0057) ***	(0.0054)	(0.0112) **		(0.0079) *
	Ln of total area (hectare)	0.0657	0.0660	0.1101	0.0413	0.0378
		(0.0045) ***	(0.0043) ***	(0.0096) ***	(0.0065) ***	(0.0085) ***
	No of plots owned	-0.0058	-0.0058	-0.0166	-0.0056	-0.0293
		(0.0014) ***	(0.0012) ***	(0.0022) ***	(0.0017) ***	(0.0016) ***
	Livestock (TLU)		-0.0039			
			(0.0015) ***			
	Asset index	-0.0940		-0.0861		0.1412
		(0.0475) **		(0.1219)		(0.0624) **
	Ln of annual income	0.0031			-0.0195	
		(0.0034)			(0.0053) ***	
	Dist. to nearest market (km)	-0.0005	-0.0006	-0.0026	0.0013	0.0005
		(0.0003) *	(0.0003) *	(0.0007) ***	(0.0021)	(0.0004)
	Dist. to Major Road (km)	0.0006	0.0009	0.0013	0.0022	-0.0018
		(0.0004)	(0.0006)	(0.0010)	(0.0029)	(0.0006) ***
	Climate change perception index	-0.0299			-0.0135	0.0131
		(0.0154) *			(0.0253)	(0.0213)
Community level factors	Average output price (Birr)	-0.00003	-0.0004		0.0003	
		(0.00003)	(0.0003)		(0.0001) ***	
	Population density (per km^2)	-0.000002	-0.00001	0.0010	0.000003	0.00002
		(0.000002)	(0.000005)	(0.00001)	(0.000004)	(0.00003) ***
	Annual mean Rainfall (mm)		0.0000		-0.0007	
			(0.0001)		(0.0004) **	
	Prop. of agri. in 1 km buffer				-0.0864	
					(0.0808)	
	Livelihood Zone: ALL	-0.0195	-0.0152	0.0109		-0.0885
		(0.0235)	(0.0297)	(0.0490)		(0.0326) ***
	Livelihood Zone: CMC	0.0334	0.0225	-0.0645	-0.0504	-0.0386
		(0.0205)	(0.0251)	(0.0537)	(0.1280)	(0.0293)
	Livelihood Zone: EDM	0.0245	0.0344	-0.0959	0.0306	-0.0779
		(0.0233)	(0.0514)	(0.0807)	(0.0929)	(0.0324) **
	Livelihood Zone: EPL	-0.0547	-0.0258	-0.0753	0.0112	0.0955
		(0.0224) **	(0.0193)	(0.0464)	(0.0881)	(0.0294) ***
	Livelihood Zone: HSS	-0.0434	0.0307	0.0525		-0.0872
		(0.0267)	(0.0348)	(0.0579)		(0.0359) **
	Livelihood Zone: WCT	-0.0075	-0.0087	0.0686	0.0085	0.0766
		(0.0229)	(0.0220)	(0.0489)	(0.0757)	(0.0317) **
	Constant	0.9957	0.3053	0.5825	0.7497	0.5368
		(0.0545) ***	(0.0888) ***	(0.1114) ***	(0.2588) ***	(0.0720) ***
Observations = 2.211		♦Wald Chi^2=34141.47 ***		°LR = 444.36 ***		AIC=-1612.53

Note: ♦Joint model fitness Chi2 test, °LR test vs. linear model (Chi2 test); * p<0.1; ** p<0.05; *** p<0.01, Standard errors are in parenthesis.

Source: Panel data from the WB LSMS-ISA survey; own cross-sectional survey

Community-level factors such as forest product price, precipitation, population density, and livelihood zone variations influence land use. Forest product price increases

forest, while precipitation decreases it. Population density increases other land uses. EPL, compared to other livelihood (OTH), allocate less land to cropland while livelihood zone variations have mixed effect on allocation to others land use.

Land cover change

Spaciotemporal distribution of LULC categories

The study showed spatial and temporal variation of LULC (Figures 2(a); (b)). We calculated the percentage of each landscape in Tigray and its main LHZs. Figure 2(c) show that LULC types changed over time, except for cropland, which was 20% in Tigray from 1986 to 2016. In 1986, bare land was the most common LULC category in Tigray (38%), followed by natural vegetation (31%), which included woodland, forest, bushland, and shrubs (called forest from now on). This pattern was also seen in ALL, CMC, WCT, and OTH. But in HSS, forest was more than bare land (42%), and in EPL, cropland was the main category (33%). EDM had more cropland (30%) next to EPL, while ALL had more forest (34%) next to HSS.

Forest and bare land decreased in Tigray and most LHZs except CMC and EPL in 2016. CMC had more forest (36%) than in 1986 (28%), while EPL kept its bare land. Pasture and other land uses increased in Tigray and most LHZs in 2016, but pasture dropped by 84% in CMC and 40% in EDM. The order of LULC types did not change much from 1986 to 2016, but forest dominance shifted from HSS and ALL to CMC and WCT. EDM and EPL stayed the top cropland LHZs in 2016.

Figure 2. Land cover maps and percentages in Tigray for 1986 and 2016. (a) Map of land cover types by LHZs in 1986. (b) Map of land cover changes by LHZs in 2016. (c) Percentages of land cover types by LHZs for 1986 and 2016, based on (a) and (b). Source: Author's own work using ArcGIS and LandSat imagery.

The LULC trend

Section 3.1.1 shows significant changes in all landscapes over 30 years, with low persistence from 37.05% in ALL to 61.28% in EDM (Figure 3(a)). Tigray had less forest (19%) and bare land (13%), but more pasture (104%) and other land uses (84%). Forest degraded most in HSS (43%) and ALL (29%) but recovered in CMC (27%). Pasture expanded most in HSS (threefold) and ALL (twofold), while cropland expanded only in HSS (21%) and EDM (19%).

The LULC inter category transition

Component of change. We quantified landscape transitions with respect to persistence, using gross losses and gains, net change, swap, and total change. These components were derived from the transition matrices and shown in Figure 3(b) and Figure 4(a). Bare land and forest had the top two gross losses and gains, but gross losses were higher than gross gains in each category, resulting in a net loss. Forest had the highest net change (5.73%), swap (25%), and total change (31%), followed by pasture (Figure 3(b) and Figure 4(a)).

Figure 3. Land cover trend and net change in Tigray by LHZ for 1986-2016. (a) Trend change showing percentage of each land cover type in each LHZ over time. Percentage values by LHZ legend indicate persistence of total landscape. (b) Net percentage change of each land cover type in each LHZ from 1986 to 2016. Source: Author's own work using ArcGIS and LandSat imagery.

LULC dynamics across LHZs showed a mixed pattern. Forest was the most dynamic in CMC, EPL and WCT, followed by bare land. Pasture was the most dynamic in ALL and WCT, followed by bare land. In EDM, cropland was the most dynamic, followed by bare land. In OTH, bare land was the most dynamic, followed by forest. In all LHZs, total change was swap dominated (Figure 4(a)). Most (86%) farming households in each LHZ also perceived such dynamism in cropland, pasture, and forest.

Vulnerability of LULC to dynamic. Figure 4(b) shows persistence indices of LULC

dynamics in Tigray. Except for cropland (relatively stable), all categories tended to transition from loss/gain to another category. Forest and bare land lost more area, while pasture and other land uses gained more area than they persisted. Pasture gained the most area (a total increment of 4.4 times or a net gain of 2.7 times its persistence), followed by other land uses (a total increment of twice or a net gain of 1.3 times its persistence). Forest lost the most area (150% lower than its persistence) compared with the rest of the categories.

Figure 4. Percentage total change and swamp change, and persistence indices of land cover types in Tigray by livelihood zones (LHZ) for 1986-2016. (a) Percentage total change (Tc) and percentage swamp change (S) of each land cover type by LHZ. (b) Persistence indices for each land cover type by LHZ, with Gain: Persistent (Gp), Loss: Persistent (Lp), and Net Change: Persistent (Np). Source: Author’s own work using ArcGIS and LandSat imagery.

LULC categories changed similarly across space (LHZs), except for forest and pasture in CMC. Forest gained more than it persisted (a total increment of 120% of its persistence), while pasture lost more than any other category (a total decrement of 17.7 times or a net loss of 15.6 times its persistence) in CMC (Figure 4(b)). Bare land had a lower tendency to change in all LHZs except in ALL, HSS, and WCT, despite having the greatest loss/gain and the second highest total change. This inconsistency can be explained by the size of LULC categories and their persistence levels. Pontius et al. (2004) suggested that the largest LULC categories may have large transitions by chance. We separated key and systematic patterns of change from any persistence level and land cover size to avoid misleading signals of transition and spurious landscape dynamics.

Detecting key signals of LULC change. Figure 5 shows how categories change based on gains and losses. Green up-arrows mean more positive changes than expected, red down-arrows mean more negative changes than expected, and yellow hyphens mean random changes. In Tigray and most LHZs, only two changes were random: transition between “*pasture and forest*” and the loss of “*other land uses to bare land*,”. In ALL, all changes

were systematic, while in CMC, many changes were random, especially to "*pasture*". The most important systematic transitions (highlighted light red in Figure 5) with conclusive evidence for a key signal of change (green up arrows for both losses and gains) were from "*cropland*" to "*forest*" and from "*forest*" to "*bare land*" in Tigray and LHZs, from "bare land" to "pasture" in ALL, HSS, WCT, and Tigray, and from "bare land" to "other land uses" in CMC, EDM, EPL, and OTH.

Figure 5. Transition map of land cover types in Tigray for 1986-2016, showing systematic and random transitions and key signals of change. (a_1) Transitions based on losses for Tigray. (a_2) Transitions based on gains for Tigray. (b_1) to (h_1) Transitions based on losses for each LHZ, ordered as ALL, CMC, EDM, EPL, HSS, WCT and OTH. (b_2) to (h_2) Transitions based on gains for each LHZ, ordered as ALL, CMC, EDM, EPL, HSS, WCT and OTH. Source: Author's own work using ArcGIS and LandSat imagery.

Table 5 shows that forest and bare land (net loss) and pasture (net gain) changed the most. In Tigray, forest (12.8%) lost to bare land (5.01% more) and pasture (6.26%) gained from bare land (2.74% more) than expected randomly. This was similar in ALL, HSS, and OTH, except in OTH where other land use gained from bare land dominantly. Another change was cropland to forest (3.61% more) in Tigray and all LHZs. EDM also lost more bare land to other land uses (2.27% more) unlike others.

Table 5. Key signals of change by LHZ based on most significant systematic transitions. The table shows the land cover types with top 6 dominant systematic transitions in each LHZ. Percentages are relative to total landscape.

Transition for the period 1986 to 2016	Livelihood Zone (LHZ)							
	**ALL**	**CMC**	**EDM**	**EPL**	**HSS**	**WCT**	**OTH**	**Tigray**
Crop land to Forest								
Observed (%)	6.15	8.95	3.45	9.27	2.75	10.84	6.12	5.84
Expected (%)	2.17	3.83	1.04	3.95	1.12	4.95	2.19	2.23
	(2.35)	(4.99)	(2.44)	(5.52)	(2.46)	(7.22)	(3.66)	(3.65)
Difference (%)	3.98	5.12	2.42	5.32	1.62	5.89	3.93	3.61
	(3.80)	(3.96)	(1.01)	(3.75)	(0.29)	(3.61)	(2.46)	(2.19)
Difference/Expected	1.8	1.34	2.33	1.35	1.45	1.19	1.79	1.62
	(1.62)	(0.79)	(0.41)	(0.68)	(0.12)	(0.50)	(0.67)	(0.60)
Forest to Bare land								
Observed (%)	18.41	9.30	4.60	9.57	20.38	15.69	9.62	12.98
Expected (%)	10.34	7.65	3.73	6.20	13.30	10.79	5.48	7.97
	(11.37)	(5.68)	(2.22)	(4.64)	(14.52)	(9.26)	(4.62)	(7.21)
Difference (%)	8.07	1.65	0.87	3.37	7.07	4.90	4.14	5.01
	(7.05)	(3.62)	(2.37)	(4.93)	(5.86)	(6.43)	(4.99)	(5.78)
Difference/Expected	0.7	0.22	0.23	0.54	0.5 (0.40)	0.40	0.75	0.63
	(0.62)	(0.64)	(1.07)	(1.06)		(0.69)	(1.08)	(0.80)
Bare land to pasture								
Observed (%)	17.07				11.64	4.48		6.26
Expected (%)	9.90				7.69	1.74		3.16
	(9.77)				(7.44)	(2.11)		(3.53)
Difference (%)	7.18				3.95	2.74		3.10
	(7.31)				(4.20)	(2.37)		(2.74)
Difference/Expected	0.73				0.51	1.57		0.98
	(0.75)				(0.56)	(1.12)		(0.78)
**Bare land to other uses**								
Observed (%)		6.66	6.29	6.04			6.89	
Expected (%)		3.06	3.57	2.69			4.58	
		(4.25)	(3.99)	(3.63)			(3.93)	
Difference (%)		3.60	2.72	3.36			2.31	
		(2.42)	(2.31)	(2.41)			(2.96)	
Difference/Expected		1.18	0.76	1.25			0.50	
		(0.57)	(0.58)	(0.66)			(0.75)	

Note: Values in brackets are based on gains and values off brackets are based on losses.
Source: Author's own work using ArcGIS and LandSat imagery.

Discussion

A mixed-effects model showed that plot-level factors significantly impacted land use allocation. Plots near residences were often used for forest or other land uses to prevent deforestation by outsiders (Etsay et al., 2019). Higher-altitude plots were more likely to be fallowed, possibly for animal feed (Bergeron & Pender, 1999). Soil quality and type influenced land use choices: better soils favored cropland and pasture and vertosols favored fallow land, agreeing with previous studies (Verburg et al., 2004; Vu et al., 2014). Tenanted land was more often used for cropland or other land uses than owned land, reflecting intension of tenants to maximize short-term land returns.

Household-level factors also significantly affected land use. More land use types, especially fallow land, were associated with larger and less fragmented plots. This indicates that farmers in Tigray may use most of their newly acquired land for fallowing (1.7 times greater) and leave their more fragmented land idle, both signifying probable capital constraints that impose restrictions on land use (Deininger et al., 2008). A higher dependency ratio increased fallow land and reduced cropland and other uses, possibly due to labor shortages in agriculture (Braimoh & Vlek, 2004). Higher head education, asset ownership, and climate change perception decreased cropland area, possibly indicating income diversification (O'Donoghue & Heanue, 2018; Wang et al., 2019; Zhao et al., 2018). Increased income/wealth increased demand for built-up land similar to other studies (Gatto, 2015; Li et al., 2016). Households perceiving climate change may shift their cropland to other categories as climate warming reduces arable land (Li et al., 2016).

Wealthier households or those with more livestock tended to use less forest and pastureland, respectively (Nguyen et al., 2017), suggesting that they undervalued the in-situ benefits of forests and preferred larger livestock herds in distant wild grazing areas (Son & Shrestha, 2008; Wolfersberger et al., 2015). Market and main road proximity increased the use of pasture, fallow land, and other land uses, indicating that market access and infrastructure availability influenced land use choices by creating opportunities for off-farm activities and livestock production (Verburg et al., 2004).

Product price, annual precipitation, and LHZ also affected land use allocation at the community level. Forest product price increased forest land use, possibly reflecting the profitability of forest products and the conservation incentive (Zhang et al., 2000). Annual precipitation decreased forest land use, possibly due to the suitability of wetter areas for agriculture in arid Tigray. Land use allocation differed across different LHZs, reflecting biophysical and socio-economic differences among them.

Change detection analysis also revealed LULC dynamics in Tigray and its main LHZs from 1986 to 2016, in line with Betru (2019). The results also indicated that bare land was the most dominant LULC in all landscapes except for EDM, where cropland dominated.

The most alarming change was the systematic conversion of forest (12.8%) to bare land in Tigray and all LHZs except for CMC, where forest cover increased. Forest cover decreased by 19% (0.7% per annum) in Tigray, resulting in a net loss of 6% of the total landscape. Forest degradation was most rapid in HSS (net loss of 18%), followed by ALL (net loss 10%). This indicates that forest was the most vulnerable and dynamic LULC category in Tigray, followed by pasture. These findings agree with previous studies that reported the ongoing deforestation and forest degradation in Tigray (Alemu et al., 2015b; Solomon et al., 2018) and Ethiopia (Gebeyehu et al., 2023; Regasa et al., 2021). This threatens the environment and the livelihoods of local communities through losses in soil quality, water availability, biodiversity, carbon sequestration, food, and resources. Solomon et al. (2019) stressed that forest cover dynamics in Tigray can affect ecosystem services and livelihood losses.

Besides forest loss, another notable change was the expansion of pasture (6.26%) into bare land in Tigray and most LHZs except for CMC and EDM, where pasture was abandoned. Pasture increased by 104% in share, resulting in a net gain of 6% of the total landscape. Pasture expansion was most rapid in HSS and ALL (net gain of 16% each). However, this did not offset the loss of forest cover to bare land. Cropland was relatively stable (almost zero net loss) but with a swap of 14% of the total landscape.

Despite these unfavorable changes, Farming households in Tigray and all LHZs showed commitment to systematic cropland afforestation, but only CMC saw a net increase in forest cover (8%) by afforesting cropland and pasture. Several studies back

cropland-to-forest change in Tigray and Amhara (Belay et al., 2015; Gedefaw et al., 2020). EDM also changed bare land to other uses, likely due to urban growth around Mekelle city and other growing towns in the LHZ.

Conclusion and policy implication

Conclusion

Using socioeconomic panel and Landsat data, this study examined how various factors influenced land use choice and land cover change in Tigray and its main livelihood zones from 1986 to 2016. The study showed that land use choice depended on plot, household, and community characteristics, such as plot distance, size and number, household income, forest price, and rainfall. It was also implied that land use allocation was affected by capital constraints that exist, with more fallowing newly acquired land and leaving more fragmentated land idle. This damaged the environment and livelihoods.

The study also detected the most important environmental changes in the study area. One was the fast and systematic forest loss (12.8%) to bare land. Forest loss was worst in HSS and ALL zones. The only exception was CMC zone, where forest increased due to cropland and pasture conversion to forest in form of plantation. This means that some farmers gave up their cropland for afforestation, despite its negative impact on their food and income. Another was the fast and systematic pasture gain (6.26%) from bare land. Pasture gain was best in HSS and ALL zones. However, pasture loss happened in CMC and EDM zones. Moreover, forest to bare land conversion was more than bare land to pasture conversion, endangering both the environment and livelihoods.

Policy implication

Based on the findings of this study, we suggest the following policy recommendations

for sustainable land use planning and management in Tigray and its livelihood zones:

- Implement supports and incentives such as improved market linkages, access to roads, facilities, services, forestry subsidies, and land tenure security to increase the benefit of farming households from forests, and carefully manage cropland and pasture reduction to balance the needs of both the environment and local communities.
- Implement land consolidation programs that reduce land fragmentation to increase land use efficiency and productivity.
- Adopt participatory approaches that involve local communities and stakeholders to prioritize forest regeneration on bare land in reafforestation efforts.
- Further analysis is needed to assess the effectiveness of land use policies or strategies in mitigating the environmental impacts of LULC changes in Tigray and its main LHZs.

Reference

Abbink, J. (2011). Ethnic-based federalism and ethnicity in Ethiopia: Reassessing the experiment after 20 years. *Journal of Eastern African Studies*, *5*(4), 596–618.

Abere, T., Adgo, E., & Afework, S. (2020). Trends of land use/cover change in Kecha-Laguna paired micro watersheds, Northwestern Ethiopia. *Cogent Environmental Science*, *6*(1), 1801219.

Alemu, B., Garedew, E., Eshetu, Z., & Kassa, H. (2015a). Land use and land cover changes and associated driving forces in north western lowlands of Ethiopia. *International Research Journal of Agricultural Science and Soil Science*, *5*(1), 28–44.

Alemu, B., Garedew, E., Eshetu, Z., & Kassa, H. (2015b). Land use and land cover changes and associated driving forces in north western lowlands of Ethiopia. *International Research Journal of Agricultural Science and Soil Science*, *5*(1), 28–44.

Alo, C. A., & Pontius Jr, R. G. (2008). Identifying systematic land-cover transitions using remote sensing and GIS: the fate of forests inside and outside protected areas of Southwestern Ghana. *Environment and Planning B: Planning and Design*, *35*(2), 280–295.

Aredehey, G., Mezgebu, A., & Girma, A. (2020). The effects of land use land cover change on hydrological flow in Giba catchment, Tigray, Ethiopia. *Cogent Environmental Science*, *6*(1), 1785780.

Belay, K. T., Van Rompaey, A., Poesen, J., Van Bruyssel, S., Deckers, J., & Amare, K. (2015). Spatial analysis of land cover changes in Eastern Tigray (Ethiopia) from 1965 to 2007: Are there signs of a forest transition? *Land Degradation & Development*, *26*(7), 680–689.

Bergeron, G., & Pender, J. L. (1999). *Determinants of land use change: Evidence from a community study in Honduras*.

Betru, T. (2019). Progress, Challenges and Implications of Land Use/Land Cover Detection Methods in Ethiopia: A Review. *Journal of Agriculture and Environmental Sciences*, *4*(2).

Biørn, E., Lindquist, K.-G., & Skjerpen, T. (2002). Heterogeneity in returns to scale: A random coefficient analysis with unbalanced panel data. *Journal of Productivity Analysis*, *18*, 39–57.

BoPF. (2018). *Socio-economic profile of Tigray Region*. Bureau of Planning and Finance.

Braimoh, A. K., & Vlek, P. L. (2004). Scale-dependent relationships between land-use change and its determinants in the Volta Basin of Ghana. *Earth Interactions*, *8*(4), 1–23.

Briones, P. S., & Varas, A. S. (2016). Systematic transitions in land use and land cover in a pre-Andean subwatershed with high human intervention in the Araucania Region, Chile. *Ciencia e Investigación Agraria: Revista Latinoamericana de Ciencias de La Agricultura*, *43*(3), 396–407.

Brouwer, C., Goffeau, A., & Heibloem, M. (1985). *Irrigation Water Management: Training Manual No. 1—Introduction to Irrigation*. FAO. https://www.fao.org/3/R4082E/r4082e00.htm#Contents

Coates, J., Swindale, A., & Bilinsky, P. (2007). *Household Food Insecurity Access Scale (HFIAS) for measurement of food access: Indicator guide: Version 3*.

Deininger, K., Ali, D. A., Holden, S., & Zevenbergen, J. (2008). Rural Land Certification in Ethiopia: Process, Initial Impact, and Implications for Other African Countries. *World Development*, *36*(10), 1786–1812. https://doi.org/10.1016/j.worlddev.2007.09.012

Djaenudin, D., Oktaviani, R., Hartoyo, S., & Dwiprabowo, H. (2016). Modelling of land allocation behavior in Indonesia. *Procedia Environmental Sciences*, *33*, 78–86.

Dorward, A., Kydd, J., Morrison, J., & Urey, I. (2004). A Policy Agenda for Pro-Poor Agricultural Growth. *World Development*, *32*(1), 73–89. https://doi.org/10.1016/j.worlddev.2003.06.012

Etsay, H., Negash, T., & Aregay, M. (2019). Factors that influence the implementation of sustainable land management practices by rural households in Tigrai region, Ethiopia. *Ecological Processes*, *8*(1), 1–16.

Ewunetu, A., Simane, B., Teferi, E., & Zaitchik, B. F. (2021). Land cover change in the blue nile river headwaters: Farmers' perceptions, pressures, and satellite-based mapping. *Land*, *10*(1), 68.

Farley, J. (2008). The role of prices in conserving critical natural capital. *Conservation Biology*, *22*(6), 1399–1408.

Gatto, M. (2015). *Land-use dynamics, economic development, and institutional change in rural communities-Evidence from the Indonesian oil palm sector*.

Gebeyehu, A. K., Snelder, D., & Sonneveld, B. (2023). Land use-land cover dynamics, and local perceptions of change drivers among Nyangatom agro-pastoralists, Southwest Ethiopia. *Land Use Policy, 131*, 106745.

Gebreegziabher, Z., Mekonnen, A., Kassie, M., & Köhlin, G. (2020). Household Tree Planting in Tigrai, Northern Ethiopia: Tree Species, Purposes, and Tenure Security. *Land Use Policy*, *96*(C). https://econpapers.repec.org/article/eeelauspo/v_3a96_3ay_3a2020_3ai_3ac_3as0264837719309007.htm

Gebremedhin, B., Pender, J., & Tesfay, G. (2003). Community natural resource management: The case of woodlots in northern Ethiopia. *Environment and Development Economics*, *8*(1), 129–148.

Gebremedhin, B., & Swinton, S. M. (2003). Investment in soil conservation in northern Ethiopia: The role of land tenure security and public programs. *Agricultural Economics*, *29*(1), 69–84.

Gedefaw, A. A., Atzberger, C., Bauer, T., Agegnehu, S. K., & Mansberger, R. (2020). Analysis of land cover change detection in Gozamin District, Ethiopia: From remote sensing and DPSIR perspectives. *Sustainability*, *12*(11), 4534.

Hachoofwe, E. M. (2012). *Local ecological knowledge of trees on farms, constraints and opportunities for further integration in Tigray Region, northern Ethiopia: A case study of smallholder farmers in Abreha Wa Atsbeha and Adi gudom*.

Holden, S. T., & Ghebru, H. (2016). Land tenure reforms, tenure security and food security in poor agrarian economies: Causal linkages and research gaps. *Global Food Security*, *10*, 21–28. https://doi.org/10.1016/j.gfs.2016.07.002

Hurni, H., Abate, S., Bantider, A., Debele, B., Ludi, E., Portner, B., Yitaferu, B., & Zeleke, G. (2010). *Land degradation and sustainable land management in the highlands of Ethiopia*.

Krueger, A. O. (1990). Government Failures in Development. *Journal of Economic Perspectives*, *4*(3), 9–23. https://doi.org/10.1257/jep.4.3.9

Lee, S. Y. (1996). *Agricultural location theory and behaviors in market based on von Thunen's model*.

Li, X., Wang, Y., Li, J., & Lei, B. (2016). Physical and socioeconomic driving forces of land-use and land-cover changes: A Case Study of Wuhan City, China. *Discrete Dynamics in Nature and Society*, *2016*.

Mekuria, W., Veldkamp, E., Haile, M., Nyssen, J., Muys, B., & Gebrehiwot, K. (2007). Effectiveness of exclosures to restore degraded soils as a result of overgrazing in Tigray, Ethiopia. *Journal of Arid Environments*, *69*(2), 270–284.

Mesele, K., Ponamala, S., & Tigga, A. (2019). *Determinants of household food security in southern Tigray, northern Ethiopia*. 1463–1471.

Miheretu, B. A., & Yimer, A. A. (2018). Land use/land cover changes and their environmental implications in the Gelana sub-watershed of Northern highlands of Ethiopia. *Environmental Systems Research*, *6*, 1–12.

Munthali, M. G., Botai, J. O., Davis, N., & Adeola, A. M. (2019). *Multi-temporal analysis of land use and land cover change detection for Dedza district of Malawi using geospatial techniques*.

Newbold, T., Hudson, L. N., Hill, S. L., Contu, S., Lysenko, I., Senior, R. A., Börger, L., Bennett, D. J., Choimes, A., & Collen, B. (2015). Global effects of land use on local terrestrial biodiversity. *Nature*, *520*(7545), 45–50.

Nguyen, T. T., Nguyen, L. D., Lippe, R. S., & Grote, U. (2017). Determinants of farmers' land use decision-making: Comparative evidence from Thailand and Vietnam. *World Development, 89,* 199–213.

Nkonya, E., Anderson, W., Kato, E., Koo, J., Mirzabaev, A., von Braun, J., & Meyer, S. (2016). Global cost of land degradation. *Economics of Land Degradation and Improvement–A Global Assessment for Sustainable Development*, 117–165.

O'Donoghue, C., & Heanue, K. (2018). The impact of formal agricultural education on farm level innovation and management practices. *The Journal of Technology Transfer, 43,* 844–863.

Pontius Jr, R. G., Shusas, E., & McEachern, M. (2004). Detecting important categorical land changes while accounting for persistence. *Agriculture, Ecosystems & Environment, 101*(2–3), 251–268.

Regasa, M. S., Nones, M., & Adeba, D. (2021). A review on land use and land cover change in Ethiopian basins. *Land*, *10*(6), 585.

Solomon, N., Hishe, H., Annang, T., Pabi, O., Asante, I. K., & Birhane, E. (2018). Forest cover change, key drivers and community perception in Wujig Mahgo Waren forest of northern Ethiopia. *Land*, *7*(1), 32.

Son, N., & Shrestha, R. P. (2008). GIS-assisted land evaluation for agricultural development in Mekong delta, southern Vietnam. *J. Sus. Dev. Africa*, *10*(2), 875–895.

Stiglitz, J. E. (1989). Markets, Market Failures, and Development. *The American Economic Review*, *79*(2), 197–203.

Tadesse, L., Suryabhagavan, K., Sridhar, G., & Legesse, G. (2017). Land use and land cover changes and Soil erosion in Yezat Watershed, North Western Ethiopia. *International Soil and Water Conservation Research*, *5*(2), 85–94.

Temesgen, G., Amare, B., & Silassie, H. G. (2014). Land degradation in Ethiopia: Causes, impacts and rehabilitation techniques. *Journal of Environment and Earth Science*, *4*(9), 98–104.

Tesfaye, S., Guyassa, E., Joseph Raj, A., Birhane, E., & Wondim, G. T. (2014). Land use and land cover change, and woody vegetation diversity in human driven landscape of Gilgel Tekeze Catchment, Northern Ethiopia. *International Journal of Forestry Research*, *2014*.

Turner, B. L., Lambin, E. F., & Reenberg, A. (2007). The emergence of land change science for global environmental change and sustainability. *Proceedings of the National Academy of Sciences*, *104*(52), 20666–20671.

Turner, B. L., Skole, D., Sanderson, S., Fischer, G., Fresco, L., & Leemans, R. (1995). Land-use and land-cover change: Science/research plan. *[No Source Information Available]*.

Van der Veen, A., & Gebrehiwot, T. (2011). Effect of policy interventions on food security in Tigray, Northern Ethiopia. *Ecology and Society*, *16*(1).

van Schrojenstein Lantman, J., Verburg, P., Bregt, A., & Geertman, S. (2011). Land-use modelling in planning practice. *Land-Use Modell. Plann. Pract*, *101*, 35–57.

Verburg, P. H., Crossman, N., Ellis, E. C., Heinimann, A., Hostert, P., Mertz, O., Nagendra, H., Sikor, T., Erb, K.-H., & Golubiewski, N. (2015). Land system science and sustainable development of the earth system: A global land project perspective. *Anthropocene*, *12*, 29–41.

Verburg, P. H., Schot, P. P., Dijst, M. J., & Veldkamp, A. (2004). Land use change modelling: Current practice and research priorities. *GeoJournal*, *61*, 309–324.

Vu, Q. M., Le, Q. B., Frossard, E., & Vlek, P. L. (2014). Socio-economic and biophysical determinants of land degradation in Vietnam: An integrated causal analysis at the national level. *Land Use Policy*, *36*, 605–617.

Wang, W., Dong, Y., Luo, R., Bai, Y., & Zhang, L. (2019). Changes in returns to education for off-farm wage employment: Evidence from rural China. *China Agricultural Economic Review*, *11*(1), 2–19.

WoldeYohannes, A., Cotter, M., Kelboro, G., & Dessalegn, W. (2018). Land use and land cover changes and their effects on the landscape of Abaya-Chamo Basin, Southern Ethiopia. *Land*, *7*(1), 2.

Wolfersberger, J., Delacote, P., & Garcia, S. (2015). An empirical analysis of forest transition and land-use change in developing countries. *Ecological Economics*, *119*, 241–251.

Yang, B., Duan, Y., & Zhao, Q. (2022). The effect of land fragmentation on farmers' rotation behavior in rural China. *Frontiers in Environmental Science*, *10*. https://www.frontiersin.org/articles/10.3389/fenvs.2022.1042755

Yaynemsa, K. G. (2022). Trends in Plant Ecology Research in Ethiopia. In K. G. Yaynemsa (Ed.), *Plant Biodiversity Conservation in Ethiopia: A Shift to Small Conservation Reserves* (pp. 153–167). Springer International Publishing. https://doi.org/10.1007/978-3-031-20225-4_11

Yesuph, A. Y., & Dagnew, A. B. (2019). Land use/cover spatiotemporal dynamics, driving forces and implications at the Beshillo catchment of the Blue Nile Basin, North Eastern Highlands of Ethiopia. *Environmental Systems Research*, *8*(1), 1–30.

Zhang, Y., Uusivuori, J., & Kuuluvainen, J. (2000). Econometric analysis of the causes of forest land use changes in Hainan, China. *Canadian Journal of Forest Research*, *30*(12), 1913–1921.

Zhang, Y., Uusivuori, J., & Kuuluvainen, J. (2001). *Econometric analysis of the causes of forest land use changes in Hainan, China*. EEPSEA, Singapore, SG.

Zhao, X., Pu, J., Wang, X., Chen, J., Yang, L. E., & Gu, Z. (2018). Land-use spatio-temporal change and its driving factors in an artificial forest area in Southwest China. *Sustainability*, *10*(11), 4066.

Appendix

A. Theoretical Model and Notation

Economic theory postulates farming households, facing inexistence of markets for land, allocate their homogenous land to different uses according to the profitability of a specific land use. Land profitability directly or indirectly depends on output and input prices, physical and location conditions of land, socioeconomics and other natural environment factors (Bergeron & Pender, 1999; Djaenudin et al., 2016; Zhang et al., 2001; Zhao et al., 2018). Assume now landholder (farming household) wants to maximize profit from different land uses on homogenous land (plot[5]) under his control by allocating it optimally between k different uses. Thus, a farming household's profit maximization problem constrained by the total homogenous land area under his control can be presented as:

$$\underset{A^k}{Max}\, \pi\,(p^k, A) = \sum_{k=1}^{n} [p^k y^k(A^k, E^k, z) - cE^k]$$

$$St \sum_{k=1}^{n} A^k \leq A, A^k, E^k \geq 0 \qquad \text{(A-1)}$$

Where y^k is output for land use k, p^k is farmgate price for output y^k, and A, A^k, E^k and c, respectively, are total plot area, land area allotted to k^{th} use (k=1, …, n) by each plot, aggregate input and unit cost of aggregate input used to produce output y^k. z represent physical and location conditions of land, household socioeconomics and community level factors (like natural environment).

Assuming marginal product of land and aggregate input are positive but decreasing in each land use category i.e., $\frac{\partial y_k}{\partial A_k} > 0, \frac{\partial^2 y_k}{\partial {A_k}^2} < 0 \; and \; \frac{\partial y_k}{\partial E_k} > 0, \frac{\partial^2 y_k}{\partial {E_k}^2} < 0,$ we setup Lagrangean for the maximization as:

$$L = \sum_{k=1}^{n} [p^k y^k(A^k, E^k, z) - cE^k] + \lambda(A - \sum_{k=1}^{n} A^k) \qquad \text{(A-2)}$$

The first order conditions are, then,

$$\frac{\partial L}{\partial A^k} = p^k \frac{\partial y^k}{\partial A^k} - \lambda \leq 0 \longrightarrow \left(p^k \frac{\partial y^k}{\partial A^k} - \lambda\right) A^k = 0 \qquad \text{(A-3)}$$

$$\frac{\partial L}{\partial E^j} = p^k \frac{\partial y^k}{\partial E^k} - c \leq 0 \longrightarrow \left(p^k \frac{\partial y^k}{\partial E^k} - c\right) E^k = 0 \qquad \text{(A-4)}$$

$$\frac{\partial L}{\partial \lambda} = (A - \sum_{k=1}^{n} A^k) \geq 0 \longrightarrow (A - \sum_{k=1}^{n} A^k)\lambda = 0 \qquad \text{(A-5)}$$

The marginal income from additional land to be used in Eq. (A-3) ($p^k \frac{\partial y^k}{\partial A^k}$) represent shadow land rents. Each household optimally allocate land in a way these land rents for a homogenous land (plot) are equal for each land use type. As implied by the first order condition, such optimal allocation of land area is, then, function of output price, aggregate

input price, total plot area (A) and other plot, household, and community level factors. Thus, land use/cover equations for the farming household considering plot owned can then be presented as:

$$A^{k*} = A^{k*}(p^k, c, z; A) \qquad \text{(A-6)}$$

B. Model specification and estimation

For estimation purpose, this study assumed a linear specification for the land use/cover equations. Furthermore, in situations where price data for aggregate inputs are absent, their effects can be proxied by the effects of local population level and access to markets as local input prices are mostly affected by local demand such as land and labor (Bergeron & Pender, 1999). Given plot-level three-wave panel dataset and these assumptions, land use/cover equations for each land use choice k (k=cropland, pasture, fallow, forest, and other land uses) on plot *i* in time *w* can be empirically specified as:

$$A_{iw}^{k} = \alpha^{k} + \beta^{k} p_{w}^{k} + \phi^{k} {z_{iw}}^{k} + \psi^{k} z_{w} + \varepsilon_{iw}^{k} \qquad \text{(B-7)}$$

A_{iw}^{k} is land area of the ith plot allocated to k^{th} use in time w, p_{w}^{k} is community level price of kth land use output in time w, ${z_{iw}}^{k}$ is characteristics of ith plot allocated to k^{th} land use in time w, and z_w is household and community level factors in time w. α^k, β^k, ϕ^k, and ψ^k are parameters to be estimated for the kth land use whereas ε_{iw}^{k} are correlated error terms.

Two possible sources for error term correlations can be noted (i) competition for plotland among different land uses or/and (ii) unobserved plot specific heterogeneity. Regarding the first, land use allocation considers tradeoff in land uses and resulting benefits. Tradeoff in land uses means that farmers must choose how to allocate their limited land area among different uses, and that each use has different benefits and costs[6].

Hence any decision to use the land cannot be separated from each other i.e., the decision to increase the area of a land use will influence other land use decisions. This requires estimating land use/cover equations that can account for the correlation among errors of land use. Whereas unobserved plot heterogeneity in each land use/cover equations can be separated from the gross disturbance by adopting random/fixed effect specification in each equation.

To complete our specification, let's have clear picture of the structure of our dataset. Our dataset consists of data on n plots of land that are grouped by their land use types. Each plot can have at least one of k possible land use types. We measure the dependent variables (A_{iw}^{k}) for each plot in each land use type in a vector (nx1)[7]. The independent variables are arranged in a matrix (nxm) with block diagonal structure, meaning that each land use type has its own set of explanatory variables on the diagonal[8]. We observe the plots over three waves, but not all plots are observed in all waves. Some plots may switch from one land use type to another, creating gaps in the data. This makes our panel data unbalanced[9]. To account for this, we follow the method of Biorn et al. (2002) and group the plots by the number of waves they are observed in. This reflects the degree of sustainability of a plot in a land use type. A plot that stays in the same land use type for all three waves[10] is more sustainable than a plot that switches. Therefore, our dataset has a hierarchical structure: plots are nested within sustainability groups, and sustainability groups are nested within land use types.

We used unbalanced panel data to fit land use/cover equations that account for the potential correlation of errors and the plot specific effects that are not observed. For this purpose, we respecify Eq. B-7 as a three-level mixed-effects model as follows:

$$A_{ijk} = \alpha_k + \phi z_{ijk} + c_{ij} + c_{ijk} + \mu_{ijk} \qquad \text{(B-8)}$$

Where A_{ijk} is the area of land allocated to use (k) by plot (i) in sustainability group (j), z_{ijk} is a vector of determinants (plot, household and community level factors like price of outputs of kth land use) for land use (k) by plot (i) in sustainability group (j), α_k is the intercept for land use group (k), ϕ is a vector of fixed effects coefficients, c_{ij} is a random intercept for sustainability group (j) within land use type (k), c_{ijk} is a random intercept for plot (i) within sustainability group (j) and land use type (k), and μ_{ijk} is the error term with $\mu_{ijk} \sim IIN(0, \sigma^2{}_u)$. The random intercepts are uncorrelated with error terms and assumed to be normally distributed with mean zero and variance-covariance matrices to be estimated.

The fixed effects coefficient ϕ measures the average effect of the determinants on the land use area across all plots, sustainability groups, and land use types. The random effects coefficients c_{ij}, and c_{ijk} capture the unobserved heterogeneity at different levels of the hierarchy, such as sustainability group and plot.

We assume that the vector of error terms follows $\mu \sim N(0, \Omega)$, which captures the possible correlation between the errors of different land use equations. The error covariance matrix has the following form:

$$\Omega = \begin{bmatrix} \sigma_1^2 & \cdots & \sigma_{lk} \\ \vdots & \ddots & \vdots \\ \sigma_{kl} & \cdots & \sigma_k^2 \end{bmatrix}$$

where Ω is the error covariance matrix, σ_k^2 is the variance of the error term for land use type (k), and σ_{kl} is the covariance of the error terms for land use types (k) and (l). Note that Ω is symmetric and positive definite.

We estimated the parameters of our three-level mixed-effects model using the maximum likelihood method. We used Stata version 17 to perform the estimation. We suppressed the constant term for the whole model by using the `noc` option in the `mixed`

command, as we were interested in the constant of each land use equation, which we obtained by adding a column vector of 1 in the vector of determinants arranged as a block diagonal matrix. We also suppressed the random intercept for the land use group by using the `noc` option after the `$k$` level of the model specification, as it was captured by the estimated constant for each land use equation. We obtained the estimates of the fixed effects coefficients, the random effects variances, and the error covariance matrix from the output of the `mixed` command. Ω was estimated by specifying an unstructured covariance matrix for the error terms using `residuals (unstructured)`option in the `mixed` command to allow us account for the possible correlation between the errors of different land use equations. We also performed post-estimation tests and diagnostics to check the validity and accuracy of our model.

C. ***Diagnostic detection techniques***

Our change detection analysis began with visual observation (LULC map and graphs) of land cover of the two periods (1986 and 2016). Spatiotemporal land cover distribution for each LHZ and Tigray as whole were analyzed in terms of percentages of total area of each landscape in each period to visually identify the changes that are associated with LULCC in each landscape. Moreover, a growth rate-based change detection diagnosis was also adopted to quantify LULC change relative to the reference period, 1986. Average annual rate of LULC change between study periods was calculated as:

$$R_c = \frac{1}{(t_2 - t_1)} * ln\left(\frac{A_2}{A_1}\right) * 100\% \qquad \text{(C-9)}$$

Where R_c is an average annual rate of change (%); A_1 is amount of land cover (hectare) in time 1986; A_2 is an amount of land cover (hectare) in time 2016; and t_2 and t_1 are the time two study years.

Transition matrix method

Transition matrix is a technique to compare LULC of a landscape at two different time periods, providing comprehensive and detailed "from-to" LULC change information without requiring data normalization between the two dates (Table 6). It organizes categories of the initial time at the rows and categories of the subsequent period at the columns. It displays the proportion of the observed landscape that do not experience any change (P_{jj}) on the diagonal of the matrix, whereas proportions of the observed landscape that experience change at the off diagonals. Entries off the diagonal indicate a transition from category i to a different category j. The column sum of each category during the first period (P_{i+}) denotes the proportion of the landscape in category *i* in the initial time while the row total of each category during the subsequent time (P_{+j}) denotes the proportion of landscape in category *j* in the subsequent time.

Table 6. Transition matrix for comparing two maps in different time periods.

	Subsequent time				Initial time total
	Category 1	Category 2	…	Category *j*	
Initial time					
Category 1	P_{11}	P_{12}		P_{1j}	P_{1+}
Category 2	P_{21}	P_{22}		P_{2j}	P_{2+}
…	…	…	…	…	…
Category *i*	P_{i1}	P_{i2}		$P_{jj;\ i=j}$	P_{i+}
Subsequent time total	P_{+1}	P_{+2}		P_{+j}	1

Using the most general information on the matrix, we began quantifying the changes associated with LULC changes for each category using net change (in absolute value) given as:

$$N_j = |P_{+j} - P_{i+}| \quad \text{(C-10)}$$

Though such a net change in the quantity of a category indicates a definite change on the landscape, a lack of net change (N_j=0) does not necessarily indicate a lack of change on the landscape since the net change fails to capture the swapping component of

change. Swamp is component of change when a given quantity (hectare) of a category loss at one location is accompanied by the same quantity (hectare) of the category gain at another location. To fill such shortfalls, therefore, we adopted a methodology introduced by (Pontius Jr et al., 2004) that accounts persistence (proportions at the diagonal) in examining landscape change. This method assumes persistence dominates most landscapes (as evidenced in many studies) and transitions need to be analyzed relative to it. With such relative quantification, we decomposed transitions to its various components to identify signals of systematic land change.

Component of change analysis

A more precise way of detecting LULC dynamics is to quantify landscape transition relative to persistence. Gross losses and gains, net change, and swap are important components of total change in the analysis of landscape dynamics considering signals of persistence on the landscape. To arrive at each component, we first calculated gross loss (L_i) and gross gain (G_j) as follows:

$$L_i = \left|P_{i+} - P_{jj}\right| \text{ and } G_j = \left|P_{+j} - P_{jj}\right| \qquad \text{(C-11)}$$

L_i is proportion of landscape that experiences gross loss of category *i* during the study period and G_j is proportion of landscape that experiences gross gain of category *j* during the study period.

The second important component, swamp (S_j), defines the swap location change that occurs when the landscape demonstrates simultaneous gross gain and gross loss (such as reforestation in some portion of the landscape and deforestation in other part). This component is constructed by pairing each grid cell that gains with grid cell that losses to create a pair of grid cells that swap for each category. When amount of gain is equal to

the amount of loss ($N_j = 0$), then the total change is all the swamp. Hence, the total change is twice the gain (or loss). If not, the amount of swamp (S_j) was calculated as:

$$S_j = 2 * Min(L_i, G_j) \tag{C-12}$$

S_j does not capture portion of the change in excess of gain(loss) when gains are not equal to losses. The net change captures remaining unpaired gain or loss after all gains and losses have been paired to compute the amount of swap. Hence, N_j, defined in Eq (2) above can be redefined as:

$$N_j = Max(L_i, G_j) - Min(L_i, G_j) = |P_{+j} - P_{i+}| \tag{C-13}$$

Having the swamp and the net change components, we calculated total change for each category as the sum of the swamp change and net change as:

$$T_c = S_j + N_j = Max(L_i, G_j) + Min(L_i, G_j) \tag{C-14}$$

Vulnerability to LULCC analysis

Once we analyzed each component of change (transition), indices of persistence were calculated to show vulnerability of each category to LULC dynamics as follows:

$$G_P = G_j / P_j$$

$$L_P = L_j / P_j \tag{C-15}$$

$$N_P = N_j / P_j$$

Where G_P is gross gain to persistence ratio, L_P is gross loss to persistence ratio, N_P is net change to persistence ratio and P_j is proportion of the landscape of a category j that did not exhibit any change (transition).

Land use land cover categories with relatively higher $G_P$were asserted to have a higher tendency to gain surface instead of remaining stable, whereas land use land cover categories with relatively higher L_P were declared to be highly vulnerable to loss surface (loss surface instead of remaining stable). N_P shows magnitude of net surface loss/gain relative to persistence.

On the other hand, based on such relative (to persistence) quantifications, there may be misleading decisions showing lower tendencies to change of a LULC category while undertaking the greatest loss/gain and the highest total change. Such inconsistency in signaling change can be explained by size of LULC categories and their persistence levels. A larger transition observed in a category in the transition matrix may not necessary indicate relatively higher LULC change in the category as a category that account for large portions of the landscapes may show higher transition due its share, hence, not systematic transition.

Pontius et. al. (2004) noted even through a random process, largest LULC categories may experience sizable transitions (changes) among themselves. Such wrong signals of transition along with the spurious landscape dynamics due to larger persistence size relative to changes (Alo & Pontius Jr, 2008) leads to the adoption of identification of key and systematic patterns of change separate from any persistence level and land cover size (Briones & Varas, 2016).

Detecting key signals of LULC change

The analysis of persistence, gains and losses is instructive, but it fails to identify whether transitions were random or systematic as it fails to examine the dynamics among the off-

diagonal entries. Hence, we followed Pontius et. al. (2004) methodology to analyze the off-diagonal entries to identify systematic transitions of land change for a given landscape's degree of persistence. In identifying systematic transitions within the transition matrix, we constructed two expected transition matrices based on losses and gains that are expected to occur due to random process. In the expected transition matrix based on gross again, the gross gain of any one category is distributed based on the distribution of the other categories in the initial time (1986) as Eq (8) assuming that the total gross gain of each category is fixed:

$$G_{ij} = \left(P_{+j} - P_{jj}\right) * \left(\frac{P_{i+}}{\sum_{i=1,i\neq j}^{J} P_{i+}}\right) \quad \text{(C-16)}$$

Whereas, in the expected transition matrix based gross loss, the gross loss of any one category is distributed based on the distribution of the other categories in the subsequent time (2016) as Eq (9) assuming that the total gross loss of each category is fixed:

$$L_{ij} = \left(P_{i+} - P_{jj}\right) * \left(\frac{P_{+j}}{\sum_{i=1,i\neq j}^{J} P_{+j}}\right) \quad \text{(C-17)}$$

Where G_{ij} is the expected transition from category *i* to category *j* due to a random process of gain, L_{ij} is the expected transition from category *i* to category *j* due to a random process of loss, $(P_{+j} - P_{jj})$ is the observed gross gain of category *j*, $(P_{i+} - P_{jj})$ is the observed gross loss of category *i*, P_{i+} is the size of category *i* in the initial time, P_{+j} is the size of category *j* in the subsequent time, and the notation $i \neq j$ in the summation of each equations excludes size of the category under consideration from the sum of the sizes.

Eq (8) rearranges only the off-diagonal transitions within each column while holding persistence constant and Eq (9) rearranges only the off-diagonal transitions within each row while holding the persistence constant on the diagonal.

A transition is said to be systematic if the difference between transition observed in observed transition matrix deviates from transition expected in expected transition matrix. To provide conclusive evidence of key signal of change, category *i* should systematically loss to category j whilst category *j* is systematically gaining from category *i*. i.e., if the differences between observed and expected transitions due random losses and the differences between observed and expected transitions due random gains are significantly positive (Alo & Pontius Jr, 2008).

Table 7. Equation by Equation regressions (OLS of each land use equations)

Land use share equation		Cropland	Pasture	Fallow	Forest	Others
Plot level factors	Slope (%)	0.0010	(0.0005)	0.0009	0.0005	(0.0005)
		(0.0007)	(0.0008)	(0.0012)	(0.0010)	(0.0008)
	Elevation (m)	0.0000	0.0000	0.0001	0.0000	0.0000
		0.0000	(0.0000) *	0.0000	0.0000	0.0000
	Dist. to residence (kms)	0.0003	(0.0027)	0.0020	(0.0059)	(0.0060)
		(0.0007)	(0.0089)	(0.0058)	(0.0088)	(0.0031) *
	Soil type is vertosol (Yes=1)	(0.0147)	0.0087	0.0889	(0.0500)	(0.0274)
		(0.0138)	(0.0222)	(0.0326) ***	(0.0251) *	(0.0194)
	Soil quality: Fair	(0.0208)	0.0150	(0.0088)		(0.0816)
		(0.0134)	(0.0152)	(0.0315)		(0.0186) ***
	Soil quality: Poor	(0.0280)	(0.0105)	0.0255		(0.0986)
		(0.0152) *	(0.0162)	(0.0351)		(0.0208) ***
	Land tenure: Tenanted	0.1047	(0.0361)	(0.0840)		0.2923
		(0.0315) ***	(0.0594)	(0.0826)		(0.0356) ***
	Land tenure: Sharecropped	0.0200	0.0022	(0.0576)		0.0493
		(0.0289)	(0.0452)	(0.0727)		(0.0407)
Household level factors	Literate house head (Yes=1)	(0.0244)	(0.0120)			0.0149
		(0.0120) **	(0.0147)			(0.0169)
	Dependency ratio	(0.0160)	0.0033	0.0290		(0.0149)
		(0.0059) ***	(0.0064)	(0.0123) **		(0.0082) *
	Ln of total area (hectare)	0.0656	0.0659	0.1073	0.0433	0.0384
		(0.0046) ***	(0.0051) ***	(0.0106) ***	(0.0087) ***	(0.0088) ***
	No of plots owned	(0.0056)	(0.0057)	(0.0167)	(0.0056)	(0.0285)
		(0.0014) ***	(0.0014) ***	(0.0024) ***	(0.0022) **	(0.0016) ***
	Livestock (TLU)		(0.0040)			
			(0.0018) **			
	Asset index	(0.0901)		(0.0391)		0.1569
		(0.0487) *		(0.1400)		(0.0645) **
	Ln of annual income	0.0032			(0.0138)	
		(0.0035)			(0.0072) *	
	Dist. to nearest market (km)	(0.0005)	(0.0005)	(0.0025)	(0.0021)	0.0005
		(0.0003) *	(0.0004)	(0.0008) ***	(0.0028)	(0.0004)
	Dist. to Major Road (km)	0.0006	0.0008	0.0012	(0.0023)	(0.0019)
		(0.0004)	(0.0007)	(0.0011)	(0.0037)	(0.0006) ***
	Climate change perception index	(0.0291)	0.0000		(0.0332)	0.0113
		(0.0157) *	(0.0001)		(0.0347)	(0.0219)
Community level factors	Average output price (Birr)	0.0000	(0.0004)		0.0002	
		0.0000	(0.0003)		(0.0001) ***	
	Population density (per km^2)	0.0000	0.0000	0.0000	0.0000	0.0000
		0.0000	0.0000	0.0000	0.0000	(0.0000) ***
	Annual mean Rainfall (mm)		0.0000		(0.0002)	
			(0.0001)		(0.0005)	
	Prop. of agri. in 1 km buffer				(0.0547)	
					(0.1092)	
	Livelihood Zone: ALL	(0.0215)	(0.0231)	0.0044		(0.0860)
		(0.0240)	(0.0348)	(0.0538)		(0.0334) **
	Livelihood Zone: CMC	0.0351	0.0197	(0.0640)	(0.2027)	(0.0496)
		(0.0208) *	(0.0294)	(0.0589)	(0.1673)	(0.0301) *
	Livelihood Zone: EDM	0.0245	0.0451	(0.0870)	(0.0693)	(0.0767)
		(0.0237)	(0.0606)	(0.0874)	(0.1219)	(0.0330) **
	Livelihood Zone: EPL	(0.0562)	(0.0278)	(0.0552)	(0.1341)	0.0950
		(0.0228) **	(0.0225)	(0.0503)	(0.1144)	(0.0300) ***
	Livelihood Zone: HSS	(0.0452)	0.0073	0.0355		(0.0866)
		(0.0272) *	(0.0409)	(0.0629)		(0.0366) **
	Livelihood Zone: WCT	(0.0050)	(0.0175)	0.0557	(0.0684)	0.0606
		(0.0235)	(0.0259)	(0.0541)	(0.1007)	(0.0324) *
	Constant	0.9945	0.3240	0.5882	0.7709	0.5448
		(0.0555) ***	(0.1042) ***	(1215) ***	(0.3408) **	(0.0736) ***
	R^2	0.3200	0.7300	0.6000	0.7100	0.4300
	N	916	123	196	57	919
	F	16.64 ***	11.11 ***	12.26 ***	5.22 ***	29.71 ***
	AIC	-803.2686	-330.8000	-159.5978	-144.0090	-178.2386

Notes:

[1] A farming household is a household that engages in mixed farming, mainly involves both crop and livestock production on the same farm. A household is composed of one or more persons who live and eat together, and who have a head who oversees the household's economic and social affairs.

[2] In Tigray, farmers often receive input supports from the government, such as subsidies or free inputs, that lower their production costs. The government also intervenes in the market by setting price ceilings or floors for some inputs and outputs, that affect their revenues.

[3] Number of plots in different locations indicate level of land fragmentation. The more the number of plots in different locations, the more fragmented land (Yang et al., 2022).

[4] Elevation: High (> 1500 m), Medium (500 - 1500 m), Low (< 500 m) and Slope: Sloppy (> 15%), Moderate (5 - 15%), Flat (< 5%) (Brouwer et al., 1985).

[5] Plot, a unit of analysis in this study, is piece of land owned (and managed) by a household in all the three waves. It may consist of several fields that can have different uses. That is plot can be allocated to different uses either in its entirety or in parts (a mixed land cover type). A household may own one or more plots in various locations and each plot is assumed to have similar characteristics, such as soil quality, size, location, or access to water and inputs.

[6] For example, crop production may have higher revenue but also higher input costs and environmental degradation than pasture production. Tradeoff in land uses, then, implies there is interaction or spillover effect among outputs, such as opportunity cost, income effect, or environmental impact.

[7] The dependent variables for each kth land use are stacked into a single vector (nx1) of dependent variables.

[8] Such data structure is intentionally done by careful data manipulations (preparation) to trick Stata in to estimating a system of land use equations apparently as a single equation. This is because with time invariant plot level factors, adopting seemingly unrelated regression (SUR) was not feasible.

[9] If a plot (in part or in its entirety) enter a land use category at the first wave and switch in its entirety to other land use, it may be back to its original land use in the next wave creating gap in waves in the land use category.

[10] The logic behind adopting the method of Biorn et al. (2003) is to account for the unbalancedness of the panel data and to capture the degree of sustainability of a plot in a land use type. By grouping the plots by the number of waves they are observed in, we can compare the plots that are more stable (observed in all three waves) with those that are less stable (observed in one or two waves). This allows us to test how different factors affect the sustainability of plot in a land use. A plot (in part or its entirety) will sustain all the three-waves in a land use category if there are favorable household (like capital availability) or/and community-level features (environmental factors), if not, lesser level of sustainability (in two-waves or in a single-wave). For example, capital constraints may prevent farmers from investing in productivity-enhancing technologies and inputs, such as improved seeds, fertilizers, irrigation, etc. This leads to low yields and low returns from agriculture, which discourages farmers and make them leave some land fallow or clear more land from forests to expand their cultivation area, hoping to increase their output and income.

Figure

Click here to access/download;Figure;Figure_1.jpg

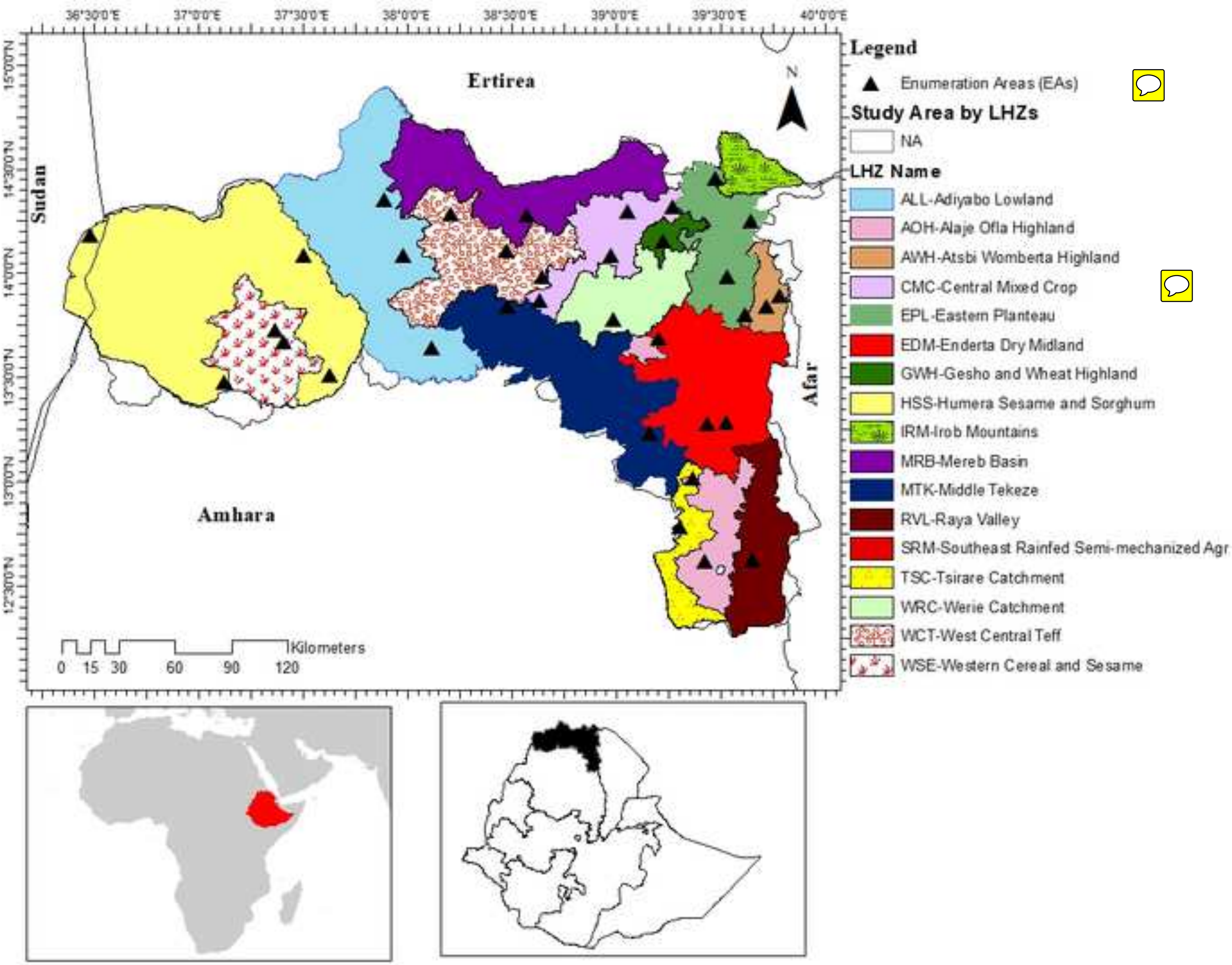

Figure

Click here to access/download;Figure;Figure_2(a).jpg

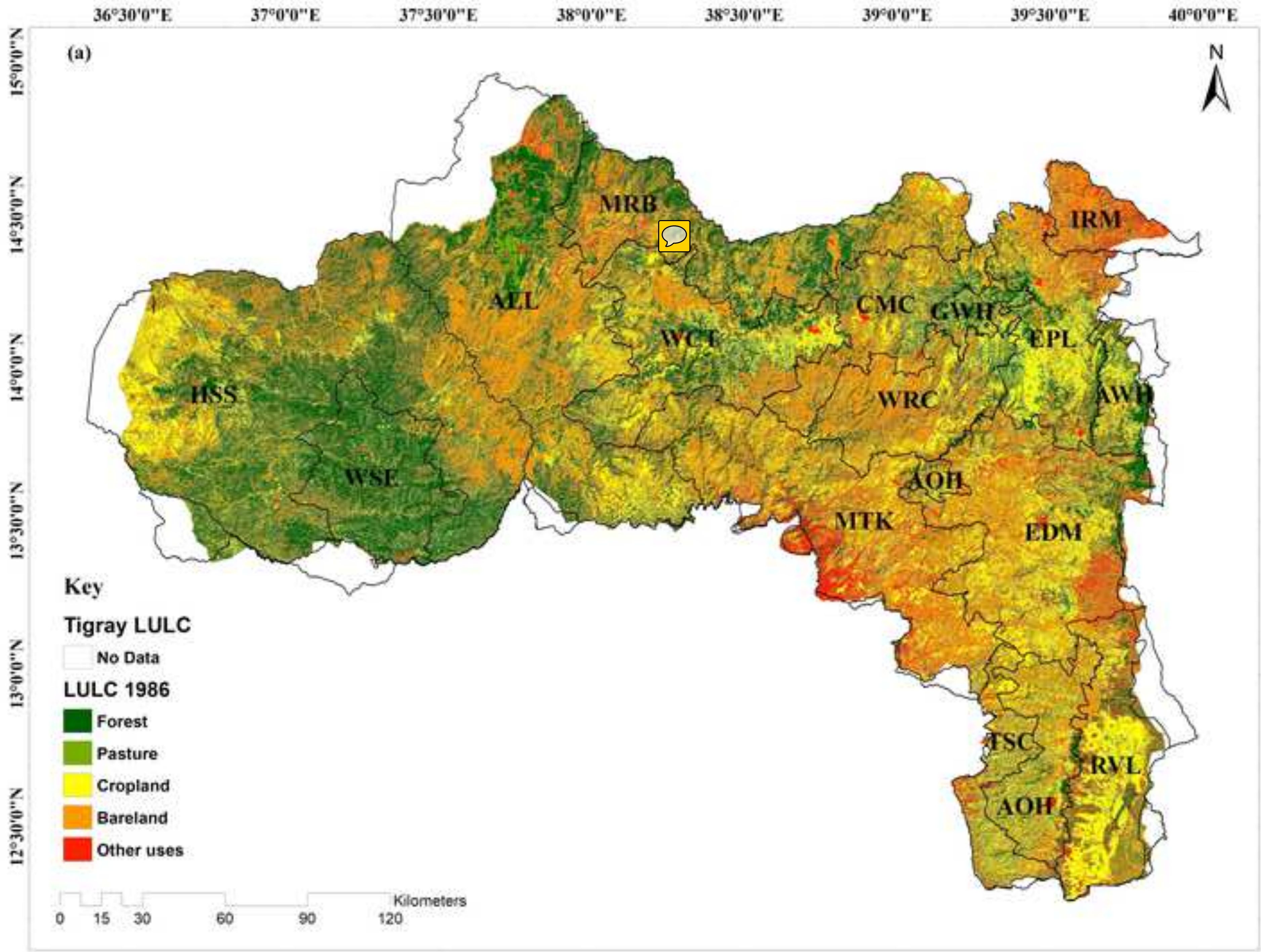

Figure

Click here to access/download;Figure;Figure_2(b).jpg

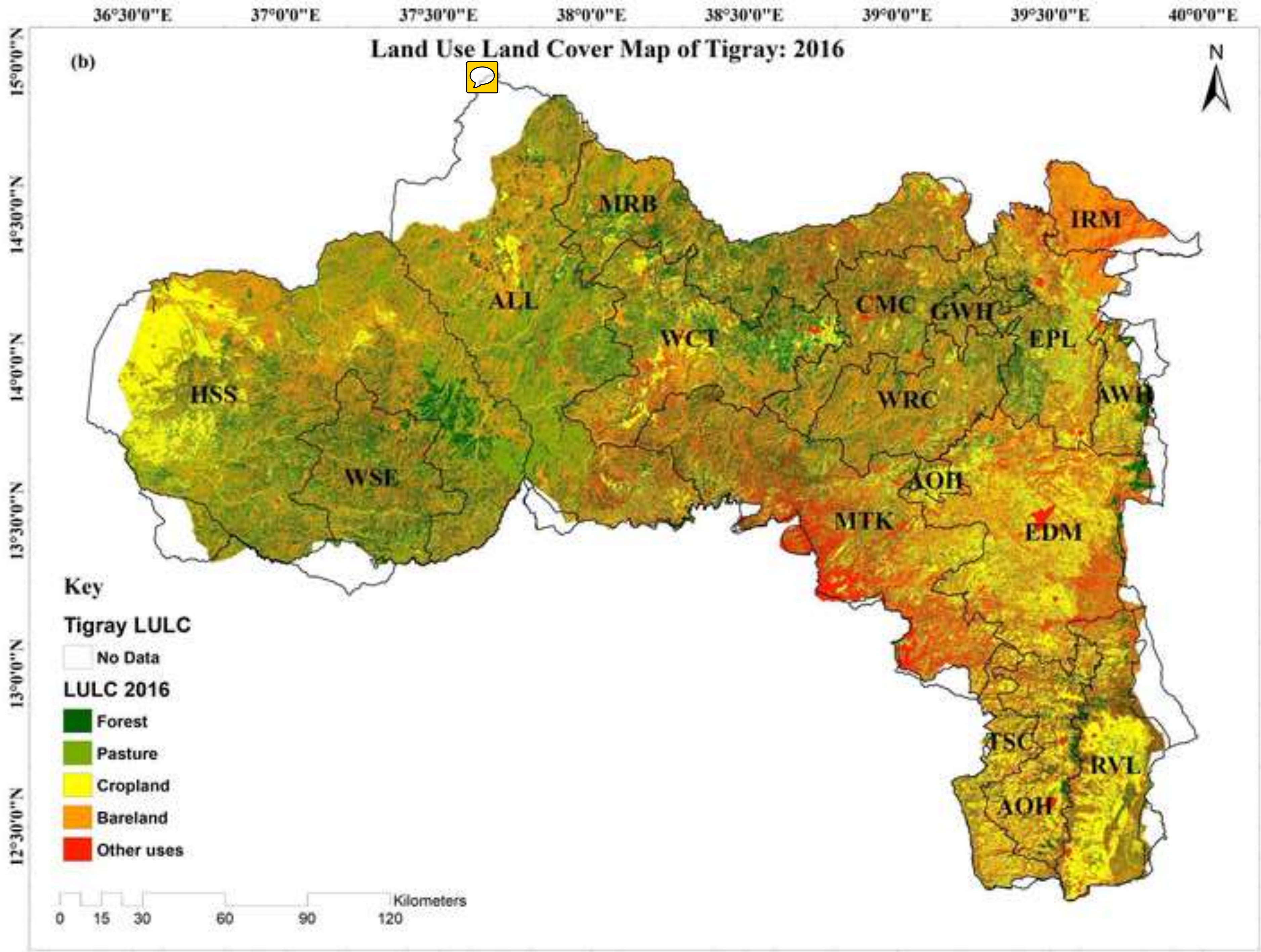

Figure

Click here to access/download;Figure;Figure_2(c).jpeg

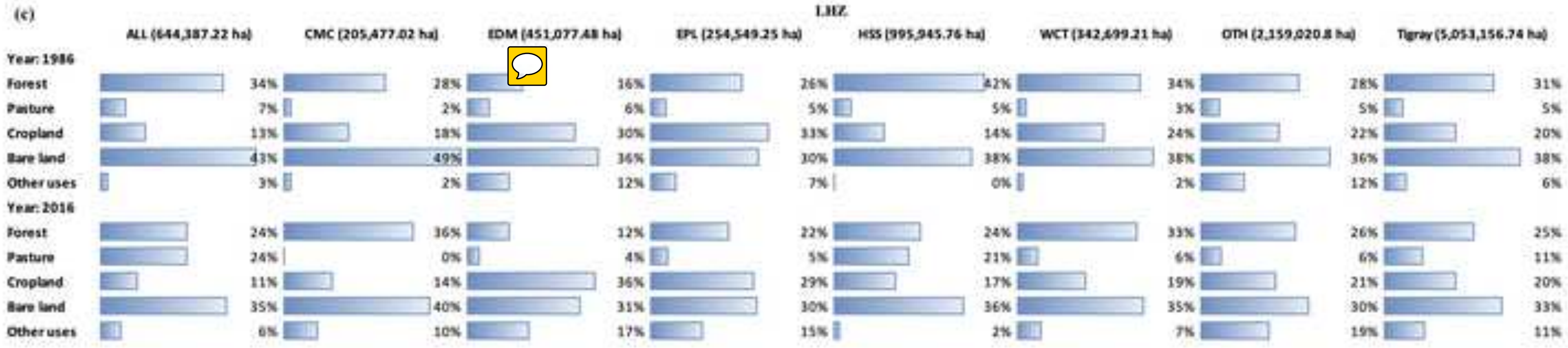

Figure

Click here to access/download;Figure;Figure_3.jpeg

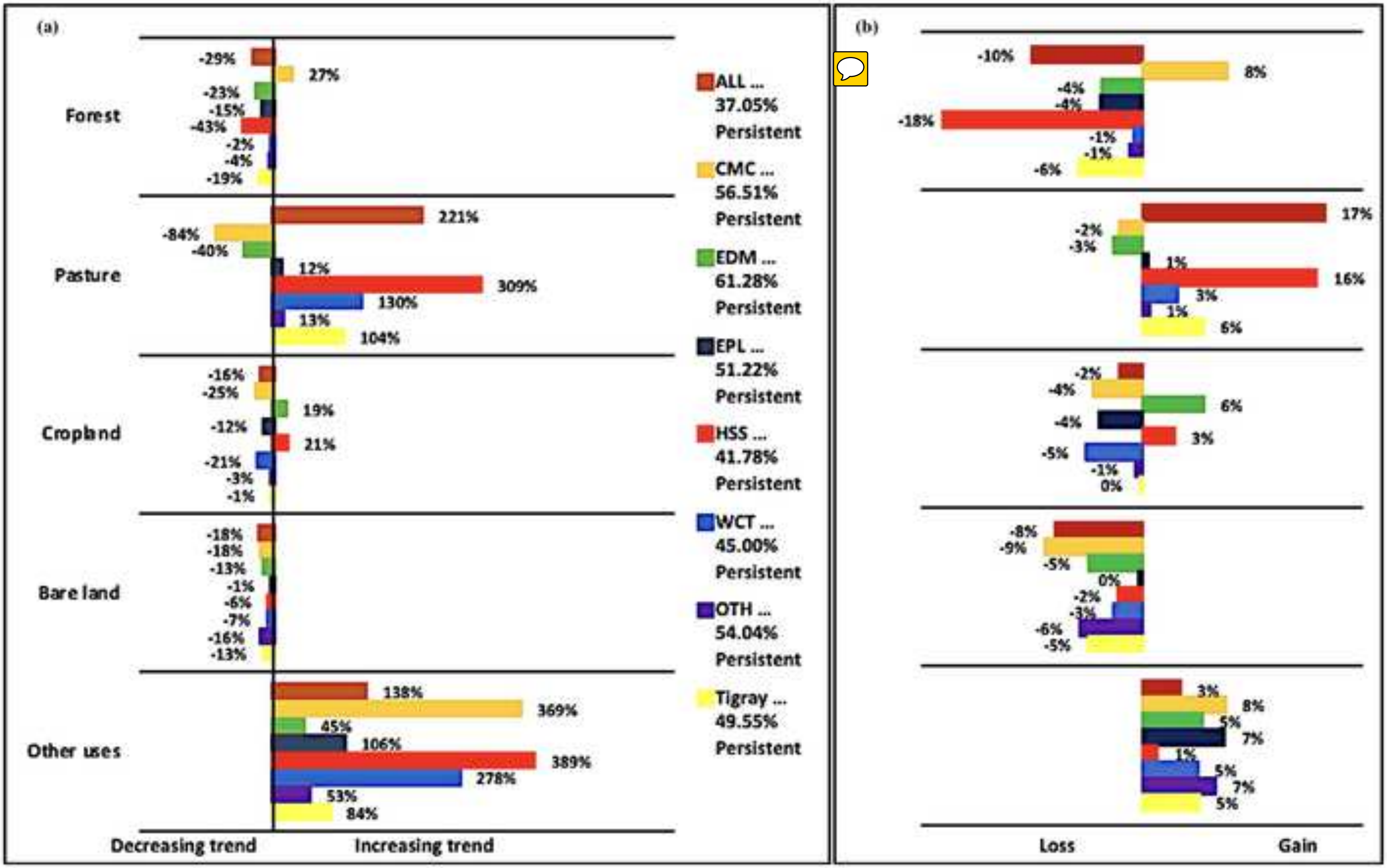

Figure

Click here to access/download;Figure;Figure_4.jpeg

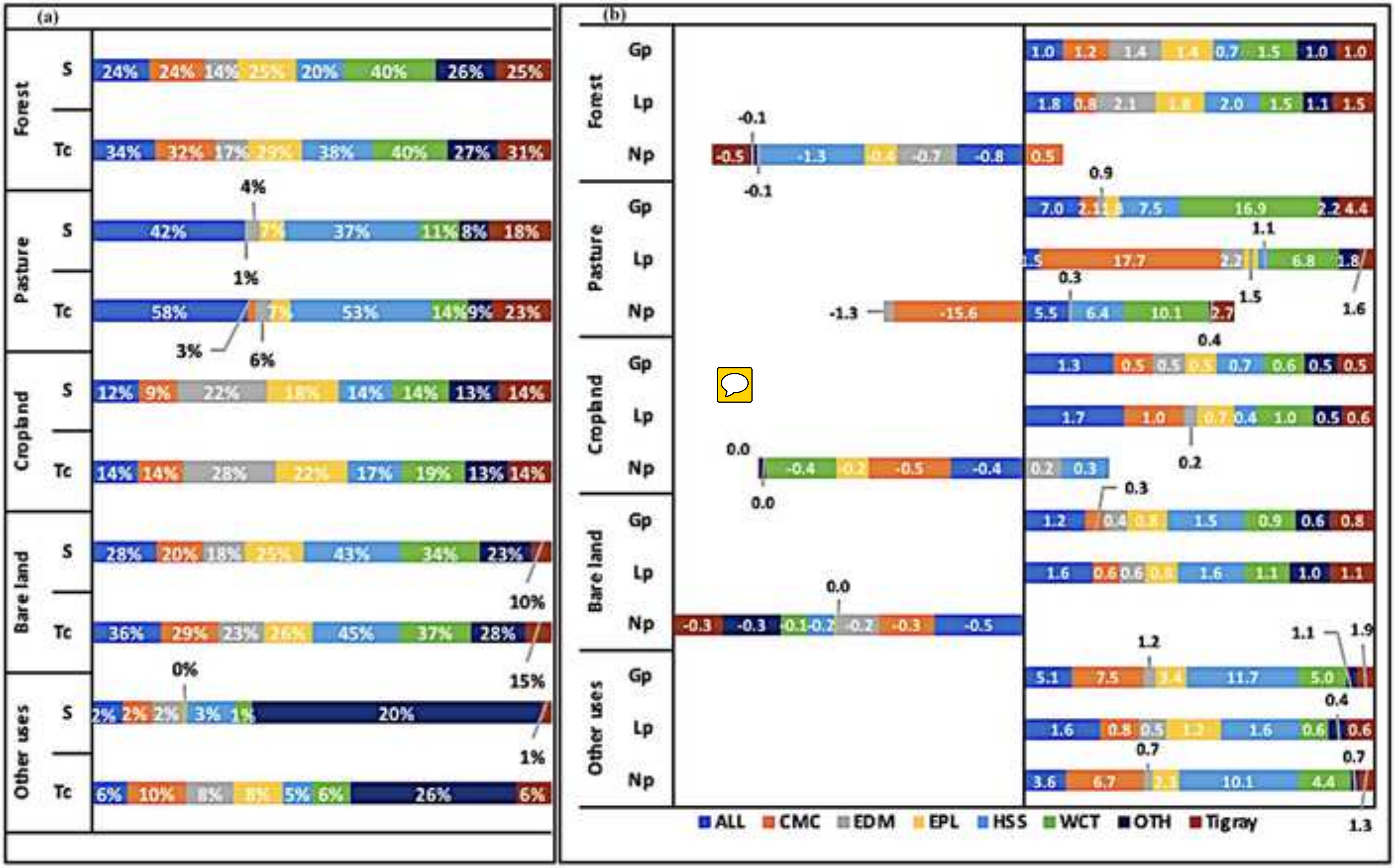

Figure
Click here to access/download;Figure;Figure_5.jpeg

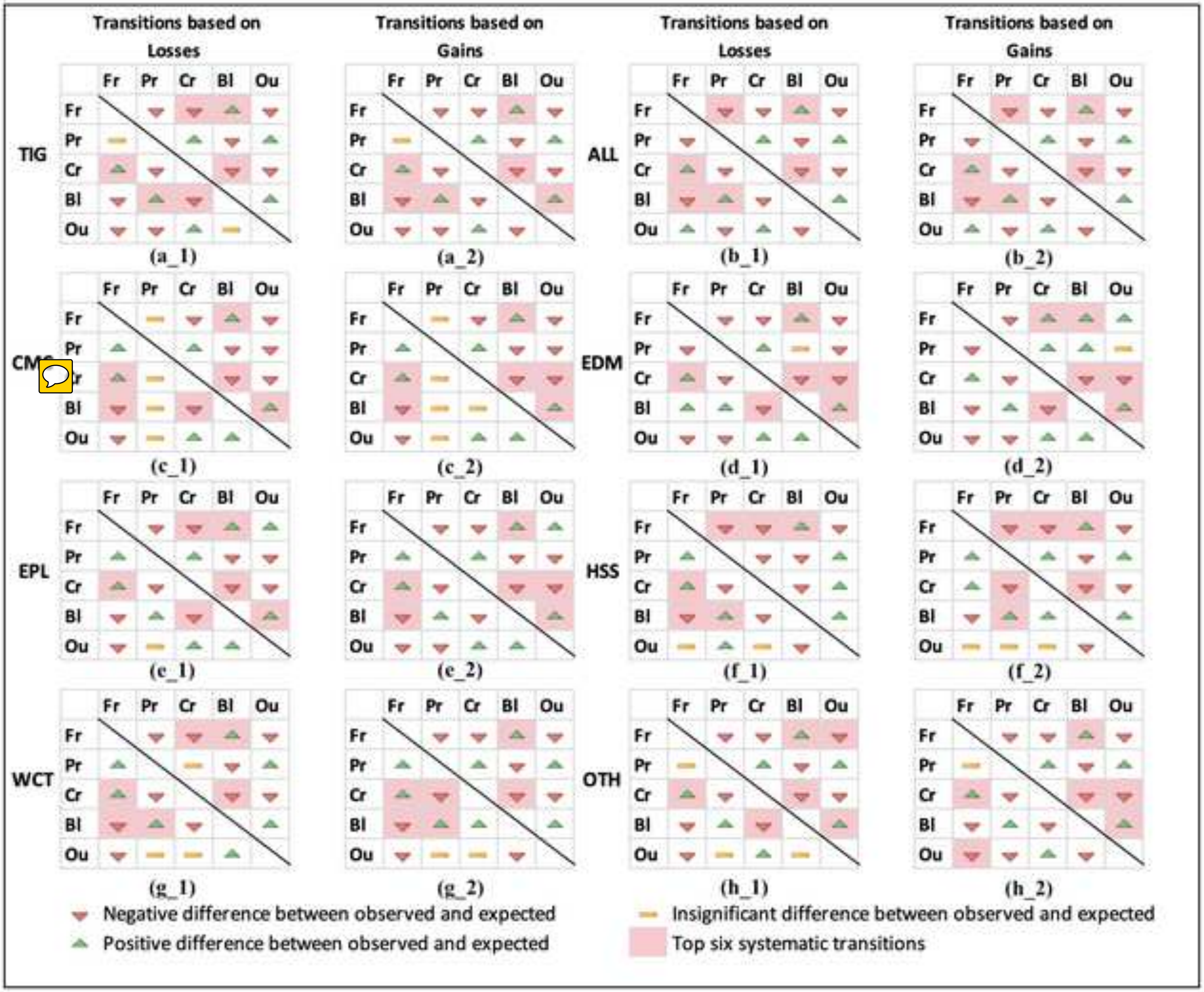

Supplement: Attachment — Submitted filename: PONE-D-23-33020_reviewer.pdf [file pone.0304896.s011.pdf]
