# Supplementary material for: Multi-level determinants of land use land cover change in Tigray, Ethiopia: A mixed-effects approach using socioeconomic panel and satellite data
Source: PLoS One. 2024 Jun 13;19(6):e0304896. doi: 10.1371/journal.pone.0304896 (PMC11175475; doi:10.1371/journal.pone.0304896)
Supplement: Attachment — Submitted filename: Response to Reviewers.docx [file pone.0304896.s012.docx]

Dear Dr. Josily Samuel,

We are grateful for your and reviewers’ constructive feedback on our manuscript entitled “Multi-level determinants of land use land cover change in Tigray, Ethiopia: A mixed-effects approach using socioeconomic panel and satellite data” (manuscript number PONE-D-23-33020), submitted to PLOS ONE.

We appreciate your consideration of our work for revision. We have revised the manuscript according to your comments and addressed all your concerns.

This letter explains how we have revised our manuscript in response to each of your comments. We hope that our revisions meet your expectations, and our manuscript is now ready for publication in PLOS ONE.

We have prepared a point-by-point response to each issue raised by you and the reviewers. We have also listed the changes that we have made in the revised manuscript, which are marked in blue. For the comments that only appear in the manuscript (mainly related to reference, definition of terms, acronym style, grammar, or formatting), we have summarized them in annexed Table 1 that includes the reviewer comment, the change made, and the location in the revised manuscript and justification (if needed). We do not categorize the comments to reviewer #1 and #2, as there is no clear indicator whose comments are in the manuscript. We apologize for this.

Please find below our detailed responses to you and the reviewers, followed by the list of changes that we have made in the revised manuscript.

Sincerely,

Tadele Tafese Habte

Centre for Environment and Development, College of Development Studies, Addis Ababa University, Addis Ababa, Ethiopia

**Response to Reviewer #1**

Reviewer comment 1

- Line 79: I am not as such convinced why religion was described as it is not relevant to this particular topic

Author response 1

- *Thank you for your suggestion. We agree that religion is not relevant to our study on land use choice and land cover change. We have removed the sentence about the respondents’ religious affiliation from the background section, paragraph 1.*

Reviewer comment 2

- Line 89-94: is out of the context of this study and hence remove

Author(s) response 2

- *Thank you for your feedback. We acknowledge that the paragraph was not relevant to our study on land use/land cover change, as it did not match the temporal scope or the variables of our analysis. Therefore, we have removed the paragraph from the background section.*

Reviewer comment 3

- The 17 livelihood zones and enumeration areas should have been briefly described for readers and supported with relevant literature. Please provide some background information with regard to these two important concepts indicated.

Author(s) response 3

- *We think this is good suggestion. We have added a short description of the enumeration areas (EA) and the livelihood zones (LHZ) in lines 83-96. The LHZ is a spatial classification based on the dominant livelihood activities and agro-ecological conditions, while the EA is the primary sampling (smallest geographic) unit, used by the Central Statistics Agency (CSA) of Ethiopia for survey sampling and data availability. We have cited the relevant sources for these definitions.*

Reviewer comment 4

- Line 98: change "farms" in to " farmers" which It seems that farms are not engaged.

Author(s) response 4

- *Thank you for your correction. We have changed the word “farms” to “farmers” in line 99.*

Reviewer comment 5

- Line 106-109: Do not you think that soil and water conservation initiatives are one of the components of PSNP? Please try to show the difference if it is actually different? For me it is part of the SLM programs initiated through PSNP.

Author(s) response 5

- *Thank you for your comment. We agree that SWC is one of the SLM programs implemented through PSNP. However, we also want to highlight the SWC campaign that the government of Tigray has launched to restore degraded lands, which involves the participation of all farmers regardless of their PSNP status. We have added a sentence to clarify this difference in lines 108-109.*

Reviewer comment 6

- Line 126-127: when was the survey conducted? Did you get the same households? Please clarify

Author(s) response 6

- *Thank you for your inquiry. We have added more details on the follow-up survey that we conducted to supplement the ERSS, which is part of the LSMS-ISA project by the World Bank. This survey reached out to all the 387 households who participated in the 3^rd^ round of ERSS/ LSMS-ISA in August 2019. We have mentioned this information in lines 137-141.*

Reviewer comment 7

- Line 152: How many key informant interviewees were used and why?

Author(s) response 7

- *To validate the old LULC maps, we interviewed 34 key informants (one from each EA) who were elderly people. The number of interviews was limited by the resource and access constraints, as we conducted them along with the ERSS/LSMS-ISA follow-up survey. This is indicated in lines 175-176.*

Reviewer comment 8

- Line 210-215: repeated

Author(s) response 8

- *Thank you for your observation. We have removed the duplicate paragraph from page 12, under empirical model and estimation.*

Reviewer comment 9

- The discussion section must be organized in to sub heading similar to the results section

Author(s) response 9

- *Thank you for your suggestion. We have followed your recommendation and divided the discussion section into three subsections: (1) land use choice analysis, (2) land cover change, and (3) policy implications, matching the results section. Introduced subsection headings are indicated in lines 425, 464 and 500, respectively.*

**Response to Reviewer #2**

Reviewer comment 1

- Introduction: The Introduction is too short. There should be more content related to similar work of LULCC in the Tigray Region. There are already quite a few publications about LULCC in the Tigray Region. You have tried to provide your arguments (what distinguishes your research from others) but this could be better filled with content. I understand that your manuscript is already quite long but it might be better to shift other content to the annex and rather improve the introduction.

Author(s) response 1

- *Thank you for your constructive feedback. We understand your concern about the introduction section being too short and lacking sufficient content on LULC change in Tigray. We have revised the introduction section by adding more references on LULC change in Tigray and highlighting the research gaps that our study addresses. We have also moved some content to the annex to shorten the manuscript. Please see lines 57-66 for the revised introduction section.*

Reviewer comment 2

- Background: The map needs to be improved (see my comments in the manuscript).

Author(s) response 2

- *Thank you for your feedback on the map of the study area. You wanted us to define the enumeration area (EA) and the land use (LU) types, and to show the LU types on the map. We have updated our manuscript to explain the EA and the livelihood zones (LHZ) on the map. The EA is the smallest geographic unit used by the central statistics agency (CSA) of Ethiopia for survey sampling and data availability. The LHZ is a spatial classification based on the dominant livelihood activities and agro-ecological conditions. We have added these definitions to the caption of Fig. 1 in lines 83-87 and to the introduction section in lines 88-96. LU types in this study are the main land purposes of plots (cropland, forest, pasture, fallow and other uses) as defined in the Ethiopia Rural Socioeconomic Survey/* *World Bank’s Living Standards Measurement Study, Integrated Survey on Agriculture (ERSS/LSMS-ISA) dataset. We do not show plot level LU types on the map due to confidentiality terms of using the ERSS/LSMS-ISA. We have defined LU types in lines 229-230. We have removed the map of land cover types, as requested by the editor, due to some copyright issues. We apologize for this inconvenience and thank the editor for noticing it. We hope that this answers your questions and that you are happy with our revisions.*

Reviewer comment 3

- Method: There are existing sources of data that were used (ERSS and LSMS-ISA) but there is not much explanation. Please provide more details about the identification of the representative sample of households, collected variables and their definition, e.g., survey template, reason of data collection, time of the data collection, etc. The research question in “Method of data analysis” does not fit to the objective from the introduction. I am confused what is actually the aim of this study.

Author(s) response 3

- *Thank you for your feedback. We have revised the sub-section on data source and method of data analysis (lines 115-141) to provide more information about the socioeconomic data, both ERSS and its follow-up survey, which captured the public perceptions of climate and LULC change. We have explained the following aspects of the surveys: (1) their aim and scope, (2) their sampling strategy and size, (3) their main variables and definitions (or survey templates in S4_Appendix), (4) their timing and rationale, and (5) their availability and access. We are sorry for the inconsistency between the objective in the method of data analysis and in the introduction. This was a mistake that we made while editing the manuscript. We have fixed it in line 190, to match the objective in the introduction. We hope that this clarifies your concerns and that you are satisfied with our revisions.*

Reviewer comment 4

- Only two years of spatial data were compared (1986 and 2016). The spatial classification of land use types is unclear and needs more specification that is backed-up with references. In addition, no data were available for the year 1986 from the Rural Socioeconomic Survey (only 2012, 2014, and 2016). How this data gap was addressed?

Author(s) response 4

- *Thank you for your feedback. We have revised the manuscript to address the issues you raised about the spatial data and the land use classification. We admit that using only two years of spatial data (1986 and 2016) and having no data for 1986 from the ERSS may affect the validity of our analysis. We justify our choices and solutions as follows: First, we encountered two challenges when analyzing LULC change with the panel data from ERSS/LSMS-ISA for 2012, 2014, and 2016. The panel data enabled us to study land use allocation with a mixed-effects model, which explained the recent changes in land use well. However, the panel data had a low temporal resolution (5 years and 3-time steps), which limited our ability to capture the trends and patterns of land use transitions. Therefore, we used spatial data from satellite imagery (LandSat) for 1986 and 2016 for the whole region, to detect more reliable and noticeable land cover change. This created a temporal and spatial mismatch with the land use allocation analysis. We addressed this by combining the long-term land cover change analysis with the short-term land use allocation analysis, while keeping our objective (to examine the determinants of land use choices and land cover dynamics in Tigray). We acknowledged the limitation of this approach in lines 249-258. Second, we selected 1986 and 2016 as the two years of spatial data for these reasons (lines 147-150). We matched 2016 with the year of the panel data (2012-2016). We chose 1986 because it was a drought year in Tigray, Ethiopia, which contrasted with the current situation. Another reason was the difficulty of satellite image processing for such a large area. We wanted to add more years in between, but the war in Tigray in 2020 and the communication blackout stopped us from doing so. We decided to continue with our analysis with the data we had for about 3 years of crisis. Third, we explained the spatial classification of land use types in depth and added more references to support it in lines 143-188. We had land use classification from the panel data at the plot level as cropland, forest, pasture, fallow, and other uses, but the classification from satellite imagery processing was: cropland, grassland/pasture, forest, bushland, bare land, and others. To harmonize the classifications, we used information from the key informant interviews (KII) during the ERSS/LSMS-ISA follow-up survey. The informants said that Natural and plantation forests found in the study area include woodlands, shrubs and bushlands that are under government and communal protection and agroforestry areas. We agreed with the respondents’ definition of forest and included bushland and shrubs in it.*

Reviewer comment 5

- Results: Figure 2c, 3 and 4 are difficult to read and hard to understand. This rather causes confusion. Actually, I stopped reading until the end of the results´ section because it is hard to understand.

Author(s) response 5

- *Thank you for your feedback. We are sorry for the confusion caused by the graphs of land cover change analysis in the results section. We have made the following changes to improve our presentation: (i) We focused on the change detection based on trend and transition matrix, as suggested by the reviewer, instead of change detection based on change in land cover share. We also deleted the land cover maps, as requested by the editor, due to some copyright issues. (ii) We separated the trend change in land cover (previously Fig. 3a) from the net change (previously Fig. 3b) and made a standalone figure (Fig. 2). We combine net change in land cover (Previously Fig. 3b) and swamp and total change in land cover (Previously Fig. 4a) and made them a standalone figure (Fig. 3). Fig. 2 and Fig. 3 show consistent patterns of land cover change, while Fig. 3 also shows additional information components of change (net, swap, and total changes). (iii) We replaced the persistence indices of each land cover type (previously Fig. 4b) with a table (Table 5) to make it easier to compare the values. You can see the changes made in (i) and (ii) in the new Fig. 2 and Fig. 3 and their captions in lines 342-346 and lines 357-362, respectively. The change made in (iii) is indicated in lines 378-380. (iv) We also rounded the values reported on figures to one decimal place. This may cause some minor changes in numbers. We also rewrite sentences in the results section to match the new presentation format (lines 340-341 and line 350-356). We hope that these changes will make our results clearer and easier to understand.*

Reviewer comment 6

- The methodological discussion is missing. The most recent spatial data is from 2016 - it means, it is 7 years ago and might not reflect current land use changes (current realities). I recommend to use updated spatial information.

Author(s) response 6

- *Thank you for your constructive feedback. We have updated our manuscript to include more methodological discussion in the discussion section. We have added four new paragraphs that cover the following points: (i) The model and the method we used to analyze land use allocation and land cover change, and the data we used for this purpose (lines 418-424). (ii) A short description of the mixed effects model (its assumptions) and the ERSS/LSMA-ISA data we used to fit the model (lines 426-430), and the model’s limitation in capturing long term land use change trends and patterns (lines 460-463). (iii) A short explanation of the approach we followed to detect land cover change, the data we used, and the challenges and limitations of using satellite data, such as spatial resolution and temporal frequency mismatches, limited ability to capture land cover heterogeneity and diversity within pixels, and intermediate land cover changes (lines 465-476). (iv) We added relevant literature citations to support each paragraph. We understand your concern about the timeliness of the spatial data. We selected the 2016 data because it matched the last year of the ERSS/LSMA-ISA panel data, which was the main source of information for the land use allocation determinants. However, we recognize that the ERSS/LSMA-ISA for 2018/19 is a new panel and is not a continuation of the previous ESS waves (*[*Ethiopia - Socioeconomic Survey 2018-2019 (worldbank.org)*](https://microdata.worldbank.org/index.php/catalog/3823)*). Moreover, we had difficulties in getting and validating satellite imagery data for other years due to the large area coverage and security issues in the region. Therefore, we acknowledge that there might be some recent land use changes that are not reflected by the 2016 data (especially during the recent years of Tigray war), and we recommend that future research could use more updated and high-resolution spatial data to capture the current realities of land use and land cover dynamics in the region (lines 525-528). We hope that these revisions address your concerns.*

Reviewer comment 7

- Conclusion: The section “Policy implication” is out of place. A conclusion should not show new content. Please add this section to the discussion, give more explanation and support it with references.

Author(s) response 7

- *Thank you for your constructive feedback. We have revised our manuscript to include the policy implication section in the discussion section, as you recommended (lines 500-528). We admit that the policy implication section was out of place in the conclusion section. We followed the PLOS ONE guideline of having policy implications in the conclusion section (though not mandatory). However, we understood that this is not a usual practice in other journals, and that it might mislead the readers who look for a brief summary of our key findings and contributions in the conclusion section. Therefore, we relocated the policy implication section to the discussion section, where we could give more details and references to support it.*

Reviewer comment 8

- References are largely missing that the reader cannot judge about the quality of the work. Information is partially missing, too (especially regarding the spatial land use classification approach and the detection of land use changes). And please address my comments in the manuscript.

Author(s) response 8

- *We are grateful for your constructive feedback on this matter. We have addressed the clarity issues on classification and change detection techniques in our Author responses 4 and 5 to reviewer #2 (correspondingly, in our revised manuscript) as well as in S3_Appendix. We have also included more references to support our methods, as requested. We have annexed Table 1 summarizing our responses reviewer comments in the manuscript, along with the reviewer comment, the change made, and the location of the change in the manuscript*.

**Response to Editor**

Editor comment 1

- Please ensure that your manuscript meets PLOS ONE's style requirements, including those for file naming. The PLOS ONE style templates

Author(s) response 1

- *We thank you for your valuable feedback. We have revised the manuscript based on the PLOS ONE’s journal requirements, as recommended specifically following the manuscript body formatting guidelines and Title, author, affiliations formatting guidelines accessed at* [*https://journals.plos.org/plosone/s/file?id=wjVg/PLOSOne_formatting_sample_main_body.pdf*](https://journals.plos.org/plosone/s/file?id=wjVg/PLOSOne_formatting_sample_main_body.pdf) *and* 
  [*https://journals.plos.org/plosone/s/file?id=ba62/PLOSOne_formatting_sample_title_authors_affiliations.pdf*](https://journals.plos.org/plosone/s/file?id=ba62/PLOSOne_formatting_sample_title_authors_affiliations.pdf)

Editor comment 2

- Did you know that depositing data in a repository is associated with up to a 25% citation advantage (<https://doi.org/10.1371/journal.pone.0230416>)? If you’ve not already done so, consider depositing your raw data in a repository to ensure your work is read, appreciated and cited by the largest possible audience. You’ll also earn an Accessible Data icon on your published paper if you deposit your data in any participating repository (<https://plos.org/open-science/open-data/#accessible-data>).

Author(s) response 2

- *Thank you for your information on the benefits of depositing raw data in a repository. However, almost all of raw data used for this study can be publicly accessed. The ERSS/LSMS-ISA datasets are available in the public domain: Ethiopia Socioeconomic Survey (ESS) 2011-2012, Wave 1,* [*http://dx.doi.org/10.48529/80xt-9m68*](https://doi.org/10.48529/80xt-9m68)*; Ethiopia Socioeconomic Survey (ESS) 2013-2014, Wave 2,* [*http://dx.doi.org/10.48529/mccp-y123*](https://doi.org/10.48529/mccp-y123)*; Ethiopia Socioeconomic Survey (ESS) 2015-2016, Wave 3,* [*http://dx.doi.org/10.48529/ampf-7988*](https://doi.org/10.48529/ampf-7988)*. The data accessed and filtered using the following criteria: saq01==1 & rural!=3, which correspond to selecting households in Tigray region that are engaged in farming activities in rural areas and small towns. Moreover, anyone can access LanSat imageries at* [*https://earthexplorer.usgs.gov/*](https://earthexplorer.usgs.gov/)

Editor comment 3

- In your Data Availability statement, you have not specified where the minimal data set underlying the results described in your manuscript can be found. PLOS defines a study's minimal data set as the underlying data used to reach the conclusions drawn in the manuscript and any additional data required to replicate the reported study findings in their entirety. All PLOS journals require that the minimal data set be made fully available. For more information about our data policy, please see <http://journals.plos.org/plosone/s/data-availability>.

Author(s) response 3

- *We clearly understand your valid concern on the minimal dataset and thank you for that. The data that support the findings of this study are available as supporting information files (zipped file). The files in the zipped data file are named as follows: min_data_hh_features.dta, min_data_plot_by_field_features.dta, min_data_land_use_output_price.dta, min_data_mixed_reg_data_diag_block.dta, min_data_lulc_perception_follow_up_survey.dta, and min_data_lulc.dta. A metadata named metadata (DOCX) describing contents and the source of the data of each file is also attached. The data are also available from the corresponding author upon reasonable request.*

Editor comment 4

- We note that Figures 1, 2a and 2b in your submission contain [map/satellite] images which may be copyrighted. All PLOS content is published under the Creative Commons Attribution License (CC BY 4.0), which means that the manuscript, images, and Supporting Information files will be freely available online, and any third party is permitted to access, download, copy, distribute, and use these materials in any way, even commercially, with proper attribution. For these reasons, we cannot publish previously copyrighted maps or satellite images created using proprietary data, such as Google software (Google Maps, Street View, and Earth). For more information, see our copyright guidelines: <http://journals.plos.org/plosone/s/licenses-and-copyright>.
   
  We require you to either (1) present written permission from the copyright holder to publish these figures specifically under the CC BY 4.0 license, or (2) remove the figures from your submission:

Author(s) response 4

- *We are grateful for your constructive feedback on this matter. Based on your recommendation we removed Figures 2a and 2b (land cover maps),* *which were not only had copyright issues but also depicting visualization of land cover changes analyzed in detail using trend change and transition matrix analysis, hence were redundant. However, with regard to Figure 1 we have learned that FEWS NET is already under the CC BY 4.0 license. Under its Data and information use and attribution policy [https://help.fews.net/fdp/data-and-information-use-and-attribution-policy], it declares any one is using data collected by FEWS NET in a visual, graphic, map or infographic of once own, it simply requires to attribute the source data clearly and prominently on the visual itself or with in the key/legend and include a link [**https://fews.net]. We indicated this on caption of Fig. 1 in lines 83-87. Enumeration area information is considered public information presented by the Ethiopian government agency, Central Statistics Agency (CSA) of Ethiopia and can be accessed in either of* [*http://microdata.worldbank.org/index.php/catalog/2053*](https://microdata.worldbank.org/index.php/catalog/2053)*,* [*https://microdata.worldbank.org/index.php/catalog/2247*](https://microdata.worldbank.org/index.php/catalog/2247)*, or* [*http://microdata.worldbank.org/index.php/catalog/2783*](https://microdata.worldbank.org/index.php/catalog/2783)

**Table 1. How we addressed reviewers’ feedback on the submitted manuscript**.

| **SN** | **Reviewer comment** | **Line number (on submitted manuscript)** | **Change made** | **Line number (on revised manuscript)** | **Justification** |
| --- | --- | --- | --- | --- | --- |
| 1 | Please add Ethiopia (on the title) | Line 1 | Added “Ethiopia” | Line 4 | Specifying where Tigray is found is reasonable. |
| 2 | Please add Ethiopia (on the abstract) | Line 12 | Added “Ethiopia” | Line 22 |  |
| 3 | Please write first the full word | Line 58 | Added the full name “Land Use Land Cover” | Line 52 |  |
| 4 | There is no full stop before this word. So, no capitalization needed | Line 70 | Word “we” rewritten to begin in lower case | Lines 69, 70 and 71 |  |
| 5 | Unclear what you mean (by three waves) | Line 71 | “three waves” replaced by “repeated measurements for the year 2012, 2014, and 2016” | Line 71-72 | We agree it may not be clear for every reader that waves represent rounds of data collection in panel datasets. |
| 6 | Noun is missing (on basin or sub-basin) | Line 73 | Added noun “river” | Line 74 |  |
| 7 | uncommented |  | Changed number of livelihood zones (LHZs) to 16 | Line 80, 90 | Error in counting the number of LHZs |
| 8 | But this map does not say anything about the data points, the names of administrative boundaries. | Line 85 | Removed “international and administrative boundaries” | From Fig. 1 caption Lines 83-87 | We are convinced that the aim is not to show international and administrative boundaries of Tigray region rather the location of Tigray, its livelihood zones and center of enumeration areas where ERSS-LSMS-ISA are collected |
| 9 | Please add the source in the reference list | Line 87 | Added a reference numbered “27” in reference list. | Line 611-612 |  |
| 9^1^ | Please write first the full word before you use an abbreviation -Figures should work as stand-alone | Line 87-88 | Added of FEWS NET at its first appearance and in Figure 1 captions | Line 86-87, 89 |  |
| 10 | Please provide more details in the reference list. For now, I could not find this paper. | Line 98 | Added the reference numbered “31” in reference list. | Line 620-623 | The reference was Tigray government report. For better search we replace it by WB report of same issues. |
| 11 | This information is by far not enough. Please provide more details about the identification of representative | Line 114 | Comment addressed in author response 3 to reviewer #2 | Line 115-141 |  |
| 12 | three times (on three waves) | Line 119 | Refer to #5 |  | Same to comment in SN 5. |
| 13 | How I can access the data? Otherwise, I cannot validate your data. | Line 121 | Added ways to access the LSMS-ISA data. | Line 130-133  Or in Author response 3 to editor |  |
| 14 | Please add the source (for US Geological Survey Center for Earth Resources Observation and Sciences) | Line 132 | Added the source | Line 152 |  |
| 15 | Please write which radiometric and atmospheric corrections and how. | Line 133 | Added specific radiometric and atmospheric correction and explained how | Lines 152-156 |  |
| 16 | Please write the full word before you use an abbreviation. Captions/Figures should work as stand-alone | Line 136 (Table 1) | Added the full name “Land Use Land Cover” | Line 157 (title of Table 1) |  |
| 17 | Where does this definition come from? (source) | Line 136 (Table 1) | Added justification for the operational definition | Lines 144-147 |  |
| 18 | River are not bare land | Line 136 (Table 1) | Replaced “river course” by “rocks found along river banks” | Definition of Bare land in Table 1 (line 157) |  |
| 19 | How do you distinguish this from bare land? | Line 136 (Table 1) | Removed “unidentified” | Definition of Other land use land cover in Table 1 (line 157) | Other land use land cover were areas covered by water, wetlands, and settlement |
| 20 | Please add source | Line 138-139 | Refer to #9 |  | Same to comment in SN 9. |
| 21 | How many and from which years | Line 143-144 | Added year for toposheets | Line 172 |  |
| 22 | References are missing (on class iterative self-organizing data analysis) | Line 149 | Added the reference numbered “40” | Line 168 |  |
| 23 | When (year, amount and season) and how data were collected (on ground truthing field observation data collected) | Line 150 | Added more clarifications on ground truthing | Lines 170-179 |  |
| 24 | The same as the field observations (on sample points) | Line 153 | Refer to #23 |  |  |
| 24’ | Please add references of used methods and similar methodological approaches to justify why you have used this process | Line 154 | Added the references numbered “42” and “43” | Lines 181, 182 |  |
| 25 | This does not match with your objective: Detailed analysis of LULC change and its drivers in Tigray. | Line 159 | Comment addressed in author response 3 to reviewer #2 |  |  |
| 26 | This source is quite old. Is there no updated literature? | Line 164 | Added the updated reference numbered “47-49” | Line 203 |  |
| 27 | How the sentence is written, it is not clear that these references are not specifically for Ethiopia. | Line 178 | Rephrased the paragraph for clarity, references specific to Ethiopia cited. | Lines 208 - 214 | We agree that the sentences were fragmented and too long making the paragraph unclear. We simplified and clearly rewrite the paragraph. Citations are now specific to Ethiopia. |
| 28 | Was this defined before | Line 195 | Added the definition for sustainability group as “The number of observations of a plot in all the three panel rounds in a specific land use category.” | Lines 229 - 234 | We are convinced that definitions of important terms should also be defined in the main manuscript rather than defining them only in Appendices. |
| 29 | What is “waves plots are observed in”? | Line 197 | Added the clarification “how many times (rounds) plots have been assigned to a specific land use category during 2012 to 2016” | Lines 230-232 |  |
| 29^1^ | A reference where this has happened already? | Line 201 | Added references numbered “55, 62, 63” | Line 241 |  |
| 29^2^ | References are missing | Line 213 or Line 189 | Added reference numbered “45” | Line 224 |  |
| 30 | Why? Where do I see reference? | Line 202-203 | Added the citation numbered “64, 65” | Line 242 |  |
|  | Are these your own results? Where are the results shown (Table)? Or do not refer here to table 2 if these Results are not shown there. | Line 241 | Added Table 2 with the relevant information | Line 288 |  |
| 31 | What is the difference to the soil quality classes and how these classes were defined | Line 255 (Table 2) | Added definitions of soil quality and soil type in the caption for Table 2. | Line 292 – 293  (Table 2 caption) |  |
| 31^1^ | Where did you get this information from? (This is not possible from the LandSat data.) | Line 255 (Table 2) | Added the source for geo-referenced geospatial data used | Line 129-130 | We agree with the reviewer that information on soil quality cannot be obtained from LandSat. However, the ERSS/LSMS-ISA links plot and community level geo-referenced geospatial data to encourage researchers to use the dataset. |
| 32 | References are missing | Line 265-266 | Added the reference number “72” | Line 300 |  |
| 33 | Please explain these abbreviations. A table should be understandable without reading the main body of the text. | Line 290 | Added the full names of each Livelihood zones in the caption for Table 4 | Line 319-320 |  |
| 34 | What is it (on EPL) | Line 299 | Added the full name of EPL “Eastern Plateau” | Line 326 |  |
| 35 | Did you introduce these abbreviations before? | Line 306 | Added the full names abbreviations at their first appearance | Lines 332, 337, 388, |  |
| 36 | I don’t know which section do you mean | Line 321 | Deleted reference to Section 3.1.1 |  | It was a merely error while editing our manuscript. |
| 37 | But if you leave land fallow, the land can regenerate. This is contradicting to “damage” the environment | Line 472 | Removed “environment” |  | We agree with the reviewer that leaving land fallow can help the land regenerate. We have deleted the word “environment” |
| 37^1^ | References are missing | Line 737 | Added references numbered “1,2,3 4” | Line 14, S2_Appendix |  |
| 37^2^ | References are missing | Line 748 | Added references numbered “3, 4,5” | Line 24, S2_Appendix |  |
| 37^3^ | References are missing | Line 775 | Added references numbered “7,8” | Line 49, S2_Appendix |  |
| 37^4^ | References are missing | Line 800 | Added references numbered “9” | Line 73, S2_Appendix |  |
| 37^5^ | Please explain the abbreviations (a legend or info in the caption about the abbreviations is missing) | Figure 5 | Added full name of all abbreviations in the figure caption in Fig. 4 | Line 401-407 |  |
| 38 | I am not sure if extensive footnotes are recommended for this journal. | Line 951 | Removed footnotes from the main text of manuscript. |  |  |

We summarized our responses to the reviewer’s comments that were not included in the email, but only in the manuscript as annotations. These comments mainly concerned definition of terms (clarifications); style, grammar, and formatting issues, such as punctuation, spelling; references; acronyms; and word choices. Comments with SN 1 up to 38 are of same reviewer (more likely reviewer #1), while comments with SN with superscripts are of another reviewer (more likely reviewer #2). We have labeled different SN because we are not sure whose comments are (reviewer #1 or reviewer #2) as they are inserted as comments in the submitted manuscript with no identification of reviewers.
