# Supplementary material for: Multi-level determinants of land use land cover change in Tigray, Ethiopia: A mixed-effects approach using socioeconomic panel and satellite data
Source: PLoS One. 2024 Jun 13;19(6):e0304896. doi: 10.1371/journal.pone.0304896 (PMC11175475; doi:10.1371/journal.pone.0304896)
Supplement: Attachment — Submitted filename: Response to Reviewers.docx [file pone.0304896.s013.docx]

Dear Dr. Josily Samuel,

We are grateful for your and reviewer constructive feedback on our manuscript entitled “Multi-level determinants of land use land cover change in Tigray, Ethiopia: A mixed-effects approach using socioeconomic panel and satellite data” (manuscript number PONE-D-23-33020), submitted to PLOS ONE.

We appreciate your consideration of our work for revision. We have revised the manuscript according to your comments and addressed all your concerns.

This letter explains how we have revised our manuscript in response to each of your comments. We hope that our revisions meet your expectations, and our manuscript is now ready for publication in PLOS ONE.

We have prepared a point-by-point response to each issue raised by you and the reviewer. We have also listed the changes that we have made in the revised manuscript, which are marked in blue.

Please find below our detailed responses to you and the reviewer, followed by the list of changes that we have made in the revised manuscript.

Moreover, we have specified in our data availability statement in the previous revision that the minimal data supporting the study are available as supporting information files uploaded as zipped file. If there is still room to change this, we have published the minimal data in Dryad repository at <https://doi.org/10.5061/dryad.n5tb2rc2z>

Sincerely,

Tadele Tafese Habte

Centre for Environment and Development, College of Development Studies, Addis Ababa University, Addis Ababa, Ethiopia

We thank you and the reviewer for your valuable feedback and your time and effort in reviewing our manuscript. We hope that you will find our revisions satisfactory and accept our manuscript for publication in PLOS ONE.

**Response to Reviewer #3**

Reviewer comment 1

- Line no. 79-80 Agro-climatic zone is a land unit in terms of major climates, suitable for a certain range of crops and cultivars but the name suggests it is based on elevation. Please clarify.

Author response 1

- *Thank you for your question. We agree that the agroclimatic zonation we used is specific to Ethiopia and may not be applicable to other regions. The agroclimatic background of the region is based on the traditional zonation that reflects the major physical conditions and agricultural land uses according to altitude and crop patterns. Elevation is a key factor that influences temperature and rainfall in Ethiopia. This traditional zonation has been validated by several studies, such as Hurni (1998), who defined the following zones:*
  - *Bereha: hot lowlands below 500 meters, with limited crop production in the arid east and root crops and maize in the humid west.*
  - *Kolla: lowlands between 500 and 1,500 meters, with sorghum, finger millet, sesame, cowpeas, and groundnuts as predominant crops.*
  - *Woina Dega: midlands between 1,500 and 2,300 meters, with wheat, teff, barley, maize, sorghum, chickpeas and haricot beans as predominant crops.*
  - *Dega: highlands between 2,300 and 3,200 meters, with barley, wheat, highland oilseeds, and highland pulses as predominant crops.*
  - *Wurch: highlands between 3,200 and 3,700 meters, with barley as a common crop.*
  - *Kur: highland areas above 3,700 meters, mainly used for grazing pasture.*

*The dominant agroclimatic zones in Tigray are: Kolla (lowlands), Woina Dega (midlands) and Dega (highlands).*

Reviewer comment 2

- Background - should also include the gap in research which this paper is addressing with the objectives.

Author(s) response 2

- *Thank you for your valuable feedback. We appreciate your attention to detail. In response to your suggestion, we have now incorporated a paragraph in the background section (lines 111 to 117) that explicitly outlines the research gap this paper aims to address. This addition will provide readers with a clearer understanding of the context and objectives of our study.*

Reviewer comment 3

- Methodology- can you show the whole methodology in flowchart form. It will make the reader to understand the whole thing better.

Author(s) response 3

- *Thank you for your feedback. We have addressed your suggestion by including a flowchart that visually represents the methodology. The flowchart has been added in lines 205 to 206. Additionally, the caption for this flowchart, which is included as supporting information, can be found in the supporting information list at lines 789 to 790. This should enhance the reader’s understanding of the entire process.*

Reviewer comment 4

- Line no. 170- Change the word to Classification from calcification.

Author(s) response 4

- *Thank you for your observation. We have addressed your comment by replacing “Calcification” with “Classification” in line 176.*

Reviewer comment 5

- Line no. 171- Please tell the year in which you have interviewed the 34-key informant.

Author(s) response 5

- *Thank you for your feedback. We appreciate your attention to detail. The key informant interviews with elderly individuals were conducted in August 2019, during the ERSS/LSMS-ISA follow-up survey. As a result, we have now included the year in line 182 to provide clarity.*

Reviewer comment 6

- Line no. 178- A total of 375 sample points. It is not sampling points it is training points for supervised classification to create signature file.

Author(s) response 6

- *Thank you for your comment. The term “sampling points” has been revised to “training points” in line 184 to accurately reflect the purpose of these.*

Reviewer comment 7

- Line no. 281- crop farming can be simply written as farming.

Author(s) response 7

- *Thank you for your valuable feedback. Based on your suggestion, we have replaced “crop farming” with the more concise term “farming” in line 287.*

Reviewer comment 8

- As far as I understood, for LULC you have taken data of 1986 and 2016, while for socio economic study the data is of 2012 to 2016.

Author(s) response 8

- *Thank you for your valid concern. We have used land cover data for two time points: 1986 and 2016.* *We have corrected all the instances where we mistakenly wrote “1986 to 2016” instead of “1986 and 2016” when describing the data we used. Please see lines 428 and 472 for the changes.*

Reviewer comment 9

- It is not vertosol, I think its soil order vertisols. Please rewrite the sentence.

Author(s) response 9

- *We appreciate your observation. You are correct, the soil order is vertisols, not vertosol. We have revised the sentence accordingly in Table 2 and 4 and line numbers 289, 312, and 441.*

Reviewer comment 10

- Line no. 298- STATA is same the software name you mentioned earlier? If so maintain the same format.

Author(s) response 10

- *We thank you for your attention to detail. You are right, the software name is Stata, not STATA. We have corrected this typo in line 304 and ensured consistent formatting throughout the manuscript.*

Reviewer comment 11

- Line no. 401- if you have used 1986 data and 2016 do not write 1986-2016, instead write 1986 and 2016.

Author(s) response 11

- *We are grateful for your observation. This is the same issue as in response 8. We have changed “1986 to 2016” to “1986 and 2016” in line 428 and elsewhere as appropriate.*

**Response to Editor**

Editor comment 1

- Please review your reference list to ensure that it is complete and correct. If you have cited papers that have been retracted, please include the rationale for doing so in the manuscript text, or remove these references and replace them with relevant current references. Any changes to the reference list should be mentioned in the rebuttal letter that accompanies your revised manuscript. If you need to cite a retracted article, indicate the article’s retracted status in the References list and also include a citation and full reference for the retraction notice.

Author(s) response 1

- *We appreciate your comment and we have carefully reviewed our reference list. All the citations and references are complete and correct. We have used Zotero reference manager to manage our references. Zotero is linked to the Retraction Watch Database and automatically checks for retracted articles with DOI. For the references without DOI, we manually searched the Retraction Watch Database using Author(s) and Title as filtering criteria. We did not find any retracted references in our list. Therefore, we did not need to cite any retracted article or provide any rationale for doing so.*
