# Supplementary material for: Multi-level determinants of land use land cover change in Tigray, Ethiopia: A mixed-effects approach using socioeconomic panel and satellite data
Source: PLoS One. 2024 Jun 13;19(6):e0304896. doi: 10.1371/journal.pone.0304896 (PMC11175475; doi:10.1371/journal.pone.0304896)
Supplement: Attachment — Submitted filename: Response to Reviewers.docx [file pone.0304896.s014.docx]

Dear Dr. Josily Samuel,

We are grateful for your and reviewer constructive feedback on our manuscript entitled “Multi-level determinants of land use land cover change in Tigray, Ethiopia: A mixed-effects approach using socioeconomic panel and satellite data” (manuscript number PONE-D-23-33020), submitted to PLOS ONE. We appreciate your consideration of our work for revision.

This letter explains how we have revised our supplementary information named S1_flowchart in response to the reviewer’s comments. We hope that our revisions meet your expectations, and our manuscript is now ready for publication in PLOS ONE.

We have prepared a point-by-point response to each issue raised by you and the reviewer. We have also listed the changes that we have made in the revised supplementary information.

Please find below our detailed responses to you and the reviewer, followed by the list of changes that we have made.

We thank you and the reviewer for your valuable feedback and your time and effort in reviewing our manuscript. We hope that you will find our revisions satisfactory and accept our manuscript for publication in PLOS ONE.

Sincerely,

Tadele Tafese Habte

Centre for Environment and Development, College of Development Studies, Addis Ababa University, Addis Ababa, Ethiopia

**Response to Reviewer #3**

Reviewer comment 1

- The reviewer put comments [hand written] on the S1_flowchart attached to email and requested to revise accordingly.

Author response 1

- *Thank you for your comments on the flowchart summarizing the methodology. In response to the reviewer’s clear and direct feedback, the S1_flowchart has been revised to incorporate the suggested additions and deletions. The concepts indicated by the reviewer have been integrated, and unnecessary elements have been removed as per the guidance provided. Specifically;*
  - *We have clarified the types of data utilized:* ***socioeconomic*** *and* ***satellite data****, and have removed questioning data availability. Additionally, we have detailed the two sources of socioeconomic data used, namely* ***primary*** *and* ***secondary*** *data sources.*
  - *In response to the comment on land use classification, we acknowledge the hybrid approach taken in our methodology. Initially, we employed unsupervised techniques for class identification, using Iterative Self-Organizing Data Analysis clustering to group pixels based on spectral similarities. Subsequently, ground control points (GCPs) collected via high-resolution imagery from Google Earth and GPS coordinates served as training data for a supervised Maximum Likelihood Classification (MLC) algorithm. This refined the land use categories with established spectral signatures. Although our classification process involved these stages, the final supervised MLC algorithm determined our classification. Therefore, we have specified the application of supervised image classification in the revised flowchart.*
  - *We appreciate your concern regarding the temporal data representation.* *We have corrected the temporal data representation from “1986 to 2016” to the specific time points: 1986 and 2016, as used in our manuscript.*
  - *We concur that the back loop to the research question is redundant once the research question has been addressed. The flowchart has been updated to remove this loop, aligning with the recommendation.*

*We are committed to ensuring the highest quality of our work and are open to further revisions should there be additional feedback.*

**Response to Editor**

Editor comment 1

- Please review your reference list to ensure that it is complete and correct. If you have cited papers that have been retracted, please include the rationale for doing so in the manuscript text, or remove these references and replace them with relevant current references. Any changes to the reference list should be mentioned in the rebuttal letter that accompanies your revised manuscript. If you need to cite a retracted article, indicate the article’s retracted status in the References list and also include a citation and full reference for the retraction notice.

Author(s) response 1

- *We thank you for emphasizing the importance of an accurate and reliable reference list. Following your advice, we have conducted a careful review of our references using Zotero, integrated with the Retraction Watch Database, to automatically check for retracted articles with DOIs. Additionally, we performed manual searches for references without DOIs. We can confirm that our reference list is complete, accurate, and free of retracted articles. We are prepared to make any further adjustments if necessary.*
